# Cytotoxic Cyclolignans Obtained by the Enlargement of the Cyclolignan Skeleton of Podophyllic Aldehyde, a Selective Podophyllotoxin-Derived Cyclolignan

**DOI:** 10.3390/molecules29071442

**Published:** 2024-03-23

**Authors:** Pablo A. García, Ángela-Patricia Hernández, Mª Antonia Gómez-Zurita, José M. Miguel del Corral, Marina Gordaliza, Andrés Francesch, Arturo San Feliciano, Mª Ángeles Castro

**Affiliations:** 1 Departamento de Ciencias Farmacéuticas, Área de Química Farmacéutica, Facultad de Farmacia, CIETUS/IBSAL, University of Salamanca, Campus Miguel de Unamuno, 37007 Salamanca, Spain; angytahg@usal.es (Á.-P.H.); marintogzf@gmail.com (M.A.G.-Z.); jmmcs@usal.es (J.M.M.d.C.); mliza@usal.es (M.G.); artsf@usal.es (A.S.F.); 2 Department of Medicine and General Cytometry Service-Nucleus, CIBERONC CB16/12/00400, Cancer Research Centre (IBMCC/CSIC/USAL/IBSAL), 37007 Salamanca, Spain; 3 PharmaMar S.A., Avda de los Reyes, 1, 28770 Colmenar Viejo, Spain; afrancesch@pharmamar.com; 4 Programa de Pós-Graduação em Ciências Farmacêuticas, Universidade do Vale do Itajaí, UNIVALI, Itajaí 88302-901, SC, Brazil

**Keywords:** natural products, podophyllotoxin, podophyllic aldehyde, nucleophilic addition reactions, anticancer, cytotoxicity, selectivity

## Abstract

Podophyllotoxin, a cyclolignan natural product, has been the object of extensive chemomodulation to obtain better chemotherapeutic agents. Among the obtained podophyllotoxin derivatives, podophyllic aldehyde showed very interesting potency and selectivity against several tumoral cell lines, so it became our lead compound for further modifications, as described in this work, oriented toward the enlargement of the cyclolignan skeleton. Thus, modifications performed at the aldehyde function included nucleophilic addition reactions and the incorporation of the aldehyde carbon into several five-membered rings, such as thiazolidinones and benzo-fused azoles. The synthesized derivatives were evaluated against several types of cancer cells, and although some compounds were cytotoxic at the nanomolar range, most of them were less potent and less selective than the parent compound podophyllic aldehyde, with the most potent being those having the lactone ring of podophyllotoxin. In silico ADME evaluation predicted good druggability for most of them. The results indicate that the γ-lactone ring is important for potency, while the α,β-unsaturated aldehyde is necessary to induce selectivity in these cyclolignans.

## 1. Introduction

Natural products constitute a good source of bioactive compounds that can be used directly as drugs or, most frequently, as hits for developing new drugs. Also, natural products without any activity reports have often served as starting materials for the preparation of bioactive derivatives [1,2,3]. We usually refer to the concepts of improving and inducing bioactivity by chemical transformations as the chemomodulation and chemoinduction of bioactivity, respectively [4]. For many years, our research group has been involved in both the chemomodulation and chemoinduction of the bioactivity of natural compounds, such as cyclolignans, isolated from their natural sources.

**Figure 1:**
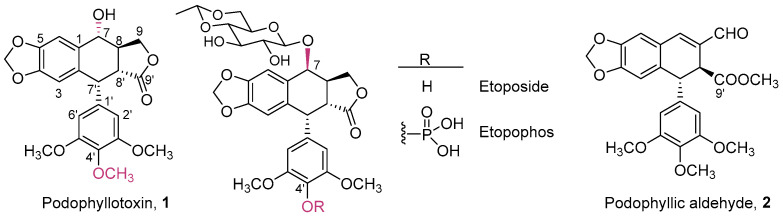
Chemical structures of podophyllotoxin and several derivatives, with the IUPAC numbering system used in this work.

Cyclolignans belong to the lignan family of natural compounds, which are widely distributed in Nature. Many biological activities have been described for them, such as anthelmintic, antiviral, or anticancer effects [5]. Among lignans, podophyllotoxin stands out for its use as an antiviral in the treatment of venereal warts and, principally, as the starting material for obtaining the clinically used anticancer drugs etoposide and etopophos (Figure 1) [6,7,8,9]. It is well known that both the natural compound and its semisynthetic derivatives have different mechanisms of action: while podophyllotoxin inhibits tubulin polymerization, etoposide and its analogs are inhibitors of the DNA-topoisomerase II enzyme. This change in the mechanism of action has been related to some structural changes in the cyclolignan skeleton, such as epimerization and glycosylation at C7 and *O*-demethylation at C4′ [10]. The numbering of the cyclolignan skeleton used in this work (Figure 1) is in accordance with IUPAC rules for lignans, which is based on the two phenylpropanoid biogenetic subunits whose condensation leads to lignans. Thus, one of the subunits is numbered from 1 to 9 and the other from 1′ to 9′ (Figure 1) [11].

**Figure 2:**
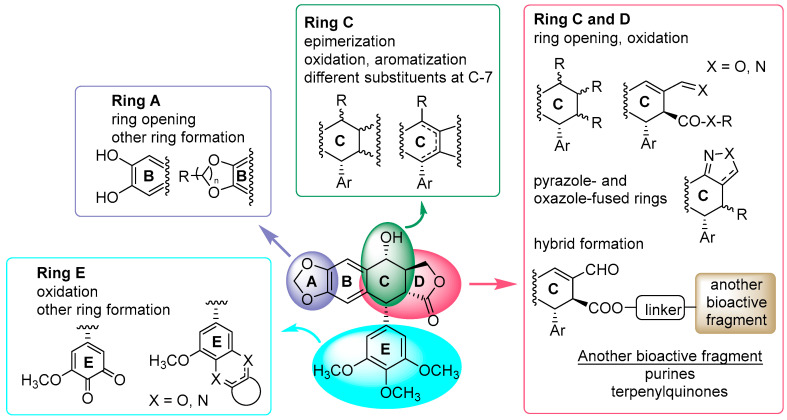
A schematic representation of the chemomodulation of podophyllotoxin performed by our group.

During the last decades, extensive chemomodulation of podophyllotoxin, **1**, has been performed by our research group, modifying nearly all the rings and functions of the cyclolignan skeleton [4], including hybridization with other biologically active natural compound derivatives, such as purines [12] or terpenylhydroquinones [13]. Such transformations are summarized in Figure 2 and had a variable influence in terms of structure–activity relationship (SAR) analysis. Most of the derivatives retained cytotoxicity at the micromolar level, with several differences. Noteworthy was the opening of the A-ring and the formation of pyrazole- and oxazole-fused rings, affecting the C- and D-rings, which showed interesting immunosuppressive activity either in vitro or in vivo [14], although they were less cytotoxic than podophyllotoxin. Also, hybrids formed by the union with another bioactive fragment, through position C7, C9, or C9′, led to promising agents with dual mechanisms of action [13].

Among the large number of podophyllotoxin derivatives obtained by our group, special mention should be made of podophyllotoxin-derived cyclolignans lacking the lactone ring, such as podophyllic aldehyde (**2**, Figure 1) and its imine derivatives, which showed selective cytotoxicity against certain human tumor cells, so **2** became our lead compound for further modifications. The antitumor evaluation of podophyllic aldehyde derivatives indicated that they kept the same mechanism of action as podophyllotoxin, and the α,β-unsaturated aldehyde function was important for selectivity, while the nature and size of the chain attached to the carboxylic acid at C9′ had variable effects on cytotoxicity and selectivity, with evidence of a possible third mechanism of action for podophyllotoxin-related lignans [15,16].

As a continuation of our research related to the chemomodulation of the bioactivity of cyclolignans, and with the aim of emphasizing the importance of the bioactivity of an untouched α,β-unsaturated aldehyde function at C9, in this work, we describe further chemical modifications performed at this aldehyde function, oriented toward the enlargement of the cyclolignan skeleton and its influence on bioactivity. They include the formation of vinylogues, reactions with different nucleophiles, and the incorporation of the aldehyde carbon into heterocyclic rings at the C2 position of several five-membered rings, such as thiazolidin-4-one and benzo-fused azoles (benzoxazole, benzothiazole, benzimidazole), purine, and 1-deazapurine. Subsequent cytotoxicity assays of the obtained derivatives were performed on three types of human cancer cells.

## 2. Results and Discussion

### 2.1. Chemistry

Podophyllic aldehyde, **2**, was obtained from podophyllotoxin, **1**, isolated from the rhizome resin of *Podophyllum emodi* [17], following the procedure previously described by us [15]. To achieve our objective of producing new bioactive podophyllic aldehyde derivatives, several carbon and heteroatom nucleophiles were used, taking advantage of the electrophilic character of the aldehyde function and the easiness of nucleophilic addition reactions.

#### 2.1.1. Addition of Carbon Nucleophiles

**Scheme 1:**
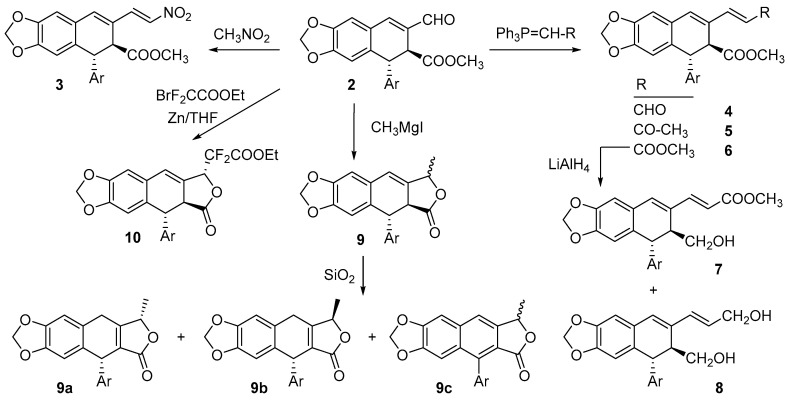
Reactions of podophyllic aldehyde, **2**, with C-nucleophiles.

Several vinylogous-like derivatives of **2** were prepared by a reaction with nitromethane and through Wittig reactions to obtain the extended derivatives **3**–**8** (Scheme 1).

The nitro derivative **3** was obtained by a reaction with nitromethane in the presence of ammonium acetate. When the reaction was performed in glacial acetic acid as a solvent under reflux [18], the unreacted aldehyde **2** was recovered. However, when nitromethane was used as a reagent and as a solvent [19], the nitro derivative **3** was obtained in low yield (7%), also recovering the unreacted aldehyde **2** (39%). HMBC and HMQC NMR experiments confirmed the structure of **3** (Figures S3 and S4 in Supplementary Materials).

Analogs **4**–**6** were obtained through the Wittig reaction using triphenyl phosphonium ylides stabilized by carbonyl groups at the α-position. When (thiphenylphosphoranylidene)acetaldehyde was used, an irresolvable mixture of the starting aldehyde and vinylogue **4** was obtained. A careful analysis of the NMR spectra of the mixture allowed us to identify the signals corresponding to the extended aldehyde **4**.

When (thiphenylphosphoranylidene)propan-2-one and methyl (thiphenylphosphoranylidene)acetate were used, the corresponding compounds **5** and **6** were obtained in high yields (82% and 84%, respectively). Their structures were confirmed by HRMS and NMR spectra.

The reduction of the diester **6** with lithium aluminum hydride at a low temperature led to a mixture of **7** and **8**. For compound **7**, only the ester at C9′ was reduced under such conditions, while in compound **8**, both ester groups were reduced, without the alteration of the double bond in any of them, as deduced from their NMR data.

Grignard and Reformatsky reagents were also used as C-nucleophiles (Scheme 1). Thus, the reaction of **2** with methylmagnesium iodide led to a complex reaction product, in whose ^1^H and ^13^C NMR spectra were signals assignable to the olefinic proton and carbon at position C7 (6.34 and 119 ppm approx., respectively), while signals for the aldehyde function were absent, as were those of the methyl ester at C9′ (around 3.60 and 55 ppm). Instead, two doublets at 1.63 and 1.54 ppm were present. All of these data indicated the addition of the organomagnesium compound to the aldehyde with relactonization [20] to give a mixture of isomers, **9**. Chromatography of the reaction product led to the isolation of small amounts of compounds **9a**–**c**. Compounds **9a** and **9b** were epimers at C9, in which the migration of the Δ^7^ double bond toward Δ^8(8′)^ occurred during chromatography, while in **9c**, the aromatization of the C-ring took place. The configuration of C9 in **9a** and **9b** was defined through nOe (nuclear Overhauser effect) NMR experiments. Thus, a positive nOe effect for compound **9a** between the methyl at C9 and the aromatic protons of the pendant trimethoxyphenyl ring indicated an *S* configuration for carbon C9. The absence of such an nOe effect for **9b** allowed us to assign the *R* configuration to **9b**.

When **2** was treated with ethyl bromodifluoroacetate in the presence of activated zinc dust in THF under reflux, compound **10** was isolated from the reaction product as a single epimer, in which not only did condensation with the aldehyde group take place, but relactonization also occurred again. The structure and configuration of **10** were determined after the analysis of its NMR spectra, 2D NMR experiments, and nOe effects. In this reaction, only epimer **10** was detected, probably because the additional coordination of zinc with the C9′-β-ester made the nucleophile approach the zinc-chelated aldehyde intermediate via the less hindered side only.

The presence of two electronegative fluorine atoms in the reagent was important for the reaction progress. When the same reaction was performed with ethyl bromoacetate under the same reaction conditions, mainly unreacted aldehyde was recovered, and only a small amount of dehydrodeoxypodophyllotoxin [21], resulting from the reduction of the aldehyde with the subsequent relactonization and aromatization of the C-ring, was detected.

#### 2.1.2. Addition of Nitrogen Nucleophiles

In our previous research, several N-nucleophiles, namely, substituted aromatic and aliphatic hydrazines, hydroxylamines, and amines, were used to obtain the corresponding hydrazones, oximes, and imines, for which very good results in potency and selectivity were obtained, particularly for the imine derivatives [22,23]. In those works, some of the aromatic amines used to form previous imines contained another heteroatom (N, O, S), located at the ortho position of the amine group, that can also act as a second nucleophile and add to the imine double bond. We describe here this kind of transformation to achieve the corresponding benzo-fused azoles that incorporate the C9 carbon of the cyclolignan skeleton into the additional five-membered heterocycle formed.

**Scheme 2:**
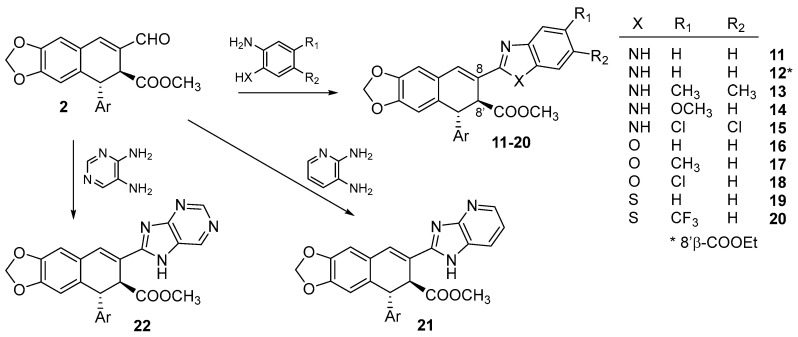
Synthesis of benzo-fused azoles **11**–**22** from **2**.

Thus, **2** was made to react with 1,2-phenylendiamines to obtain benzimidazoles **11**–**15**, with moderate to good yields. When the reaction with 1,2-phenylendiamine itself was performed in refluxing ethanol and acidic conditions, a transesterification reaction occurred, and the ethylester **12** was obtained in moderate yield. To prevent transesterification, acidic conditions were avoided, and instead, an oxidant was added to facilitate the final aromatization of benzimidazoles **11**–**15**, which were then obtained with better yields. When *o*-aminophenols or *o*-aminothiophenols were used as nucleophiles, the corresponding benzoxazoles **16**–**18** and benzothiazoles **19**–**20** were obtained in moderate yields (Scheme 2).

Diaminopyridine and diaminopyrimidine were also used as nucleophiles, leading, in these cases, to the 1-deazapurinyl and purinyl derivatives **21** and **22**, respectively (Scheme 2).

**Scheme 3:**
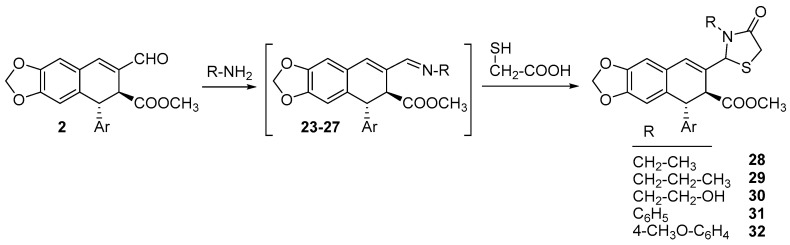
Synthesis of thiazolidines **28**–**32**.

With the aim to enlarge the type of heterocycles attached to C8, several 1,3-thiazolidin-4-one derivatives, substituted at the nitrogen atom, were also obtained from the condensation of **2** with several amines and the subsequent reaction of the formed imines **23**–**27** with thioglycolic acid to yield thiazolidinones **28**–**32** as mixtures of epimers at C9 (Scheme 3), as deduced from the presence of several duplicated signals in their NMR spectra (Figures S38–S44 in Supplementary Materials).

The formation of thiazolidinones **28**–**32** occurs in two steps: first the formation of the corresponding imines **23**–**27**, followed by condensation with thioglycolic acid. So, it can be carried out either in one pot, with the addition of thioglycolic acid to the reaction flask after the imine is formed, or with the previous isolation of the corresponding imine and further reaction with thioglycolic acid. For the latter, the corresponding imines **23**–**27** were prepared following the procedure described by us [23]. Thus, thiazolidinone **29** was obtained by both procedures with similar yields, while thiazolidinone **30** was only obtained in one pot, and thiazolidinones **28**, **31**, and **32** were obtained by the two-step procedure, as specified in the Section 3.1.

### 2.2. Bioactivity

**Figure 3:**
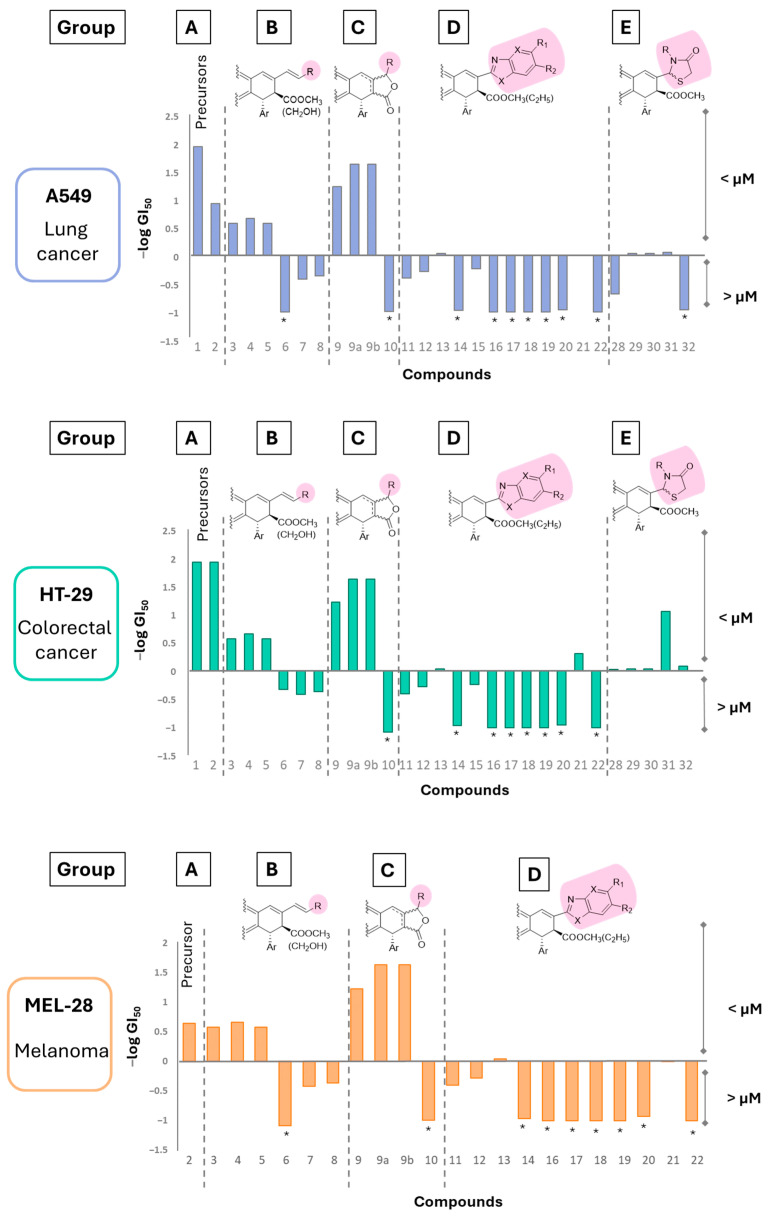
Cytotoxicity data (-log GI_50_) for the evaluated cyclolignans against the three tumor cell lines tested. Positive values correspond to the most cytotoxic compounds (GI_50_ < 1 µM), while negative values refer to lower cytotoxicity (GI_50_ > 1 µM). Values marked with asterisks (*) represent the maximum concentration tested. Groups are the following: A: precursors (**1** and **2**), B: C9 vinylogous derivatives (**3**–**8**), C: lactone derivatives (**9**–**10**), D: benzoheteroazoles (**11**–**20**), and E: thiazolidinones (**28**–**32**).

The cytotoxicity of most compounds was evaluated in vitro, using the colorimetric sulforhodamine B (SRB) method, to determine their in vitro antiproliferative activity against a panel of three human tumor cell lines: A549 (non-small-cell lung carcinoma), HT-29 (colon adenocarcinoma), and MEL-28 (malignant melanoma). The results are shown in Figure 3 and in Table S1 (GI_50_ values in µM). The natural compound podophyllotoxin, **1**, and podophyllic aldehyde, **2**, were included as references. In Figure 3, the 0 value on the y-axis coincides with the GI_50_ value of 1 µM (values are expressed as -log GI_50_) so that positive values correspond to cytotoxicity values below the micromolar level (higher cytotoxicity, GI_50_ < 1 µM) and negative values to lower cytotoxicity (GI_50_ > 1 µM). Each cell line is represented separately, with the figure itself indicating the different groups of compounds studied, arranged as follows: precursors (A: **1** and **2**), C9 vinylogous derivatives (B: **3**–**8**), lactone derivatives (C: **9**–**10**), benzoheteroazoles (D: **11**–**20**), and thiazolidinones (E: **28**–**32**).

From these results, several considerations of the structure–activity relationship can be deduced. It can be stated that many of them were cytotoxic, but in general, the selectivity toward a certain cell line disappeared, with a few exceptions.

Among the vinylogous derivatives, compounds **3**, **4**, and **5** retained cytotoxicity under the μM level, in the same range as their parent compound **2**, but with the loss of selectivity against HT-29 cells, while derivative **6** showed a certain selectivity, being fivefold more potent against HT-29 than against the other two cell lines tested, but with a lower potency, which was partially recovered in A549 and MEL-28 when the ester groups were reduced to the corresponding alcohols (**7** and **8** *vs*. **6**).

The best cytotoxicity results were observed for the 9-methyl lactones **9**, **9a**, and **9b**. These compounds showed GI_50_ values in the same range as that of **1** on the three cell lines tested and similar to that of **2** on HT-29 cells, but without the selectivity observed for podophyllic aldehyde between this cell line and the other two. However, the presence of a bulkier substituent at that position led to a considerable decrease in potency (**10** vs. **9**). It can also be observed that the mixture of epimers **9**, with a Δ^7^ double bond, was nearly three times less potent than the single isomers **9a** and **9b**, which had a Δ^8(8′)^ double bond, while no difference was observed between epimers (**9** *vs*. **9a** and **9b**).

Several differences can be observed among the evaluated benzazoles, which incorporated the C9 carbon into heterocyclic rings. Thus, benzimidazoles **11**–**15** retained cytotoxicity at the μM level, while benzoxazoles **16**–**18**, benzothiazoles **19**–**20**, and the purinyl derivative **22** were nearly inactive; only the deazapurinyl analog **21** kept the GI_50_ values at the μM level, with a slight selectivity against HT-29. Among the thiazolidinones, they also retained cytotoxicity at the μM level without selectivity toward any cell line, except in the case of those with aromatic substituents, **31** and **32**, which were tenfold more potent on HT-29 than on A549 cells. From this, it can be said that this skeleton enlargement did not improve the cytotoxicity of the molecules. In fact, the benzazole group includes the least potent compounds of all the series prepared.

In order to correlate the cytotoxic activity obtained with structural features, we conducted an in silico analysis focused on drug-likeness and ADME parameters. The structures were submitted to the freely accessible Swissadme platform [24] to predict their pharmacokinetic properties and possible adverse effects.

**Figure 4:**
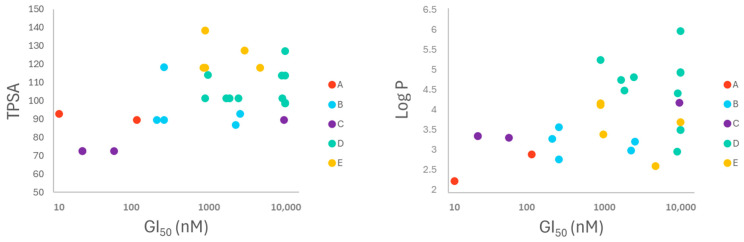
Graphical representation of log P and TPSA values of the cyclolignans, calculated by Swissadme platform. Log P and TPSA values are plotted with the cytotoxicity values of the compounds in A549. Color code for the different groups of compounds: red for precursors (A: **1** and **2**), blue for C9 vinylogous derivatives (B: **3**–**8**), purple for lactone derivatives (C: **9**–**10**), green for benzoheteroazoles (D: **11**–**20**), and yellow for thiazolidines (E: **28**–**32**).

Attending to their chemical features, compounds were organized into the same groups, A to E, indicated above, and a color code was assigned in the figures to each group to observe trends. Most of the compounds showed good druggability since they agreed with Lipinski’s rule of five. Groups A, B, and C (except compound **10**) fulfilled the five rules, and groups D and E only presented one violation. All the compounds also showed good non-rotatable bond (n-ROTB) values (<10) and acceptable H-bond acceptor values (7–10) (Table S2). Further correlations through in silico values were found when plotting TPSA and log S (referring to solubility) and log P (lipophilicity) in relation to cytotoxicity values. Figure 4 represents the values obtained for the A549 cell line (similar results were found for HT-29 cells and are shown in Figure S1). In both cases, a certain correlation among the groups is observed, which also follows a trend in terms of their cytotoxicity. At first glance, it can be seen that higher TPSA and log P values, relative to the precursors, correlate with lower cytotoxicity. Specifically, the most interesting in silico correlation was observed between log P and cytotoxicity. In this case, it can be seen that the values of groups B and E cluster together. Some components of these groups showed good cytotoxicity at the micromolar level. On the other hand, group D, which is generally less cytotoxic, clusters with higher log P values.

Also, the Swissadme platform provided very interesting information on other drug parameters. As can be seen in Figure S2, using the BOILED-egg model provided by the platform [25], most of the compounds were predicted to show good gastrointestinal absorption, and only compounds **9**, **9a**, and **9b** were predicted to passively permeate the blood–brain barrier. In terms of whether or not the compounds can be substrates of P-glycoprotein, the precursors appear to be substrates of this cellular efflux system. Similarly, the more cytotoxic compounds (**3**–**5** and **9**–**10**) seem to maintain this feature. In the case of metabolism, various isoforms of cytochrome P450 (CYP) were evaluated, with the results showing different patterns among the groups and the isoform considered (Table S3).

In summary, these results seem to indicate that the γ-lactone ring is important for the cytotoxic potency of these cyclolignans derived from podophyllotoxin, as has been previously considered [26]. In this sense, it is also worth mentioning that the majority of the podophyllotoxin derivatives described in the literature had this lactone ring [27,28] unsubstituted at C9, so little or nothing was known till now about the influence of that substitution on bioactivity. According to our results, it seems that a small substituent is tolerable at C9 but not a bulky one; in any case, it will be necessary to consider more examples in future works. Also, in accordance with some of our previous works, the α,β-unsaturated aldehyde seems necessary for the selectivity of the podophyllic aldehyde analogs [15,29] since the absence of the electrophilic character at C9 and the enlargement of the cyclolignan skeleton by its incorporation into different heterocycles led to less potent analogs or, in some cases, nearly inactive derivatives. These results warrant further studies aimed at understanding their mechanisms of action at the molecular level in different types of tumor cells.

## 3. Materials and Methods

### 3.1. Chemistry

NMR spectra were recorded on a Bruker (Billerica, MA, USA) WP 200 SY (200 and 50.3 MHz for ^1^H and ^13^C) or Bruker Avance 400DRX (400 and 100 MHz) spectrometer in CDCl_3_ using TMS as an internal reference. Chemical shift (δ) values are expressed in ppm and followed by multiplicity and coupling constants (J) in Hz. IR spectra were obtained on a Nicolet Impact 410 spectrophotometer in NaCl film. EM was run on a Hewlett-Packard (Palo Alto, CA, USA) 5890 Series II GC-MS with an electron impact ionization source. HRMS was run on a VG TS-250 spectrometer working at 70 eV and using electrospray ionization (ESI, Edinburgh, UK) or fast atom bombardment (FAB, Tokyo, Japan). Optical rotations were recorded on a Perkin-Elmer 241 polarimeter in a CHCl_3_ solution. UV spectra were obtained on a Hitachi (Tokyo, Japan) 100-60 spectrophotometer in an ethanol solution, and λ_max_ values are given in nm. Column chromatography (CC) purifications were performed using silica gel 60 (40–63 mm, 230–400 mesh, Merck), and TLC was carried out on silica gel 60 F_245_ (Merck, 0.25 mm thick). Solvents and reagents were purified by standard procedures as necessary.

Starting materials. Podophyllotoxin, **1**, was isolated from the rhizome resin of *Podophyllum emodi* (Berberidaceae) as previously described [17]. Podophyllic aldehyde, **2**, and imines **23**–**27** were obtained from **1** as described previously [23].

*Compound* **3**. A mixture of aldehyde **2** (100 mg, 0.24 mmol), ammonium acetate (23 mg, 0.3 mmol), and nitromethane (5 mL) was heated at reflux under an inert atmosphere for 4 h, chilled, and concentrated under vacuum. The residue was redissolved in CH_2_Cl_2_ and washed with brine, dried over Na_2_SO_4_, filtered, and evaporated. Purification by silica gel CC of the crude provided **3** (CH_2_Cl_2_/EtOAc 97:3, 8 mg, 8%) and unreacted **2** (CH_2_Cl_2_/EtOAc 9:1, 39 mg, 39%). Data for **3**: ^1^H NMR (CDCl_3_, 200 MHz) δ (ppm) 7.79 (*d*, 1H, H9, *J* = 13 Hz), 7.14 (*d*, 1H, H1″, *J* = 13 Hz), 7.08 (*s*, 1H, H7), 6.81 (*s*, 1H, H6), 6.69 (*s*, 1H, H3), 6.22 (*s*, 2H, H2′ and H6′), 6.01 (*d*, 1H, H10a, *J* = 1.3 Hz), 6.00 (*d*, 1H, H10b, *J* = 1.3 Hz), 4.58 (*d*, 1H, H7′, *J* = 1.5 Hz), 3.78 (*s*, 3H, H11′), 3.75 (*s*, 6H, H10′ and H12′), 3.69 (*s*, 3H, CH_3_O-9′), 3.55 (*d*, 1H, H8′, *J* = 1.5 Hz); ^13^C NMR (CDCl_3_) δ (ppm) 171.3 (C9′), 153.4 (C3′ an*d* C5′), 150.1 (C4), 147.4 (C5), 141.1 (C7),140.3 (C9), 137.6 (C1′), 137.2 (C4′), 136.2 (1″), 132.3 (C2), 125.8 (C1), 124.5 (C8), 109.9 (C3), 108.6 (C6), 104.4 (C2′ and C6′), 101.8 (C10), 60.8 (C11′), 56.2 (C10′ and C12′), 52.9 (CH_3_O-9′), 48.0 (C8′), 46.3 (C7′). IR ν_max_/cm^−1^ (film): 2923, 1731, 1604, 1586, 1504, 1460, 1376, 1242, 1126, 974.

The general procedure for the Wittig reaction. A mixture of aldehyde **2** and the corresponding triphenyl phosphonium ylide in dry toluene was stirred under an inert atmosphere at reflux for a specified time and then concentrated under vacuum. The reaction product was purified by CC on silica gel (CH_2_Cl_2_/EtOAc) to yield the corresponding vinylogous derivatives **4**–**6**.

*Compound* **4**. From **2** (140 mg, 0.33 mmol) and (thiphenylphosphoranylidene)acetaldehyde (155 mg, 0.49 mmol) in 60 mL of toluene for 7 d. CC on silica gel (CH_2_Cl_2_/EtOAc 96:4) of the crude yielded a 6:4 mixture of **2** and **4** (150 mg). Data for **4**: ^1^H NMR (CDCl_3_, 200 MHz) δ (ppm) 9.58 (*d*, 1H, H2″, *J* = 7.7 Hz), 7.26 (*d*, 1H, H9, *J* = 16 Hz), 6.98 (*s*, 1H, H7), 6.80 (*s*, 1H, H6), 6.70 (*s*, 1H, H3), 6.24 (*s*, 2H, H2′ and H6′), 6.16 (*dd*, 1H, H1″, *J* = 16 and 7.7 Hz), 6.00 (*d*, 1H, H10a, *J* = 1.1 Hz), 5.98 (*d*, 1H, H10b, *J* = 1.1 Hz), 4.57 (*d*, 1H, H7′, *J* = 1.5 Hz), 3.88 (*s*, 3H, H11′), 3.71 (*d*, 1H, H8′, *J* = 1.5 Hz), 3.74 (*s*, 6H, H10′ and H12′), 3.66 (*s*, 3H, CH_3_O-9′); ^13^C NMR (CDCl_3_) δ (ppm) 193.6 (C2″) 171.7 (C9′), 153.2 (C3′ and C5′), 152.9 (C9), 149.4 (C4), 147.3 (C5), 145.6 (C1′), 137.8 (C7), 137.0 (C4′), 131.3 (C2), 127.5 (C1″), 128.7 (C8), 126.1 (C1), 109.9 (C3), 108.3 (C6), 104.3 (C2′ and C6′), 101.6 (C10), 60.8 (C11′), 56.1 (C10′ and C12′), 52.7 (CH_3_O-9′), 47.4 (C7′), 46.4 (C8′). EM: 452 *m*/*z*.

*Compound* **5**. From **2** (100 mg, 0.23 mmol) and (triphenylphosphoranylidene)propan-2-one (76 mg, 0.24 mmol) in 20 mL of toluene for 2 d. CC of the crude on silica gel (CH_2_Cl_2_/EtOAc 9:1) yielded **5** (90 mg, 82%). ^1^H NMR (CDCl_3_, 200 MHz) δ (ppm) 7.30 (*d*, 1H, H9, *J* = 16 Hz), 6.92 (*s*, 1H, H7), 6.78 (*s*, 1H, H6), 6.68 (*s*, 1H, H3), 6.25 (*s*, 2H, H2′ and H6′), 6.13 (*d*, 1H, H1″, *J* = 16 Hz), 5.98 (*d*, 1H, H10a, *J* = 1.1 Hz), 5.97 (*d*, 1H, H10b, *J* = 1.1 Hz), 4.54 (*d*, 1H, H7′, *J* = 1.5 Hz), 3.78 (*s*, 3H, H11′), 3.74 (*s*, 6H, H10′ and H12′), 3.71 (*d*, 1H, H8′, *J* = 1.5 Hz), 3.66 (*s*, 3H, CH_3_O-9′), 2.29 (*s*, 3H, H3″); ^13^C NMR (CDCl_3_) δ (ppm) 198.4 (C2″) 171.9 (C9′), 153.2 (C3′ and C5′), 148.9 (C4), 147.2 (C5), 144.2 (C9), 138.2 (C1′), 137.0 (C7 and C4′), 131.1 (C2), 128.8 (C8), 126.4 (C1), 126.0 (C1″),109.7 (C3), 108.1 (C6), 104.3 (C2′ and C6′), 101.5 (C10), 60.8 (C11′), 56.0 (C10′ and C12′), 52.6 (CH_3_O-9′), 47.3 (C8′), 46.5 (C7′), 27.4 (C3″). EM: 466 *m*/*z*. IR ν_max_/cm^−1^ (film) 2921, 1731, 1667, 1651, 1578, 1505, 1458, 1224, 1126, 1005, 752.

*Compound* **6**. From **2** (200 mg, 0.47 mmol) and methyl (thiphenylphosphoranylidene)acetate (320 mg, 0.94 mmol) in 15 mL of toluene for 34 h. CC of the crude on silica gel (CH_2_Cl_2_/EtOAc 95:5) yielded **6** (190 mg, 84%). ^1^H NMR (CDCl_3_, 200 MHz) δ (ppm) 7.46 (*d*, 1H, H9, *J* = 16 Hz), 6.85 (*s*, 1H, H7), 6.75 (*s*, 1H, H6), 6.66 (*s*, 1H, H3), 6.25 (*s*, 2H, H2′ and H6′), 5.95 (*d*, 1H, H10a, *J* = 1.4 Hz), 5.94 (*d*, 1H, H10b, *J* = 1.4 Hz), 5.87 (*d*, 1H, H1″, *J* = 16 Hz), 4.52 (*d*, 1H, H7′, *J* = 1.6 Hz), 3.77 (*s*, 3H, H11′), 3.73 (*s*, 6H, H10′ and H12′), 3.69 (*d*, 1H, H8′, *J* = 1.6 Hz), 3.73 (*s*, 3H, CH_3_O-3″), 3.64 (*s*, 3H, CH_3_O-9′); ^13^C NMR (CDCl_3_) δ (ppm) 171.9 (C9′), 167.5 (C2″), 153.1 (C3′ and C5′), 148.7 (C4), 147.1 (C5), 145.4 (C9), 138.2 (C1′), 136.9 (C4′), 136.2 (C7), 131.2 (C2), 128.5 (C8), 126.2 (C1), 116.6 (C1″),109.6 (C3), 108.1 (C6), 104.3 (C2′ and C6′), 101.4 (C10), 60.7 (C11′), 56.0 (C10′ and C12′), 52.5 (CH_3_O-9′), 51.5 (C3″), 47.2 (C8′), 46.5 (C7′). HRMS calcd for C_26_H_26_O_9_ [M + H]^+^ 482.1577 u, found 482.1572 *m/z*; IR ν_max_/cm^−1^ (film) 2940, 1730, 1715, 1600, 1510, 1495, 1325, 1135, 1050, 1015. UV (EtOH) λ_max_ 213 (lg ε 4.4), 267 (lg ε 4.2), 368 (lg ε 4.3). [α]^22^_D_ −197° (c 0.97%).

*Compounds* **7** and **8**. To a solution of **6** (88 mg, 0.18 mmol) in dry ethyl ether (10 mL), a suspension of lithium aluminum hydride (14 mg, 0.37 mmol) in dry ether (5 mL) was added. It was stirred under argon at −15 °C for 3.5 h. The excess hydride was decomposed with wet EtOAc. The organic layer was dried over Na_2_SO_4_, filtered, and evaporated. CC of the crude afforded **7** (CH_2_Cl_2_/EtOAc 8:2, 14 mg, 14%) and **8** (EtOAc, 29 mg, 37%).

*Compound* **7**: ^1^H NMR (CDCl_3_, 200 MHz) δ (ppm) 7.41 (*d*, 1H, H9, *J* = 16 Hz), 6.77 (*s*, 1H, H7), 6.74 (*s*, 1H, H6), 6.71 (*s*, 1H, H3), 6.26 (*s*, 2H, H2′ and H6′), 5.98 (*s*, 2H, H10), 5.97 (*d*, 1H, H1″, *J* = 16 Hz), 4.30 (*s*, 1H, H7′), 3.78 (*s*, 3H, H11′), 3.75 (*s*, 3H, CH_3_O-3″), 3.74 (*s*, 6H, H10′ and H12′), 3.68 (*m*, 1H, H9a’), 3.39 (*t*, 1H, H9b’, *J* = 10 Hz), 3.01 (*dd*, 1H, H8′, *J* = 10 an*d* 4.9 Hz); ^13^C NMR (CDCl_3_) δ (ppm) 167.9 (C2″), 152.9 (C3′ and C5′), 148.7 (C4), 147.0 (C5), 145.4 (C9), 139.6 (C1′), -(C4′), 135.8 (C7), 131.5 (C2 and C8), 126.4 (C1), 115.9 (C1″), 110.4 (C3), 107.8 (C6), 104.2 (C2′ and C6′), 101.3 (C10), 62.7 (C9′), 60.7 (C11′), 55.9 (C10′ and C12′), 51.6 (C3″), 44.7 (C7′ and 8′). IR ν_max_/cm^−1^ (film) 3468, 2924, 1717, 1588, 1504, 1463, 1370, 1237, 1195, 1038, 934.

*Compound* **8**: ^1^H NMR (CDCl_3_, 200 MHz) δ (ppm) 6.67 (*s*, 1H, H3), 6.65 (*s*, 1H, H6), 6.40 (*s*, 1H, H7), 6.30 (*s*, 2H, H2′ and H6′), 6.28 (*d*, 1H, H9, *J* = 16 Hz), 5.93 (*d*, 1H, H10a, *J* = 1.5 Hz), 5.92 (*d*, 1H, H10b, *J* = 1.5 Hz), 5.89 (*m*, 1H, H1″), 4.23 (*m*, 1H, H2″), 3.79 (*d*, 1H, H7′, *J* = 1.6 Hz), 3.77 (*s*, 3H, H11′), 3.73 (*s*, 6H, H10′ and H12′), 3.69 (*dd*, 1H, H9a′, *J* = 11 and 4.6 Hz), 3.37 (*dd*, 1H, H9b’, *J* = 11 and 9.5 Hz), 3.01 (*ddd*, 1H, H8′, *J* = 9.5, 4.6 and 1.6 Hz); ^13^C NMR (CDCl_3_) δ (ppm) 152.9 (C3′ and C5′), 147.3 (C4), 146.7 (C5), 140.2 (C1′), 136.5 (C4′), 132.8 (C2), 132.1 (C7), -(C8), 129.9 (C1), 128.5 (C9), 127.2 (C1″), 110.2 (C3), 107.1 (C6), 104.5 (C2′ and C6′), 101.0 (C10), 63.6 (C2″), 63.1 (C9′), 60.7 (C11′), 56.0 (C10′ and C12′), 44.9 (C7′ and 8′). IR ν_max_/cm^−1^ (film) 3430, 2934, 1590, 1505, 1484, 1236, 1126, 1039, 934. UV (EtOH) λ_max_ 209 (lg ε 4.5), 295 (lg ε 3.7), 316 (lg ε 3.7). [α]^22^_D_ −37.1° (c 0.41%).

*Compound* **9**. A 3.0 M solution of methylmagnesium iodide in diethyl ether (0.17 mL) was added dropwise to a stirred solution of **2** (209 mg, 0.49 mmol) in dry THF at −78 °C under an argon atmosphere. Then, it was stirred and left to reach rt for 90 min and stirred for an additional 2 h. Then, a solution of saturated ammonium chloride was added and extracted with EtOAc. The combined organic layers were washed with brine, dried over Na_2_SO_4_, filtered, and evaporated. Insolubilization in EtOAc gave a mixture of epimers, **9** (23 mg, 11%). ^1^H NMR (CDCl_3_, 200 MHz) δ (ppm) 6.65/6.63 (*s*, 1H, H6), 6.34 (*m*, 1H, H7), 6.21 (*s*, 1H, H3), 6.53 (*bs*, 2H, H2′ and H6′), 6.28 (*d*, 1H, H9, *J* = 16 Hz), 5.92 (*d*, 1H, H10a, *J* = 1.3 Hz), 5.90 (*d*, 1H, H10b, *J* = 1.3 Hz), 5.20 (*m*, 1H, H9), 4.02/4.10 (*s*, 1H, H7′), 3.89 (*s*, 3H, H11′), 3.85 (*s*, 6H, H10′ and H12′), 3.80 (*m*, 1H, H8′), 1.63/1.54 (*d*, 3H, CH_3_-9, *J* = 6.0/6.8 Hz); ^13^C NMR (CDCl_3_) δ (ppm) 174.1 (C9′), 153.4 (C3′ and C5′), 147.5 (C4), 146.5 (C5), 136.9/136.1 (C1′), 136.5/136.6 (C4′), 130.9 (C2), 128.8 (C1), 126.8/127.1 (C8), 119.3/119.8 (C7), 109.3 (C3), 107.1 (C6), 103.6 (C2′ and C6′), 101.3 (C10), 76.8/77.3 (C9), 60.9 (C11′), 56.1 (C10′ and C12′), 46.9 (C7′), 45.7/43.4 (C8′), 18.8/21.5 (Me). IR ν_max_/cm^−1^ (film) 2924, 1774, 1592, 1506, 1483, 1234, 1132, 1034, 929. Purification by preparative thin-layer chromatography (Hexane/EtOAc) afforded compounds **9a** (3 mg), **9b** (3 mg), and **9c** (4 mg).

*Compound* **9a**: ^1^H NMR (CDCl_3_, 200 MHz) δ (ppm) 6.71 (*s*, 1H, H6), 6.64 (*s*, 1H, H3), 6.35 (*s*, 2H, H2′ and H6′), 5.95 (*d*, 1H, H10a, *J* = 1.3 Hz), 5.93 (*d*, 1H, H10b, *J* = 1.3 Hz), 5.04 (*qd*, 1H, *J* = 6.8 and 1.8, H9), 4.78 (*m*, 1H, H7′), 3.81 (*dd*, 1H, *J* = 23 and 4.2, H7a), 3.79 (*s*, 3H, H11′), 3.78 (*s*, 6H, H10′ and H12′), 3.59 (*dd*, 1H, *J* = 23 and 4.2, H7b), 1.50 (*d*, 3H, CH_3_-9, *J* = 6.8 Hz); ^13^C NMR (CDCl_3_) δ (ppm) 171.4 (C9′), 161.1 (C8), 153.2 (C3′ and C5′), 147.1 (C4), 146.9 (C5), 138.4 (C1′), 136.8 (C4′), 129.8 (C1), 127.7 (C2), 123.4 (C8′), 109.4 (C3), 107.8 (C6), 105.2 (C2′ and C6′), 101.2 (C10), 78.3 (C9), 60.7 (C11′), 56.0 (C10′ and C12′), 42.7 (C7′), 28.4(C7), 18.5 (Me). IR ν_max_/cm^−1^ (film) 2927, 1753, 1591, 1505, 1485, 1233, 1127, 1037, 934. EM: 410 *m/z*. UV (EtOH) λ_max_ 207 (lg ε 4.4), 259 (lg ε 4.2), 290 (lg ε 3.6), 310 (lg ε 3.6), 349 (lg ε 3.3). [α]^22^_D_ +26.9° (c 0.23%).

*Compound* **9b**: ^1^H NMR (CDCl_3_, 200 MHz) δ (ppm) 6.72 (*s*, 1H, H6), 6.62 (*s*, 1H, H3), 6.36 (*s*, 2H, H2′ and H6′), 5.95 (*d*, 1H, H10a, *J* = 1.5 Hz), 5.94 (*d*, 1H, H10b, *J* = 1.5 Hz), 5.07 (*q*, 1H, *J* = 6.8, H9), 4.79 (*t*, 1H, *J* = 4.2 Hz, H7′), 3.73 (*d*, 1H, *J* = 3.8 Hz, H7a), 3.78 (*s*, 3H, H11′), 3.77 (*s*, 6H, H10′ and H12′), 3.63 (*d*, 1H, *J* = 4.1, H7b), 1.51 (*d*, 3H, CH_3_-9, *J* = 6.8 Hz); ^13^C NMR (CDCl_3_) δ (ppm) 171.3 (C9′), 160.9 (C8), 153.1 (C3′ and C5′), 147.1 (C4), 146.9 (C5), 138.3 (C1′), 136.9 (C4′), 129.6 (C1), 127.6 (C2), 123.8 (C8′), 109.4 (C3), 107.8 (C6), 105.5 (C2′ and C6′), 101.2 (C10), 78.1 (C9), 60.7 (C11′), 56.1 (C10′ and C12′), 42.7 (C7′), 28.3 (C7), 18.1 (Me). IR ν_max_/cm^−1^ (film) 2980, 1757, 1584, 1505, 1485, 1237, 1126, 1037, 938. EM: 410 *m/z*. UV (EtOH) λ_max_ 210 (lg ε 4.5), 258 (lg ε 4.5), 310 (lg ε 3.8), 350 (lg ε 3.6). [α]^22^_D_ +23.0° (c 0.58%).

*Compound* **9c**: ^1^H NMR (CDCl_3_, 200 MHz) δ (ppm) 7.65 (*s*, 1H, H7), 7.21 (*s*, 1H, H6), 7.10 (*m*, 1H, H3), 6.56 (*d*, 1H, *J* = 2.8 Hz, H2′), 6.54 (*d*, 1H, *J* = 2.8 Hz, H6′), 6.09 (*s*, 2H, H10), 5.62 (*q*, 1H, *J* = 6.6 Hz, H9), 3.97 (*s*, 3H, H11′), 3.85 (*s*, 3H, H10′), 3.84 (*s*, 3H, H12′), 1.73 (*d*, 3H, CH_3_-9, *J* = 6.6 Hz); ^13^C NMR (CDCl_3_) δ (ppm) 169.0 (C9′), 152.9 (C3′ and C5′), 150.0 (C4), 148.7 (C5), 144.8 (C8), 140.3 (C7′), 137.7 (C4′), 134.6 (C1′), 130.4, 130.3 (C2 and C1), 118.8 (C8′), 118.7 (C7), 107.3 (C3), 107.1 (C6), 103.8 (C2′ and C6′), 101.8 (C10), 75.8 (C9), 61.8 (C11′), 56.1 (C10′ and C12′), 21.1 (Me). EM: 408 *m/z*.

*Compound* **10**. A stirred suspension of zinc (62 mg, 0.52 mmol) in dry THF was refluxed in a two-necked flask under an inert atmosphere. Then, a solution of ethyl bromodifluoroacetate (0.12 mL, 0.94 mmol) and aldehyde **2** (200 mg, 0.47 mmol) in a 1:1 mixture of dry CH_2_Cl_2_:THF was added through a septum. The reaction mixture was refluxed for 24 h, and the solvent was removed under vacuum. The residue was dissolved in chloroform, washed with a 5% tetrasodium ethylenediaminetretraacetate (EDTA) solution (pH = 10) and brine, dried over Na_2_SO_4_, filtered, and evaporated. Purification by silica gel CC (CH_2_Cl_2_/EtOAc 92:8) yielded compound **10** (50 mg, 21%). ^1^H NMR (CDCl_3_, 200 MHz) δ (ppm) 6.67 (*s*, 1H, H6), 6.60 (*d*, 1H *J* = 3.0 Hz, H7), 6.21 (*s*, 1H, H3), 6.50 (*bs*, 2H, H2′ and H6′), 5.93 (*d*, 1H, H10a, *J* = 1.3 Hz), 5.91 (*d*, 1H, H10b, *J* = 1.3 Hz), 5.47 (*dd*, 1H, *J* = 16 and 7.2 Hz, H9), 4.41 (*q*, 2H, *J* = 7.1 Hz, O-CH_2_-CH_3_), 4.04 (*d*, 1H, *J* = 16 Hz, H7′), 3.89 (*s*, 3H, H11′), 3.84 (*s*, 6H, H10′ and H12′), 3.76 (*m*, 1H, H8′), 1.39 (*t*, 3H, *J* = 7.1 Hz, O-CH_2_-CH_3_); ^13^C NMR (CDCl_3_) δ (ppm) 172.6 (C9′), 161.9 (*t*, C2″), 153.6 (C3′ and C5′), 148.2 (C4), 146.7 (C5), 137.4 (C4′), 135.5 (C1′), 131.5 (C2), 126.2 (C1 and C8), 125.9 (C7), 111.2 (*t*, C1″), 109.7 (C2′ and C6′), 109.3 (C3), 107.6 (C6), 101.4 (C10), 77.2 (*t*, C9), 63.7 (O-CH_2_-CH_3_), 60.8 (C11′), 56.1 (C10′ and C12′), 47.0 (C7′), 43.9 (C8′), 13.9 (O-CH_2_-CH_3_).); HRMS calcd for C_26_H_24_O_9_F [M + H]^+^ 518.1388 u, found 518.1397 *m/z*; IR ν_max_/cm^−1^ (film) 2938, 1789, 1752, 1592, 1509, 1485, 1259, 1128, 1033, 928. UV (EtOH) λ_max_ 210 (lg ε 4.5), 224 (lg ε 4.4), 301 (lg ε 3.9), 317 (lg ε 3.9). [α]^22^_D_ −37.3° (c 0.51%).

*The general procedure for the synthesis of benzimidazoles* **11**–**15**. To a solution of **2** (0.10–0.16 mmol) in EtOH (5 mL), *p*-benzoquinone (*p*-BQ) (0.11–0.16 mmol) and the corresponding phenylenediamine (0.10–0.16 mmol) were added. The mixture was stirred at reflux for a specified time and then concentrated under vacuum. The reaction product was purified by CC on silica gel (CH_2_Cl_2_/EtOAc 8:2) to yield the corresponding benzimidazole.

*Benzimidazole* **11**. From **2** (48 mg, 0.11 mmol) in EtOH (5 mL), *p*-BQ (15 mg, 0.14 mmol), and 1,2-phenylenediamine (16 mg, 0.14 mmol) following the general procedure for 4.5 h. CC of the crude on silica gel (CH_2_Cl_2_/EtOAc 8:2) afforded **11** (33 mg, 57%). ^1^H NMR (CDCl_3_, 400 MHz) δ (ppm) 7.38 (*bs*, 1H, H7), 7.38 (*m*, 2H, H3″ and H6″), 7.15 (*m*, 2H, H4″ and H5″), 6.70 (*s*, 1H, H3), 6.61 (*bs*, 1H, H6), 6.35 (*s*, 2H, H2′ and H6′), 5.95 (*d*, 1H, H10a, *J* = 1.3 Hz), 5.93 (*d*, 1H, H10b, *J* = 1.3 Hz), 4.65 (*d*, 1H, H7′, *J* = 1.6 Hz), 4.50 (*bs*, 1H, H8′), 3.74 (*s*, 3H, H11′), 3.69 (*s*, 6H, H10′ and H12′), 3.65 (*s*, 3H, CH_3_O-9′); ^13^C NMR (CDCl_3_, 200 MHz) δ (ppm) 173.2 (C9′), 153.0 (C3′ and C5′), 151.5 (C9), 148.3 (C4), 147.0 (C5), 143.0 (C1″), 138.0 (C1′), 136.7 (C4′), 134.2 (C2″), 130.1 (C2), 129.2 (C7), 126.0 (C1), 122.7 (C4″ and C5″), 122.0 (C8), 119.0 (C6″), 110.5 (C3″), 109.6 (C3), 107.9 (C6), 104.8 (C2′ and C6′), 101.3 (C10), 60.7 (C11′), 56.0 (C10′ and C12′), 52.7 (CH_3_O-9′), 47.7 (C8′), 46.1 (C7′); HRMS calcd for C_29_H_27_N_2_O_7_ [M+H]^+^ 515.1818 u, found 515.1829 *m/z*; IR ν_max_/cm^−1^ (film) 2953, 2924, 1731, 1590, 1504, 1484, 1459, 1278, 1234, 1126, 1037, 744.

*Benzimidazole* **12**. To a solution of **2** (49 mg, 0.12 mmol) in abs EtOH, aq HCl 2 N (0.5 mL) and 1,2-phenylenediamine (31 mg, 0.28 mmol) were added and continuously stirred at 135 °C under an argon atmosphere for 24 h. The reaction was diluted with water and extracted with EtOAc. The combined organic layers were washed with brine, dried over Na_2_SO_4_, and filtered, and the solvent was evaporated. CC of the residue (CH_2_Cl_2_/EtOAc 8:2) yielded aldehyde **2** (41%) and compound **12** (28%). ^1^H NMR (CDCl_3_, 400 MHz) δ (ppm) 7.52 (*bs*, 1H, H7), 7.48 (*m*, 2H, H3″ and H6″), 7.16 (*m*, 2H, H4″ and H5″), 6.76 (*s*, 1H, H6), 6.70 (*bs*, 1H, H3), 6.36 (*s*, 2H, H2′ and H6′), 5.98 (*s*, 1H, H10a), 5.97 (*s*, 1H, H10b), 4.64 (*bs*, 1H, H7′), 4.39 (*bs*, 1H, H8′), 4.13 (*dq*, 2H, *J* = 7.1 and 2.3, CH_3_-CH_2_-O-9′), 3.73 (*s*, 3H, H11′), 3.71 (*s*, 6H, H10′ and H12′), 1.14 (*t*, 3H, *J* = 7.1, CH_3_-CH_2_-O-9′); ^13^C NMR (CDCl_3_, 200 MHz) δ (ppm) 172.4 (C9′), 153.1 (C3′ and C5′), 151.2 (C9), 148.6 (C4), 147.0 (C5), 137.7 (C1′), 136.9 (C4′), 130.6 (C2), 123.1 (C7), 125.8 (C1), 123.1 (C4″ and C5″), 109.6 (C3), 108.2 (C6), 104.7 (C2′ and C6′), 101.4 (C10), 61.7 (CH_3_-CH_2_-O-9′), 60.7 (C11′), 56.1 (C10′ and C12′), 48.2 (C8′), 46.0 (C7′), 14.0 (CH_3_-CH_2_-O-9′); HRMS calcd for C_30_H_28_N_2_O_7_ [M+H]^+^ 529.1974 u, found 529.1921 *m/z*; IR ν_max_/cm^−1^ (film) 2954, 2923, 1728, 1589, 1504, 1485, 1460, 1235, 1126, 1036, 746. [α]^22^_D_ −72.4° (c 0.17%)

*Benzimidazole* **13**. From **2** (70 mg, 0.16 mmol) in EtOH (5 mL), *p*-BQ (18 mg, 0.16 mmol), and 4,5-dimethyl-1,2-phenylenediamine (27 mg, 0.20 mmol) following the general procedure for 4.5 h. CC of the crude on silica gel (CH_2_Cl_2_/EtOAc 9:1) afforded **13** (14 mg, 16%). ^1^H NMR (CDCl_3_, 400 MHz) δ (ppm) 7.41 (*m*, 1H, H6″), 7.31 (*s*, 1H, H7), 7.04 (*m*, 1H, H3″), 6.70 (*s*, 1H, H3), 6.65 (*s*, 1H, H6), 6.36 (*s*, 2H, H2′ and H6′), 5.96 (*d*, 1H, H10a, *J* = 1.4 Hz), 5.95 (*d*, 1H, H10b, *J* = 1.4 Hz), 4.63 (*d*, 1H, H7′, *J* = 2.1 Hz), 4.48 (*d*, 1H, H8′, *J* = 2.1 Hz), 3.73 (*s*, 1H, H11′), 3.69 (*s*, 2H, H10′ and H12′), 3.67 (*s*, 3H, CH_3_O-9′), 2.30 (*s*, 6H, CH_3_-4″ and CH_3_-5″); ^13^C NMR (CDCl_3_, 200 MHz) δ (ppm) 173.3 (C9′), 153.0 (C3′ and C5′), 150.5 (C9), 148.2 (C4), 146.9 (C5), 142.1 (C1″), 137.9 (C1′), 136.8 (C4′), 132.3 (C2″ and C5″), 131.1 (C4″), 130.3 (C2), 128.3 (C7), 126.1 (C1), 122.2 (C8), 119.2 (C6″), 110.7 (C3″), 109.6 (C3), 108.0 (C6), 104.7 (C2′ and C6′), 101.3 (C10), 60.7 (C11′), 56.1 (C10′ and C12′), 52.7 (CH_3_O-9′), 47.7 (C8′), 46.2 (C7′), 20.3 (CH_3_-4″ and CH_3_-5″); HRMS calcd for C_31_H_31_N_2_O_7_ [M + H]^+^ 543.2131 u, found 543.2147 *m/z*; IR ν_max_/cm^−1^ (film) 3310, 2931, 2873, 2852, 1695, 1465, 1454, 1236, 1128, 1036, 890, 737.

*Compound* **13** was also obtained from the following procedure: A mixture of aldehyde **2** (52 mg, 0.12 mmol) and 4,5-dimethyl-1,2-phenylenediamine (18 mg, 0.13 mmol) in acetonitrile (2 mL) was stirred at 90 °C for 1 h. FeCl_3_·6H_2_O (1 mg, 0.004 mmol) was then added, and the mixture was stirred with heating at 90 °C, under continuous O_2_ bubbling, for 7 h. The solvent was evaporated, and the residue was purified by silica gel CC (CH_2_Cl_2_/EtOAc 9:1) to give **13** (37 mg, 56%).

*Benzimidazole* **14**. From **2** (46 mg, 0.11 mmol) in EtOH (4 mL), *p*-BQ (14 mg, 0.13 mmol), and 4-methoxy-1,2-phenylenediamine (23 mg, 0.11 mmol) following the general procedure for 6.5 h. CC of the crude on silica gel (CH_2_Cl_2_/EtOAc 7:3) afforded **14** (38 mg, 65%). ^1^H NMR (CDCl_3_, 200 MHz) δ (ppm) 7.37 (*s*, 1H, H7), 7.35 (*m*, 1H, H5″), 6.83 (*d*, 1H, H6″, *J* = 2.5 Hz), 6.78 (*d*, 1H, H3″, *J* = 2.5 Hz), 6.70 (*s*, 1H, H3), 6.66 (*s*, 1H, H6), 6.34 (*s*, 2H, H2′ and H6′), 5.97 (*d*, 1H, H10a, *J* = 1.5 Hz), 5.96 (*d*, 1H, H10b, *J* = 1.5 Hz), 4.65 (*d*, 1H, H7′, *J* = 2.0 Hz), 4.46 (*d*, 1H, H8′, *J* = 2.0 Hz), 3.81 (*s*, 3H, CH_3_O-4″), 3.73 (*s*, 1H, H11′), 3.71 (*s*, 2H, H10′ and H12′), 3.69 (*s*, 3H, CH_3_O-9′); ^13^C NMR (CDCl_3_, 200 MHz) δ (ppm) 173.4 (C9′), 156.7 (C4″), 153.0 (C3′ and C5′), 150.8 (C9), 148.4 (C4), 147.1 (C5), 137.9 (C1′), 136.9 (C4′), 130.2 (C2), 129.0 (C7), 126.0 (C1), 121.4 (C8), 112.6 (C3″ and C6″), 109.6 (C3), 108.1 (C6), 104.8 (C2′ and C6′), 101.4 (C10), 60.7 (C11′), 56.1 (C10′ and C12′), 55.6 (CH_3_O-4″), 52.8 (CH_3_O-9′), 47.7 (C8′), 46.1 (C7′); HRMS calcd for C_30_H_29_N_2_O_8_ [M + H]^+^ 545.1924 u, found 545.1974 *m/z*; IR ν_max_/cm^−1^ (film) 2926, 1737, 1732, 1590, 1505, 1485, 1463, 1456, 1417, 1373, 1327, 1274, 1237, 1126, 1036, 825, 730.

*Benzimidazole* **15**. From **2** (45 mg, 0.11 mmol) in EtOH (5 mL), *p*-BQ (14 mg, 0.13 mmol), and 4,5-dichloro-1,2-phenylenediamine (20 mg, 0.11 mmol) following the general procedure for 6 h. CC of the crude on silica gel (CH_2_Cl_2_/EtOAc 85:15) afforded **15** (62 mg, 99%). ^1^H NMR (CDCl_3_, 400 MHz) δ (ppm) 7.57 (*bs*, 1H, H6″), 7.15 (*s*, 1H, H7), 7.01 (*bs*, 1H, H3″), 6.69 (*s*, 1H, H3), 6.33 (*s*, 2H, H2′ and H6′), 6.24 (*s*, 1H, H6), 5.97 (*d*, 1H, H10a, *J* = 1.3 Hz), 5.94 (*d*, 1H, H10b, *J* = 1.3 Hz), 4.68 (*d*, 1H, H7′, *J* = 2.8 Hz), 4.66 (*d*, 1H, H8′, *J* = 2.8 Hz), 3.82 (*s*, 3H, CH_3_O-9′), 3.74 (*s*, 1H, H11′), 3.71 (*s*, 2H, H10′ and H12′); ^13^C NMR (CDCl_3_, 200 MHz) δ (ppm) 174.7 (C9′), 153.1 (C3′ and C5′), 152.9 (C9), 148.6 (C4), 147.0 (C5), 142.6 (C1″), 137.7 (C1′), 137.1 (C4′), 133.0 (C2″), 130.2 (C7), 130.1 (C2), 126.8 (C5″), 126.0 (C4″), 125.6 (C1), 121.3 (C8), 119.9 (C3″), 111.5 (C6″), 109.6 (C3), 108.2 (C6), 105.1 (C2′ and C6′), 101.5 (C10), 60.7 (C11′), 56.2 (C10′ and C12′), 53.2 (CH_3_O-9′), 47.6 (C8′), 46.3 (C7′); HRM*S* calcd for C_29_H_25_N_2_O_7_ [M + H]^+^ 583.0960 u, found 583.1016 *m/z*; Anal. calcd for C_29_H_24_N_2_O_7_Cl_2_: C, 59.70; H, 4.15; N, 4.80; found: C, 57.53; H, 4.25; N, 4.83; IR ν_max_/cm^−1^ (film) 3290, 2952, 2920, 2850, 1731, 1592, 1504, 1485, 1462, 1454, 1417, 1374, 1295, 1235, 1126, 1098, 1038, 1005, 828.

*The general procedure for the synthesis of benzoxazoles* **16**–**18**. A solution of compound **2** (0.12–0.14 mmol) and the corresponding 2-aminophenol (0.12–0.20 mmol) in dry EtOH (2 mL) was stirred at reflux for the specified time. The solvent was evaporated, and the residue was redissolved in glacial acetic acid (1 mL). Pb(AcO)_4_ (0.12–0.34 mmol) was added to the solution, and it was then stirred at room temperature for a time. After dilution with water, the mixture was extracted with EtOAc, and the combined organic layers were washed with brine, dried over Na_2_SO_4_, filtered, and evaporated. Purification by silica gel column chromatography (Hexane/EtOAc) of the crude provided the corresponding benzoxazoles.

*Benzoxazole* **16**. From **2** (50 mg, 0.12 mmol) and 2-aminophenol (13 mg, 0.12 mmol) in dry EtOH (2 mL) for 46 h. Then, in glacial acetic acid (1 mL) with Pb(AcO)_4_ (55 mg, 0.12 mmol) for 24 h. CC (Hexane/ EtOAc 7:3) of the crude provided **16** (36 mg, 59%). ^1^H NMR (CDCl_3_, 200 MHz) δ (ppm) 7.73 (*s*, 1H, H7), 7.67 (*m*, 1H, H3″), 7.49 (*m*, 1H, H6″), 7.31 (*m*, 1H, H4″), 7.29 (*m*, 1H, H5″), 6.90 (*s*, 1H, H6), 6.72 (*s*, 1H, H3), 6.30 (*s*, 2H, H2′ and H6′), 6.01 (*d*, 1H, H10a, *J* = 1.3 Hz), 5.99 (*d*, 1H, H10b, *J* = 1.3 Hz), 4.72 (*d*, 1H, H7′, *J* = 1.8 Hz), 4.40 (*d*, 1H, H8′, *J* = 1.8 Hz), 3.75 (*s*, 1H, H11′), 3.72 (*s*, 2H, H10′ and H12′), 3.65 (*s*, 3H, CH_3_O-9′); ^13^C NMR (CDCl_3_, 400 MHz) δ (ppm) 172.2 (C9′), 163.0 (C9), 153.1 (C3′ and C5′), 150.5 (C1″), 149.0 (C4), 147.2 (C5), 142.1 (C2″), 137.6 (C1′), 137.0 (C4′), 132.7 (C7), 131.0 (C2), 126.0 (C1), 125.1 (C5″), 124.4 (C4″), 119.8 (C3″), 119.4 (C8), 110.2 (C6″), 110.0 (C3), 108.4 (C6), 104.8 (C2′ and C6′), 101.5 (C10), 60.7 (C11′), 56.0 (C10′ and C12′), 52.7 (CH_3_O-9′), 47.4 (C8′), 46.2 (C7′); HRMS calcd for C_29_H_26_NO_8_ [M + H)]^+^ 516.1658 u, found 516.1691 *m/z*; IR ν_max_/cm^−1^ (film) 2952, 2937, 1732, 1590, 1504, 1485, 1455, 1418, 1371, 1328, 1242, 1128, 1037, 1007, 933, 808, 765, 747.

*Benzoxazole* **17**. From **2** (58 mg, 0.14 mmol) and 2-amino-4-methylphenol (17 mg, 0.14 mmol) in dry EtOH (2 mL) for 23 h. Then, in glacial acetic acid (1 mL) with Pb(AcO)_4_ (159 mg, 0.34 mmol) for 44 h. CC (Hexane/EtOAc 8:2) of the crude provided **17** (41 mg, 57%). ^1^H NMR (CDCl_3_, 200 MHz) δ (ppm) 7.70 (*s*, 1H, H7), 7.44 (*bs*, 1H, H3″), 7.35 (*d*, 1H, H6″, *J* = 8.4 Hz), 7.10 (*dd*, 1H, H5″, *J*_1_ = 8.4 Hz, *J*_2_ = 1.5 Hz), 6.88 (*s*, 1H, H6), 6.71 (*s*, 1H, H3), 6.30 (*s*, 2H, H2′ and H6′), 5.99 (*d*, 1H, H10a, *J* = 1.5 Hz), 5.97 (*d*, 1H, H10b, *J* = 1.5 Hz), 4.71 (*d*, 1H, H7′, *J* = 2.0 Hz), 4.39 (*d*, 1H, H8′, *J* = 2.0 Hz), 3.74 (*s*, 1H, H11′), 3.71 (*s*, 2H, H10′ and H12′), 3.63 (*s*, 3H, CH_3_O-9′), 2.43 (*s*, 3H, CH_3_-4″); ^13^C NMR (CDCl_3_, 200 MHz) δ (ppm) 172.3 (C9′), 163.1 (C9), 153.1 (C3′ and C5′), 149.0 (C4), 148.7 (C1″), 147.3 (C5), 142.2 (C2″), 137.7 (C1′), 136.9 (C4′), 134.3 (C4″), 132.5 (C7), 131.0 (C2), 126.2 (C5″), 126.0 (C1), 119.7 (C3″), 119.5 (C8), 110.0 (C3), 109.6 (C6″), 108.4 (C6), 104.7 (C2′ and C6′), 101.5 (C10), 60.7 (C11′), 56.0 (C10′ and C12′), 52.7 (CH_3_O-9′), 47.4 (C8′), 46.2 (C7′), 21.5 (CH_3_-4″); HRMS calcd for C_30_H_28_NO_8_ [M + H]^+^ 530.1815 u, found 530.1863 *m/z*; IR ν_max_/cm^−1^ (film) 2952, 2937, 1732, 1591, 1504, 1485, 1462, 1418, 1371, 1329, 1239, 1127, 1037, 1010, 933, 819, 735.

*Benzoxazole* **18**. From **2** (54 mg, 0.13 mmol) and 2-amino-4-chlorophenol (29 mg, 0.20 mmol) in dry EtOH (2 mL) for 47 h. Then, in glacial acetic acid (1 mL) with Pb(AcO)_4_ (60 mg, 0.13 mmol) for 26 h. CC of the crude on silica gel (CH_2_Cl_2_/EtOAc 98:2) provided **18** (27 mg, 39%). ^1^H NMR (CDCl_3_, 200 MHz) δ (ppm) 7.73 (*s*, 1H, H7), 7.62 (*d*, 1H, H3″, *J* = 2.2 Hz), 7.39 (*d*, 1H, H6″, *J* = 8.4 Hz), 7.26 (*dd*, 1H, H5″, *J*_1_ = 8.4 Hz, *J*_2_ = 2.2 Hz), 6.89 (*s*, 1H, H6), 6.71 (*s*, 1H, H3), 6.29 (*s*, 2H, H2′ and H6′), 6.01 (*d*, 1H, H10a, *J* = 1.3 Hz), 5.99 (*d*, 1H, H10b, *J* = 1.3 Hz), 4.72 (*d*, 1H, H7′, *J* = 2.0 Hz), 4.36 (*d*, 1H, H8′, *J* = 2.0 Hz), 3.75 (*s*, 1H, H11′), 3.72 (*s*, 2H, H10′ and H12′), 3.65 (*s*, 3H, CH_3_O-9′); ^13^C NMR (CDCl_3_, 200 MHz) δ (ppm) 172.1 (C9′), 164.3 (C9), 153.1 (C3′ and C5′), 149.2 (C1″), 149.0 (C4), 147.3 (C5), 143.2 (C2″), 137.5 (C1′), 137.0 (C4′), 133.5 (C7), 131.1 (C4″), 129.8 (C2), 125.8 (C1), 125.3 (C5″), 119.7 (C3″), 118.9 (C8), 110.9 (C6″), 110.0 (C3), 108.5 (C6), 104.6 (C2′ and C6′), 101.6 (C10), 60.7 (C11′), 56.0 (C10′ and C12′), 52.7 (CH_3_O-9′), 47.4 (C8′), 46.2 (C7′); Anal. calcd for C_29_H_24_NO_8_Cl: C, 63.33; H, 4.40; N, 2.55; found: C, 63.37; H, 4.69; N, 2.54; IR ν_max_/cm^−1^ (film) 2934, 2838, 1732, 1589, 1537, 1505, 1485, 1455, 1255, 1238, 1127, 1037, 1010, 934, 816, 735, 703.

*Synthesis of benzothiazoles* **19** and **20**. To a solution of aldehyde **2** (50 mg, 0.12 mmol), 2-aminothiophenol (15 μL, 0.14 mmol) or 2-amino-4-(trifluoromethyl)thiophenol (28 mg, 0.12 mmol) and *p*-toluensulfonic acid (2 mg, 0.01 mmol) in dry toluene (5 mL) MgSO_4_ (34 mg, 0.28 mmol) were added, and the mixture was stirred at reflux for 24 h. The mixture was then filtered, and the solvent was evaporated under vacuum. The residue was redissolved in EtOAc and neutralized with aq saturated NaHCO_3_ and washed with brine, dried over Na_2_SO_4_, filtered, and evaporated off. CC of the crude on silica gel (CH_2_Cl_2_/EtOAc 96:4 or 98:2) afforded **19** (32 mg, 52%) or **20** (55 mg, 77%).

*Compound* **19**. ^1^H NMR (CDCl_3_, 200 MHz) δ (ppm) 7.92 (*m*, 1H, H3″), 7.82 (*m*, 1H, H6″), 7.42 (*m*, 1H, H4″), 7.40 (*s*, 1H, H7), 7.34 (*m*, 1H, H5″), 6.86 (*s*, 1H, H6), 6.73 (*s*, 1H, H3), 6.38 (*s*, 2H, H2′ and H6′), 5.99 (*s*, 1H, H10a), 5.98 (*s*, 1H, H10b), 4.67 (*d*, 1H, H7′, *J* = 2.4 Hz), 4.61 (*d*, 1H, H8′, *J* = 2.4 Hz), 3.75 (*s*, 1H, H11′), 3.71 (*s*, 2H, H10′ and H12′), 3.62 (*s*, 3H, CH_3_O-9′); ^13^C NMR (CDCl_3_, 200 MHz) δ (ppm) 172.5 (C9′), 167.8 (C9), 153.7 (C1″), 153.0 (C3′ and C5′), 148.7 (C4), 147.1 (C5), 137.8 (C1′), 136.8 (C4′), 134.4 (C2″), 131.8 (C7), 131.3 (C2), 127.4 (C8), 126.1 (C1), 126.0 (C4″), 125.2 (C5″), 123.0 (C3″), 121.3 (C6″), 109.8 (C3), 108.2 (C6), 104.6 (C2′ and C6′), 101.4 (C10), 60.7 (C11′), 56.0 (C10′ and C12′), 52.5 (CH_3_O-9′), 48.3 (C8′), 46.5 (C7′); HRMS calcd for C_29_H_26_NO_7_S [M + H]^+^ 532.1430 u, found 532.1381 *m/z*; Anal. calcd for C_29_H_25_NO_7_S: C, 65.52; H, 4.74; N, 2.63; *S*, 6.03; found: C, 65.05; H, 4.96; N, 2.54; *S*, 5.94; IR ν_max_/cm^−1^ (film) 2952, 2927, 1732, 1588, 1504, 1486, 1456, 1238, 1126, 1036, 1009, 731, 686.

*Compound* **20**. ^1^H NMR (CDCl_3_, 400 MHz) δ (ppm) 8.19 (*bs*, 1H, H3″), 7.92 (*d*, 1H, H6″, *J* = 8.4 Hz), 7.56 (*dd*, 1H, H5″, *J*_1_ = 8.4 Hz, *J*_2_ = 1.5 Hz), 7.43 (*s*, 1H, H7), 6.87 (*s*, 1H, H6), 6.73 (*s*, 1H, H3), 6.36 (*s*, 2H, H2′ and H6′), 6.01 (*d*, 1H, H10a, *J* = 1.3 Hz), 5.99 (*d*, 1H, H10b, *J* = 1.3 Hz), 4.69 (*d*, 1H, H7′, *J* = 2.2 Hz), 4.58 (*d*, 1H, H8′, *J* = 2.2 Hz), 3.75 (*s*, 1H, H11′), 3.72 (*s*, 2H, H10′ and H12′), 3.64 (*s*, 3H, CH_3_O-9′); ^13^C NMR (CDCl_3_, 200 MHz) δ (ppm) 172.3 (C9′), 169.8 (C9), 153.3 (C4″), 153.1 (C3′ and C5′), 149.0 (C4), 147.2 (C5), 137.8 (C1″), 137.7 (C1′), 137.0 (C4′), 133.1 (C7), 131.5 (C2), 129.1 (C2″), 127.0 (C8), 125.8 (C1), 125.5 (CF_3_-4″), 121.8 (C6″), 121.5 (C5″), 120.1 (C3″), 109.9 (C3), 108.3 (C6), 104.6 (C2′ and C6′), 101.5 (C10), 60.7 (C11′), 56.1 (C10′ and C12′), 52.6 (CH_3_O-9′), 48.4 (C8′), 46.5 (C7′); HRMS calcd for C_30_H_25_NO_7_SF_3_ [M + H]^+^ 600.1304 u, found 600.1337 *m/z*; IR ν_max_/cm^−1^ (film) 2953, 2839, 1732, 1589, 1505, 1487, 1463, 1418, 1330, 1232, 1126, 1037, 1008, 933, 736.

*Compound* **21**. To a solution of compound **2** (50 mg, 0.12 mmol) in nitrobenzene (5 mL), 2,3-diaminopyridine (26 mg, 0.24 mmol) was added. The mixture was stirred at 145 °C under a N_2_ atmosphere for 3 d. Purification by silica gel CC (CH_2_Cl_2_/MeOH 97:3) provided compound **21** (48 mg, 79%). ^1^H NMR (CDCl_3_, 200 MHz) δ (ppm) 8.35 (*d*, 1H, H6″, *J* = 5.1 Hz), 8.03 (*m*, 1H, H4″), 7.65 (*s*, 1H, H7), 7.26 (*m*, 1H, H5″), 6.84 (*s*, 1H, H6), 6.75 (*s*, 1H, H3), 6.36 (*s*, 2H, H2′ and H6′), 6.02 (*s*, 1H, H10a), 6.00 (*s*, 1H, H10b), 4.75 (*d*, 1H, H7′, *J* = 1.9 Hz), 4.61 (*d*, 1H, H8′, *J* = 1.9 Hz), 3.72 (*s*, 1H, H11′), 3.67 (*s*, 2H, H10′ and H12′), 3.63 (*s*, 3H, CH_3_O-9′); ^13^C NMR (CDCl_3_, 200 MHz) δ (ppm) 172.6 (C9′), 153.8 (C9), 153.0 (C3′ and C5′), 149.5 (C2″), 148.7 (C4), 147.2 (C5), 142.4 (C6″), 137.7 (C1′), 136.7 (C4′), 130.8 (C7), 130.6 (C2), 126.9 (C3″), 126.0 (C1), 122.4 (C8), 118.2 (C5″), 109.9 (C3), 107.9 (C6), 104.6 (C2′and C6′), 101.5 (C10), 60.6 (C11′), 55.9 (C10′ and C12′), 52.6 (CH_3_O-9′), 47.5 (C8′), 46.2 (C7′); HRMS calcd for C_28_H_26_N_3_O_7_ [M + H]^+^ 516.1770 u, found 516.1799 *m/z*; IR ν_max_/cm^−1^ (film) 2953, 2921, 2850, 1738, 1732, 1589, 1504, 1485, 1463, 1456, 1414, 1279, 1259, 1235, 1125, 1037, 1007, 935, 900, 785.

*Compound* **22**. To a solution of 4,5-diaminopyrimidine (10 mg, 0.09 mmol) in *N*,*N*-dimethylacetamide (2 mL), Na_2_S_2_O_5_ (23 mg, 0.12 mmol) was first added, and then aldehyde **2** (38 mg, 0.09 mmol). The mixture was stirred at 100 °C for 97 h. The solvent was evaporated under reduced pressure, and the crude product was purified by CC on silica gel (EtOAc), providing **22** (26 mg, 56%). ^1^H NMR (CDCl_3_, 200 MHz) δ (ppm) 9.04 (*s*, 1H, H6″), 8.95 (*s*, 1H, H2″), 7.58 (*s*, 1H, H7), 6.75 (*s*, 1H, H6), 6.73 (*s*, 1H, H3), 6.31 (*s*, 2H, H2′ and H6′), 6.01 (*s*, 1H, H10a), 6.00 (*s*, 1H, H10b), 4.74 (*d*, 1H, H7′, *J* = 1.8 Hz), 4.55 (*d*, 1H, H8′, *J* = 1.8 Hz), 3.73 (*s*, 1H, H11′), 3.70 (*s*, 2H, H10′ and H12′), 3.69 (*s*, 3H, CH_3_O-9′); ^13^C NMR (CDCl_3_, 200 MHz) δ (ppm) 172.5 (C9′), 154.4 (C9), 153.2 (C4″), 153.1 (C3′ and C5′), 151.5 (C2″), 149.3 (C4), 147.3 (C5), 147.2 (C6″), 137.5 (C1′), 137.1 (C4′), 135.2 (C5″), 132.3 (C7), 131.0 (C2), 125.4 (C1), 121.4 (C8), 110.0 (C3), 108.2 (C6), 104.8 (C2′ and C6′), 101.6 (C10), 60.7 (C11′), 56.1 (C10′ and C12′), 52.8 (CH_3_O-9′), 47.6 (C8′), 46.1 (C7′); HRMS calcd for C_27_H_25_N_4_O_7_ [M + H]^+^ 517.1723, found 517.1669; Anal. calcd for C_27_H_24_N_4_O_7_: C, 62.79; H, 4.68; N, 10.85; found: C, 62.18; H, 5.02; N, 10.34; IR ν_max_/cm^−1^ (film) 2930, 2851, 1732, 1606, 1590, 1505, 1485, 1463, 1417, 1377, 1260, 1238, 1126, 1037, 1007, 910, 730.

Compound **22** was also obtained through the following procedure: To a solution of compound **2** (52 mg, 0.12 mmol) in dry EtOH (2 mL), MgSO_4_ (30 mg, 0.25 mmol) and 4,5-diaminopyrimidine (17 mg, 0.15 mmol) were added, and the mixture was stirred at reflux for 98 h. The solvent was then evaporated, and the residue was redissolved in glacial acetic acid (2 mL). The reaction mixture was stirred at reflux for 48 h. After dilution with water, the mixture was extracted with EtOAc, and the combined organic layers were washed with brine, dried over Na_2_SO_4_, filtered, and evaporated. Purification by silica gel CC (CH_2_Cl_2_/MeOH 96:4) of the crude provided **22** (16 mg, 25%).

*General procedures for 1,3-thiazolidin-4-ones* **28**–**32**.

*Method A from imines* **23**–**27**. To a solution of the corresponding imine in dry benzene, MgSO_4_ and thioglycolic acid were added, and the reaction was refluxed for the specified time in a flask equipped with a Dean–*S*tark trap under a nitrogen atmosphere. After cooling, the mixture was filtered, concentrated under reduced pressure, diluted with an aq sat NaHCO_3_ solution, and extracted with EtOAc. The combined organic layers were washed with brine, dried over Na_2_SO_4_, and evaporated. Purification of the crude by silica gel CC (CH_2_Cl_2_/EtOAc) provided the corresponding thiazolidinones.

*Method B from aldehyde* **2**. To a suspension of aldehyde **2** in dry benzene was added the appropriate amine, and the mixture was refluxed for the specified time in a flask equipped with a Dean–*S*tark trap under a nitrogen atmosphere. After cooling to room temperature, thioglycolic acid was added dropwise to the solution, and the resulting mixture was refluxed for additional time. It was then cooled and concentrated under reduced pressure. The obtained residue was diluted with an aq sat NaHCO_3_ solution and extracted with EtOAc. The combined organic layers were washed with brine, dried over Na_2_SO_4_, and evaporated. Purification of the crude by silica gel CC (CH_2_Cl_2_/EtOAc) provided the corresponding thiazolidinones.

*1,3-Thiazolidin-4-one* **28**: Following method A from imine **23** (78 mg, 0.17 mmol) in dry benzene (10 mL), MgSO_4_ (43 mg, 0.35 mmol), and thioglycolic acid (26 μL, 0.37 mmol). The mixture was refluxed for 20 h. Purification by silica gel CC (CH_2_Cl_2_/EtOAc 8:2) provided compound **28** (33 mg, 31%) as a 7:3 mixture of epimers. ^1^H NMR (CDCl_3_, 200 MHz) δ (ppm) 6.73/6.71 (*s*, 1H, H6), 6.60/6.56 (*s*, 1H, H3), 6.47/6.55 (*s*, 1H, H7), 6.18/6.28 (*s*, 2H, H2′ and H6′), 5.97/5.94 (*d*, 1H, *J* = 1.4 Hz, H10a), 5.96/5.93 (*d*, 1H, *J* = 1.4 Hz, H10b), 5.27/5.25 (*d*, 1H, *J* = 1.4/1.9 Hz, H9), 4.41/4.32 (*d*, 1H, *J* = 1.8/2.8 Hz, H7′), 3.77/3.80 (*s*, 1H, H11′), 3.73/3.76 (*s*, 6H, H10′ and H12′), 3.66/3.61 (*s*, 3H, CH_3_O-9′), 3.45 (*m*, 2H, H2″), 3.23/3.63 (*d*, 1H, *J* = 1.8/2.8Hz, H8′), 2.08/2.77 and 3.40/3.60 (*m*, 2H, N-CH_2_-CH_3_), 0.95/1.5 (*t*, 3H, *J* = 7.2 Hz, N-CH_2_-CH_3_); ^13^C NMR (CDCl_3_, 200 MHz) Table S4. HRMS calcd for C_27_H_29_NO_8_S [M + H]^+^ 528.1692 u, found 528.1723 *m/z*; IR ν_max_/cm^−1^ (film) 2935, 1732, 1678, 1589, 1505, 1485, 1461, 1417, 1270, 1237, 1126, 1036, 1008, 734.

*1,3-Thiazolidin-4-one* **29**: Following method A from imine **24** (49 mg, 0.10 mmol) in dry benzene (10 mL), MgSO_4_ (43 mg, 0.35 mmol), and thioglycolic acid (8 μL, 0.12 mmol). The mixture was refluxed for 2 h. Purification by silica gel CC (CH_2_Cl_2_/EtOAc 8:2) provided aldehyde **2** (35%) and compound **29** (37 mg, 59%) as a 7:3 mixture of epimers. ^1^H NMR (CDCl_3_, 200 MHz) δ (ppm) 6.72/6.70 (*s*, 1H, H6), 6.58/6.54 (*s*, 1H, H3), 6.46/6.54 (*s*, 1H, H7), 6.15/6.27 (*s*, 2H, H2′ and H6′), 5.96/5.93 (*s*, 1H, H10a), 5.94/5.92 (*s*, 1H, H10b), 5.24 *bs*/5.21 *d* (1H, *J* = 1.8 Hz, H9), 4.39 *bs*/4.30 *d* (1H, *J* = 3.3 Hz, H7′), 3.75/3.78 (*s*, 1H, H11′), 3.71/3.76 (*s*, 6H, H10′ and H12′), 3.64/3.60 (*s*, 3H, CH_3_O-9′), 3.45 (*m*, 2H, H2″), 3.20 *d*/3.46 *bs* (1H, *J* = 1.8 Hz, H8′), 2.08/2.70 and 3.45/3.60 (*m*, 2H, N-CH_2_-CH_2_-CH_3_), 1.40 (*m*, 2H, N-CH_2_-CH_2_-CH_3_), 0.73/0.84 (*t*, 3H, *J* = 7.5 Hz, N-CH_2_-CH_2_-CH_3_); ^13^C NMR (CDCl_3_, 200 MHz) Table S4. HRMS calcd for C_28_H_31_NO_8_S [M + H]^+^ 542.1848 u, found 542.1896 *m/z*; IR ν_max_/cm^−1^ (film) 2958, 1733, 1681-1674, 1589, 1505, 1485, 1463, 1417, 1267, 1238, 1126, 1037, 1009, 735.

Following method B from aldehyde **2** (52 mg, 0.12 mmol) and propylamine (13 μL, 0.16 mmol) in dry benzene (10 mL) for 6 h and thioglycolic acid (9 μL, 0.13 mmol) for an additional 5 h. Purification by silica gel CC (CH_2_Cl_2_/EtOAc 8:2) provided compound **29** (31 mg, 47%) as a 7:3 mixture of epimers. When the reaction was continuously refluxed for 20 h and an additional 8 h, compound **29** was obtained in 59% yield.

1,3-Thiazolidin-4-one **30**: Following method B from aldehyde **2** (50 mg, 0.12 mmol) and ethanolamine (14 μL, 0.23 mmol) in dry benzene (10 mL) for 24 h and thioglycolic acid (10 μL, 0.14 mmol) for an additional 48 h. Purification by silica gel CC (acetone) provided compound **30** (33 mg, 52%) as a 7:3 mixture of epimers. ^1^H NMR (C DCl_3_, 200 MHz) δ (ppm) 6.73/6.71 (*s*, 1H, H6), 6.59/6.60 (*s*, 1H, H3), 6.51/6.55 (*s*, 1H, H7), 6.17/6.26 (*s*, 2H, H2′ and H6′), 5.97/5.94 (*d*, 1H, *J* = 1.5 Hz, H10a), 5.95/5.93 (*d*, 1H, *J* = 1.5 Hz, H10b), 5.41 *bs*/5.45 *d* (1H, *J* = 1.8 Hz, H9), 4.39 *bs*/4.32 *d* (1H, *J* = 3.3 Hz, H7′), 3.75/3.79 (*s*, 1H, H11′), 3.72/3.76 (*s*, 6H, H10′ and H12′), 3.65/3.61 (*s*, 3H, CH_3_O-9′), 3.50 (*m*, 2H, H2″), 3.22 *d*/3.60 *bs* (1H, *J* = 1.8 Hz, H8′), 2.30 and 3.50 (*m*, 2H, N-CH_2_-CH_2_-OH), 3.50–3.80 (*m*, 2H, N-CH_2_-CH_2_-OH); ^13^C NMR (CDCl_3_, 200 MHz) Table S4. HRMS calcd for C_27_H_29_NO_9_S [M + H]^+^ 544.1641 u, found 544.1698 *m/z*; IR ν_max_/cm^−1^ (film) 3450, 2934, 1732, 1678, 1589, 1504, 1485, 1462, 1418, 1269, 1238, 1126, 1036, 1007, 734.

*1,3-Thiazolidin-4-one* **31**: Following method A from imine **26** (87 mg, 0.17 mmol) in dry dichloromethane (10 mL), MgSO_4_ (50 mg, 0.41 mmol), and thioglycolic acid (30 μL, 0.43 mmol). The mixture was kept at rt for 15 d. Evaporation of the solvent and CC on silica gel (CH_2_Cl_2_/EtOAc 1:1) provided compound **31** (31 mg, 31%) as a 7:3 mixture of epimers. ^1^H NMR (CDCl_3_, 200 MHz) δ (ppm) 7.20 (*m*, 5H, phenyl), 6.63/6.67 (*s*, 1H, H6), 6.58/6.53 (*s*, 1H, H3), 6.38/6.51 (*s*, 1H, H7), 6.10/6.17 (*s*, 2H, H2′ and H6′), 5.94 (*d*, 1H, *J* = 1.5 Hz, H10a), 5.92 (*d*, 1H, *J* = 1.5 Hz, H10b), 5.68/5.53 (*s*, 1H, H9), 4.50/4.31 (*d*, 1H, *J* = 2.2/2.8 Hz, H7′), 3.71/3.82 (*s*, 1H, H11′), 3.69/3.80 (*s*, 6H, H10′ and H12′), 3.68/3.51 (*s*, 3H, CH_3_O-9′), 3.51/3.48 (*s*, 2H, H2″), 3.46 *d*/3.52 *bs* (1H, *J* = 2.2 Hz, H8′); ^13^C NMR (CDCl_3_, 200 MHz) Table S4. HRMS calcd for C_31_H_29_NO_8_S [M + H]^+^ 576.1692 u, found 576.1653 *m/z*; IR ν_max_/cm^−1^ (film) 2935, 1732, 1693–1684, 1590, 1504, 1485, 1462, 1270, 1238, 1126, 1036, 1008, 733.

*1,3-Thiazolidin-4-one* **32**: Following method A from imine **27** (252 mg, 0.47 mmol) in dry benzene (15 mL), MgSO_4_ (100 mg, 0.82 mmol), and thioglycolic acid (68 μL, 0.98 mmol). The mixture was refluxed for 72 h. Purification by silica gel CC (CHCl_3_/MeOH 98:2) provided compound **32** (90 mg, 31%) as a 7:3 mixture of epimers. ^1^H NMR (CDCl_3_, 200 MHz) δ (ppm) 6.99/7.12 and 6.75/6.86 (*d*, *J* = 9.0 Hz, AB system, phenyl), 6.64/6.66 (*s*, 1H, H6), 6.55/6.51 (*s*, 1H, H3), 6.40/6.52 (*s*, 1H, H7), 6.07/6.18 (*s*, 2H, H2′ and H6′), 5.93 (*d*, 1H, *J* = 1.2 Hz, H10a), 5.92 (*d*, 1H, *J* = 1.2 Hz, H10b), 5.65/5.53 (*s*, 1H, H9), 4.42/4.31 (*d*, 1H, *J* = 1.6/2.6 Hz, H7′), 3.80/3.81 (*s*, 1H, H11′), 3.66/3.71 (*s*, 6H, H10′ and H12′), 3.65/3.53 (*s*, 3H, CH_3_O-9′), 3.60/3.48 (*s*, 2H, H2″), 3.43 *d*/3.52 *bs* (1H, *J* = 2.0 Hz, H8′), 3.79/3.80 (*s*, 3H, CH_3_O-Ph); ^13^C NMR (CDCl_3_, 200 MHz) Table S4. HRMS calcd for C_32_H_31_NO_8_S [M + H]^+^ 606.1797 u, found 606.1827 *m/z*; IR ν_max_/cm^−1^ (film) 2930, 1732, 1688–1682, 1590, 1512, 1485, 1463, 1270, 1127, 1036, 1009, 735.

### 3.2. Biological Evaluation

A colorimetric type of assay using the sulforhodamine B (*S*RB) reaction was adapted for the quantitative measurement of cell growth and viability, following a previously described method [30]. This assay employs 96-well cell culture microplates of 9 mm diameter. Cell lines derived from different human cancer types were obtained from the American Type Culture Collection (ATCC). Cells were maintained in RPMI 1640 10% Fetal Bovine Serum (FBS) supplemented with 0.1 g/L penicillin and 0.1 g/L streptomycin sulfate and then incubated at 37 °C, 5% CO_2_, and 98% humidity. For the experiments, cells were harvested from subconfluent cultures using trypsin and resuspended in fresh medium before plating.

Cells were seeded in 96-well microtiter plates at 5 × 10^3^ cells per well in aliquots of 195 μL of RPMI medium, and they were allowed to attach to the plate surface by growing in a drug-free medium for 18 h. Afterward, samples were added to aliquots of 5 μL in a range from 10 to 10^−8^ μg/mL, dissolved in DM*S*O:EtOH:Phosphate-Buffered *S*aline (PB*S*) (0.5:0.5:99). After 72 h of exposure, the antitumor effect was measured by the *S*RB methodology: cells were fixed by adding 50 μL of cold 50% (*wt*/*vol*) trichloroacetic acid (TCA) and incubating for 60 min at 4 °C. Plates were washed with deionized water and dried; 100 μL of *S*RB solution (0.4% *wt*/*vol* in 1% acetic acid) was added to each microtiter well and incubated for 10 min at room temperature. Unbound *S*RB was removed by washing with 1% acetic acid. Plates were air-dried, and bound stain was solubilized with tris(hydroxymethyl)aminomethane (Tris) buffer. Optical densities (ODs) were read on an automated spectrophotometric plate reader at a single wavelength of 490 nm. Data analyses were generated automatically by LIM*S* implementation. Using control OD values (C), test OD values (T), and time-zero OD values (T_0_), the drug concentration that causes 50% Growth Inhibition (GI_50_ value) was calculated from the following equation: 100 × [(T − T_0_)/(C − T0)] = 50. Each value represents the mean of triplicate determinations.

## 4. Conclusions

In this work, we report the synthesis and cytotoxic evaluation of several derivatives of podophyllic aldehyde, a cyclolignan easily obtained from the natural compound podophyllotoxin and having very interesting potency and selectivity against several tumoral cell lines. In order to analyze the influence on the activity of the aldehyde function, several chemical modifications were performed on the podophyllic aldehyde oriented toward the enlargement of the cyclolignan skeleton through position C9. These modifications included vinylogue formation, the nucleophilic addition of different carbon and nitrogen nucleophiles, and the incorporation of the aldehyde carbon into several five-membered rings, such as thiazolidinones and benzo-fused azoles, considering benzimidazole, benzothiazole, benzoxazole, and purine or deazapurine systems. The synthesized derivatives were evaluated against several tumoral cell lines. Some of the new cyclolignans were cytotoxic at the nanomolar level, though most of them were less potent and less selective than the parent compound, podophyllic aldehyde, with the most potent being those having a γ-lactone ring with a methyl substituent at C9. An in silico ADME evaluation predicted good gastrointestinal absorption for the majority of them, and only three were predicted to cross the blood–brain barrier. In general, compounds showed good druggability, and a certain correlation between lipophilicity and cytotoxicity was observed. These results indicate that the γ-lactone ring determines the high anticancer potency and that the α,β-unsaturated aldehyde is necessary for the selectivity of these cyclolignans. Further research will be necessary for an in-depth understanding of their mechanisms of action and the structural differences that influence the selectivity degrees of these compounds.

## Data Availability

The data presented in this study are available in article and Supplementary Materials.

## Supplementary Materials

The following supporting information can be downloaded at https://www.mdpi.com/article/10.3390/molecules29071442/s1, Figures S1 and S2: Graphical representations of data obtained by Swissadme platform; Figures S3–S44: NMR spectra for compounds synthesized. Tables S1–S3: Cytotoxicity data (GI_50_ in μM) and data obtained by Swissadme for the synthesized cyclolignans; Tables S4–S16: ^13^C NMR data for thiazolidines and correlations and assignments for several compounds.
