# Supplementary material for: Cytotoxic Cyclolignans Obtained by the Enlargement of the Cyclolignan Skeleton of Podophyllic Aldehyde, a Selective Podophyllotoxin-Derived Cyclolignan
Source: Molecules. 2024 Mar 23;29(7):1442. doi: 10.3390/molecules29071442 (PMC11013179; doi:10.3390/molecules29071442)
Supplement: Supplementary file 1 [file molecules-29-01442-s001.zip › molecules-2926553-supplementary.pdf]

Supplementary Materials

Cytotoxic Cyclolignans Obtained by the Enlargement of the Cyclolignan Skeleton of Podophyllic Aldehyde, a Selective Podophyllotoxin-Derived Cyclolignan

Pablo A. García [1,*], Ángela-Patricia Hernández [1,2], Mª Antonia Gómez-Zurita [1], José M. Miguel del Corral [1], Marina Gordaliza [1], Andrés Francesch [3], Arturo San Feliciano [1,4] and Mª Ángeles Castro [1,*]

[1] Departamento de Ciencias Farmacéuticas, Área de Química Farmacéutica, Facultad de Farmacia, CIETUS/IBSAL, University of Salamanca, Campus Miguel de Unamuno, 37007 Salamanca, Spain; angytahg@usal.es (Á.-P.H.); marintogzf@gmail.com (M.A.G.-Z.); jmmcs@usal.es (J.M.M.d.C.); mliza@usal.es (M.G.); artsf@usal.es (A.S.F.)

[2] Department of Medicine and General Cytometry Service-Nucleus, CIBERONC CB16/12/00400, Cancer Research Centre (IBMCC/CSIC/USAL/IBSAL), 37007 Salamanca, Spain

[3] PharmaMar S.A., Avda de los Reyes, 1, 28770 Colmenar Viejo, Spain; afrancesch@pharmamar.com

[4] Programa de Pós-Graduação em Ciências Farmacêuticas, Universidade do Vale do Itajaí, UNIVALI, Itajaí 88302-901, SC, Brazil

* Correspondence: pabloagg@usal.es (P.A.G.); macg@usal.es (M.A.C.)

Table of contents

Table S1. Cytotoxicity data (GI_{50} in μM) for the *s*ynthe*s*ized cyclolignan*s* 3

Table S2: Swissadme re*s*ult*s* for compound*s* synthesized in thi*s* work. 4

Table S3: Additional Swissadme re*s*ults for compounds synthesized in thi*s* work. 5

Table S4. ^{13}C NMR data for thiazolidine*s* **28-32** 6

Table S5. Correlations and assignments for compound **11** (δ in ppm). 7

Table S6. Correlations and assignments for compound **12** (δ in ppm)**:** 8

Table S7. Correlations and assignments for compound **13** (δ in ppm)**:** 9

Table S8. Correlations and assignments for compound **14** (δ in ppm)**:** 10

Table S9. Correlations and assignments for compound **15** (δ in ppm)**:** 11

Table S10. Correlations and assignments for compound **16** (δ in ppm)**:** 12

Table S11. Correlations and assignments for compound **19** (δ in ppm)**:** 13

Table S12. Correlations and assignments for compound **20** (δ in ppm)**:** 14

Table S13. Correlations and assignments for compound **21** (δ in ppm)**:** 15

Table S14. Correlations and assignments for compound **22** (δ in ppm)**:** 16

Table S15. Correlations and assignments for compound **28** (δ in ppm)**:** 17

Table S16. Correlations and assignments for compound **32** (δ in ppm)**:** 18

Figure S1: Graphical representation of the Log P, TPSA and Log S values of the synthesized compounds, calculated by Swissadme 19

Figure S2: BOILED-Egg model obtained in Swissadme. 20

Figure S3. ^{1}H and ^{13}C NMR spectra for compound **3**. 21

Figure S4. HMQC and HMBC for compound **3**. 22

Figure S5. ^{1}H and ^{13}C for compound **4**. 23

Figure S6. ^{1}H and ^{13}C for compound **5**. 24

Figure S7. ^{1}H and ^{13}C for compound **6**. 25

Figure S8. ^{1}H and ^{13}C for compound **7**. 26
Figure S9. ^{1}H and ^{13}C for compound **8**. 27
Figure S10. ^{1}H and ^{13}C for compound **9** 28
Figure S11. ^{1}H and ^{13}C for compound **9a** 29
Figure S12. ^{1}H and ^{13}C for compound **9b** 30
Figure S13. ^{1}H and ^{13}C for compound **9c** 31
Figure S14. ^{1}H and ^{13}C for compound **10** 32
Figure S15. HMQC and HMBC for compound **10** 33
Figure S16. ^{1}H and ^{13}C for compound **11** 34
Figure S17. HMQC and HMBC for compound **11** 35
Figure S18. ^{1}H and ^{13}C for compound **12** 36
Figure S19. HMQC and HMBC for compound **12** 37
Figure S20. ^{1}H and ^{13}C for compound **13** 38
Figure S21. HMQC and HMBC for compound **13** 39
Figure S22. ^{1}H and ^{13}C for compound **14** 40
Figure S23. HMQC and HMBC for compound **14** 41
Figure S24. ^{1}H and ^{13}C for compound **15** 42
Figure S25. HMQC and HMBC for compound **15** 43
Figure S26. ^{1}H and ^{13}C for compound **16** 44
Figure S27. HMQC and HMBC for compound **16** 45
Figure S28. ^{1}H and ^{13}C for compound **17** 46
Figure S29. ^{1}H and ^{13}C for compound **18** 47
Figure S30. ^{1}H and ^{13}C for compound **19** 48
Figure S31. HMQC and HMBC for compound **19** 49
Figure S32. ^{1}H and ^{13}C for compound **20** 50
Figure S33. HMQC and HMBC for compound **20** 51
Figure S34. ^{1}H and ^{13}C for compound **21** 52
Figure S35. HMQC and HMBC for compound **21** 53
Figure S36. ^{1}H and ^{13}C for compound **22** 54
Figure S37. HMQC and HMBC for compound **22** 55
Figure S38. ^{1}H and ^{13}C for compound **28** 56
Figure S39. HMQC and HMBC for compound **28** 57
Figure S40. ^{1}H and ^{13}C for compound **29** 58
Figure S41. ^{1}H and ^{13}C for compound **30** 59
Figure S42. ^{1}H and ^{13}C for compound **31** 60
Figure S43. ^{1}H and ^{13}C for compound **32** 61
Figure S44. HMQC and HMBC for compound **32** 62

Table S1. Cytotoxicity data (GI_{50} in μM) for the *s*ynthe*s*ized cyclolignan*s*

**Compound**	**A-549**	**HT-29**	**MEL-28**
**1**	0.012	0.012	-
**2**	0.12	0.012	0.23
**3**	0.27	0.27	0.27
**4**	0.22	0.22	0.22
**5**	0.27	0.27	0.27
**6**	10	2.1	12
**7**	2.6	2.6	2.6
**8**	2.3	2.3	2.3
**9**	0.061	0.061	0.061
**9a**	0.024	0.024	0.024
**9b**	0.024	0.024	0.024
**10**	9.7	12	9.7
**11**	2.5	2.5	2.5
**12**	1.9	1.9	1.9
**13**	0.92	0.92	0.92
**14**	9.2	9.2	9.2
**15**	1.7	1.7	-
**16**	>9.7	>9.7	>9.7
**17**	>9.5	>9.5	>9.5
**18**	>9.1	>9.1	>9.1
**19**	>9.4	>9.4	9.4
**20**	>8.4	>8.4	8.4
**21**	1.0	0.50	1.0
**22**	>9.7	>9.7	>9.7
**28**	4.7	0.95	-
**29**	0.92	0.92	-
**30**	0.92	0.92	-
**31**	0.87	0.09	-
**32**	>8.3	0.83	-

Table S2: Swissadme results for compounds synthesized in this work.

**Compound**	MW	Lipinski #violations	#Rotatable bonds	#H-bond acceptors	#H-bond donors	TPSA	Log P
**1**	414.41	0	4	8	1	92.68	2.28
**2**	426.42	0	7	8	0	89.52	2.93
**3**	469.44	0	8	9	0	118.27	2.81
**4**	452.45	0	8	8	0	89.52	3.31
**5**	466.48	0	8	8	0	89.52	3.59
**6**	482.48	0	9	9	0	98.75	3.52
**7**	454.47	0	8	8	1	92.68	3.24
**8**	426.46	0	7	7	2	86.61	3.02
**9**	410.42	0	4	7	0	72.45	3.34
**9a**	410.42	0	4	7	0	72.45	3.37
**9b**	410.42	0	4	7	0	72.45	3.37
**10**	502.46	1	7	10	0	89.52	4.19
**11**	514.53	1	7	8	1	101.13	4.17
**12**	528.55	1	8	8	1	101.13	4.49
**13**	542.58	1	7	8	1	101.13	4.81
**14**	528.55	1	7	8	1	101.13	4.49
**15**	583.42	1	7	8	1	101.13	5.24
**16**	515.51	1	7	9	0	98.48	4.42
**17**	529.54	1	7	9	0	98.48	4.74
**18**	549.96	1	7	9	0	98.48	4.93
**19**	531.58	1	7	8	0	113.58	4.92
**20**	599.57	1	8	11	0	113.58	5.94
**21**	515.51	1	7	9	1	114.02	3.53
**22**	516.50	2	7	10	1	126.91	3.00
**28**	527.59	1	8	8	0	118.06	3.41
**29**	541.61	1	9	8	0	118.06	3.72
**30**	543.59	1	9	9	1	138.29	2.64
**31**	575.63	1	8	8	0	118.06	4.17
**32**	605.65	1	9	9	0	127.29	4.14

Table S3: Additional Swissadme results for compounds synthesized in this work.

**Compound**	GI absorption	BBB permeant	Pgp substrate	CYP1A2 inhibitor	CYP2C19 inhibitor	CYP2C9 inhibitor	CYP2D6 inhibitor
**1**	High	No	No	No	No	No	Yes
**2**	High	No	No	No	Yes	Yes	Yes
**3**	High	No	No	No	Yes	Yes	Yes
**4**	High	No	No	No	Yes	Yes	Yes
**5**	High	No	No	No	Yes	Yes	Yes
**6**	High	No	No	No	Yes	Yes	Yes
**7**	High	No	No	No	Yes	Yes	Yes
**8**	High	No	Yes	No	No	Yes	Yes
**9**	High	Yes	No	No	Yes	Yes	Yes
**9a**	High	Yes	No	No	Yes	Yes	Yes
**9b**	High	Yes	No	No	Yes	Yes	Yes
**10**	High	No	No	No	Yes	Yes	Yes
**11**	High	No	Yes	No	No	Yes	Yes
**12**	High	No	Yes	No	No	Yes	Yes
**13**	High	No	Yes	No	No	Yes	Yes
**14**	High	No	Yes	No	No	Yes	Yes
**15**	Low	No	Yes	No	No	Yes	Yes
**16**	High	No	Yes	No	No	Yes	Yes
**17**	High	No	Yes	No	No	Yes	Yes
**18**	High	No	Yes	No	No	Yes	Yes
**19**	Low	No	Yes	No	No	Yes	Yes
**20**	Low	No	Yes	No	No	Yes	No
**21**	High	No	Yes	No	No	Yes	Yes
**22**	High	No	Yes	No	No	Yes	Yes
**28**	High	No	No	No	Yes	Yes	Yes
**29**	High	No	No	No	Yes	Yes	Yes
**30**	High	No	No	No	No	Yes	Yes
**31**	High	No	Yes	No	Yes	Yes	Yes
**32**	Low	No	Yes	No	No	Yes	Yes

Table S4. ^{13}C NMR data for thiazolidine*s* **28-32**

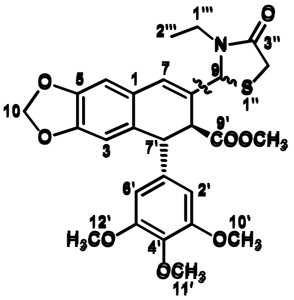
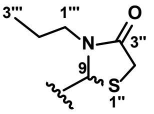
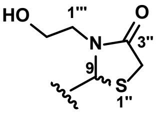
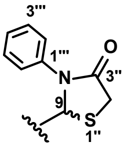
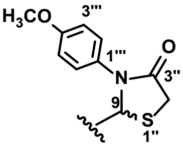

C	**28**	**29**	**30**	**31**	**32**
1	126.4/125.6	127. 6 / 126.5	126.5 / 125.7	126.0	126.0
2	128.6/129.2	128.5	128.2 / 128.5	128.9	128.7
3	109.7/109.4	109.7 / 109.4	109.7 / 109.5	109.6	109.6
4	148.0	148.0	148.0	148.0	148.0
5	147.0/146.9	147.0 / 146.9	147.1	146.9	146.9
6	107.4/107.6	107.4 / 107.6	107.5	107.5	107.5
7	127.5/129.5	127.6 / 129.5	128.5 / 130.3	126.1	126.9 / 128.7
8	129.2/129.5	129.5 / 129.7	129.5 / 129.4	128.9	129.2
9	63.6/64.7	64.1 / 65.1	65.4 / 66.6	66.4	66.7
10	101.2	101.2	101.3	101.2	101.2
1’	137.8/138.0	137.8	137.8	137.8 / 138.0	138.1
2’. 6’	105.1/105.0	104.9	104.9	104.9	104.9
3’. 5’	153.1	153.1	153.1	153.0	153.0
4’	137.2	137.1	137.1	136.9	136.9
7’	46.9/47.6	46.8 / 47.5	46.9 / 47.6	46.5	46.8 / 47.7
8’	48.0/47.2	47.8 / 47.5	48.0 / 47.3	48.9	48.6 / 49.2
9’	172.3/172.0	172.3 / 172.0	172.4	172.3	172.6 / 171.9
10’. 12’	56.1	56.1	56.2	56.0	55.9 / 56.0
11’	60.8	60.8 / 60.3	60.8	60.8	60.8
9’-	52.4	52.4	52.5	52.4	52.4
2’’	32.2/32.9	32.1 / 32.8	31.9 / 32.7	32.7	32.7 / 33.1
3’’	170.9/170.4	171.1 / 170.7	172.1	171.1 / 170.9	170.9
1’’’	36.7/37.3	43.4 / 44.0	44.7	137.8	130.1 / 130.9
2’’’	11.8	19.7 / 19.8	60.8	123.0	125.1
3’’’		11.0 / 14.1		128.9	114.1
4’’’				125.8	157.7
5’’’				128.9	114.1
6’’’				123.0	125.1
4’’’-					55.3 / 55.4

Table S5. Correlations and assignments for compound **11** (δ in ppm).

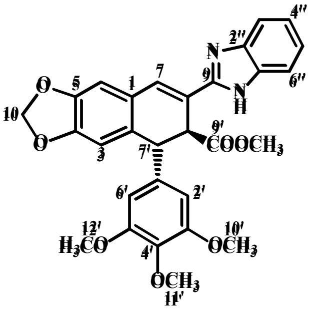

δ ^{13}C	TYPE*	HMQC, δ ^{1}H (*J* in Hz)	HMBC, δ ^{1}H	Assigned
46,1	CH	4,65 d	6,70 s, 6,35 s, 4,50 sa	7’
47,7	CH	4,50 sa	7,38 sa, 4,65 d	8’
52,7	CH_3	3,65 s		9’-OCH_3
56,0	CH_3	3,69 s		3’, 5’-OCH_3
60,7	CH_3	3,74 s		4’-OCH_3
101,3	CH_2	5,95 d, 5,93 d		10
104,8	CH	6,35 s	6,35 s, 4,65 d	2’, 5’
107,9	CH	6,61 sa	7,38 sa	6
109,6	CH	6,70 s	4,65 d	3
122,0	C		4,65 d, 4,50 sa	8
122,7	CH	7,15 m		4’’, 5’’
126,0	C		6,70 s, 4,65 d	1
129,2	CH	7,38 sa	6,61 sa, 4,50 sa	7
130,1	C		7,38 sa, 4,65 d	2
136,7	C		6,35 s, 3,74 s	4’
138,0	C		4,65 d, 4,50 sa	1’
147,0	C		6,70 s, 6,61 sa, 5,95 d, 5,93 d	5
148,3	C		6,70 s, 6,61 sa, 5,95 d, 5,93 d	4
151,5	C		7,38 sa, 4,50 sa	9
153,0	C		6,35 s, 3,69 s	3’, 5’
173,2	C		4,65 d, 4,50 sa, 3,65 s	9’

* Carbon type according to DEPT experiment

Table S6. Correlations and assignments for compound **12** (δ in ppm):

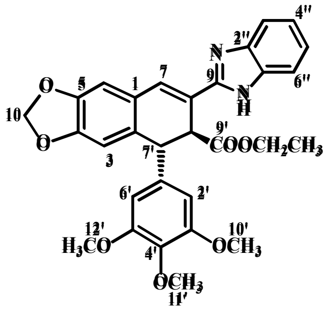

δ ^{13}C	TYPE*	HMQC, δ ^{1}H (*J* in Hz)	HMBC, δ ^{1}H	Assigned
14,0	CH_3	1,14 t	(4,13 dc)	9'-$OCH_2\underline{C}H_3$
46,0	CH	4,64 sa	6,70 s, 6,36 s	7'
48,2	CH	4,39 sa	4,64 sa	8'
56,1	CH_3	3,71 s		3', 5'-OCH_3
60,7	CH_3	3,73 s		4'-OCH_3
61,7	CH_2	4,13 dc	1,14 t	9'-$O\underline{C}H_2CH_3$
101,4	CH_2	5,98 s, 5,97 s		10
104,7	CH	6,36 s	6,36 s, 4,64 sa	2', 6'
108,2	CH	6,76 s		6
109,6	CH	6,70 s		3
123,1	CH / CH	7,16 m		7 / 4'', 5''
125,8	C		6,70 s, (4,64 sa)	1
130,6	C		6,76 s, (4,64 sa), 4,39 sa	2
136,9	C		6,36 s, 3,73 s	4'
137,7	C		4,64 sa	1'
147,0	C		6,76 s, 6,70 s, 5,98 s, 5,97 s	5
148,6	C		6,76 s, 6,70 s, 5,98 s, 5,97 s	4
151,2	CH			9
153,1	C		6,36 s, 3,71 s	3', 5'
172,4	C		(4,39 sa), (4,13 dc)	9'

* Carbon type according to DEPT experiment

Table S7. Correlations and assignments for compound **13** (δ in ppm):

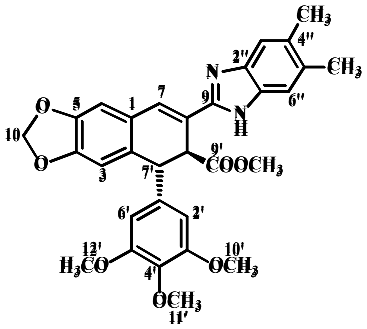

δ ^{13}C	TYPE*	HMQC, δ ^{1}H (*J* in Hz)	HMBC, δ ^{1}H	Assigned
20,3	CH_3	2,30 s		4'', 5''-CH_3
46,2	CH	4,63 d	6,70 s, 6,36 s, 4,48 d	7'
47,7	CH	4,48 d	7,31 s, 4,63 d	8'
52,7	CH_3	3,67 s		9'-OCH_3
56,1	CH_3	3,69 s		3', 5'-OCH_3
60,7	CH_3	3,73 s		4'-OCH_3
101,3	CH_2	5,96 d, 5,95 d		10
104,7	CH	6,36 s	6,36 s, 4,63 d	2', 6'
108,0	CH	6,65 s	7,31 s	6
109,6	CH	6,70 s	4,63 d	3
110,7$^{\#}$	CH		2,30 s	3''
119,2$^{\#}$	CH		2,30 s	6''
122,2	C		4,63 d, 4,48 d	8
126,1	C		6,70 s	1
128,3	CH	7,31 s	6,65 s, 4,48 d	7
130,3	C		7,31 s, 6,65 s, 4,63 d, 4,48 d	2
131,1§	C		2,30 s	4''
132,3§	C		2,30 s	5''
136,8	C		6,36 s, 3,73 s	4'
137,9	C		4,63 d, 4,48 d	1'
146,9	C		6,70 s, 5,96 d, 5,95 d	5
148,2	C		6,65 s, 5,96 d, 5,95 d	4
150,5	C		7,31 s, 4,48 d	9
153,0	C		6,36 s, 3,69 s	3', 5'
173,3	C		4,63 d, 4,48 d, 3,67 s	9'

* Carbon type according to DEPT experiment

$^{\#, §}$ Interchangeable signals

Table S8. Correlations and assignments for compound **14** (δ in ppm):

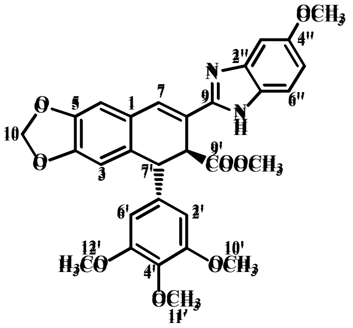

δ ^{13}C	TYPE*	HMQC, δ ^{1}H (*J* in Hz)	HMBC, δ ^{1}H	Assigned
46,1	CH	4,65 d	6,70 s, 6,34 s, 4,46 d	7’
47,7	CH	4,46 d	(7,37 s), 4,65 d	8’
52,8	CH_3	3,69 s		9’-OCH_3
55,6	CH_3	3,81 s		4’’-OCH_3
56,1	CH_3	3,71 s		3’, 5’-OCH_3
60,7	CH_3	3,73 s		4’-OCH_3
101,4	CH_2	5,97 d, 5,96 d		10
104,8	CH	6,34 s	6,34 s, 4,65 d	2’, 6’
108,1	CH	6,66 s		6
109,6	CH	6,70 s	4,65 d	3
112,6	CH	6,78 d		3’’, 6’’
121,4	C		4,65 d, (4,46 d)	8
126,0	C		6,70 s, 4,65 d	1
129,0	CH			7
130,2	C		6,66 s, 4,65 d, 4,46 d	2
136,9	C		6,34 s, 3,73 s	4’
137,9	C		4,65 d, 4,46 d	1’
147,1	C		6,70 s, 5,97 d, 5,96 d	5
148,4	C		6,66 s, 5,97 d, 5,96 d	4
150,8	C		(4,46 d)	9
153,0	C		6,34 s, 3,71 s	3’, 5’
156,7	C		(6,78 d), 3,81 s	4’’
173,4	C		4,65 d, 4,46 d	4

* Carbon type according to DEPT experiment

Table S9. Correlations and assignments for compound **15** (δ in ppm):

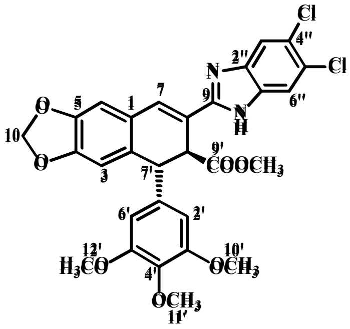

δ ^{13}C	TYPE*	HMQC, δ ^{1}H (*J* in Hz)	HMBC, δ ^{1}H	Assigned
46,3	CH	4,68 d	6,69 s, 6,33 s, 4,66 d	7’
47,6	CH	4,66 d	7,15 s, 4,68 d	8’
53,2	CH_3	3,82 s		9’-OCH_3
56,2	CH_3	3,71 s		3’, 5’-OCH_3
60,7	CH_3	3,74 s		4’-OCH_3
101,5	CH_2	5,97 d, 5,94 d		10
105,1	CH	6,33 s	6,33 s, 4,68 d	2’, 6’
108,2	CH	6,24 s	7,15 s	6
109,6	CH	6,69 s	4,68 d	3
121,3	C		4,68 d, 4,66 d	8
125,6	C		7,15 s, 6,69 s, 4,68 d	1
130,1	C		7,15 s, 6,24 s, 4,68 d	2
130,2	CH	7,15 s	6,24 s, 4,66 d	7
137,1	C		6,33 s, 3,74 s	4’
137,7	C		4,68 d, 4,66 d	1’
147,0	C		6,69 s, 6,24 s, 5,97 d, 5,94 d	5
148,6	C		6,69 s, 6,24 s, 5,97 d, 5,94 d	4
152,9	C		7,15 s, 4,66 d	9
153,1	C		6,33 s, 3,71 s	3’, 5’
174,7	C		7,15 s, 4,68 d, 4,66 d	9’

* Carbon type according to DEPT experiment

Table S10. Correlations and assignments for compound **16** (δ in ppm):

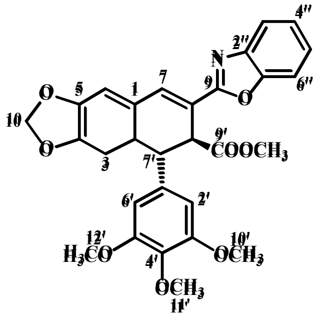

δ ^{13}C	TYPE*	HMQC, δ ^{1}H (*J* in Hz)	HMBC, δ ^{1}H	Assigned
46,2	CH	4,72 d	(6,72 s), 6,30 s, 4,40 d	7'
47,4	CH	4,40 d	7,73 s, 4,72 d	8'
52,7	CH_3	3,65 s		9'-OCH_3
56,0	CH_3	3,72 s		3', 5'-OCH_3
60,7	CH_3	3,75 s		4'-OCH_3
101,5	CH_2	6,01 d, 5,99 d		10
104,8	CH	6,30 s	6,30 s, 4,72 d	2', 6'
108,4	CH	6,90 s	(7,73 s)	6
110,0	CH	6,72 s	4,72 d	3
110,2	CH	(7,49 m)		6''
119,4	C		4,72 d, 4,40 d	8
119,8	CH	(7,67 m)		3''
124,4	CH		7,49 m	4''
126,0	C		6,72 s, (4,72 d)	1
131,0	C		7,73 s, 6,90 s, 4,72 d, 4, 40 d	2
132,7	CH	7,73 s	6,90 s, 4,40 d	7
137,0	C		6,30 s, 3,75 s	4'
137,6	C		4,72 d, 4,40 d	1'
142,1	C		(7,49 m)	2''
147,2	C		(6,90 s), 6,72 s, (6,01 s, 5,99 s)	5
149,0	C		6,90 s, (6,72 s), (6,01 s, 5,99 s)	4
150,5	C		(7,67 m)	1''
153,1	C		6,30 s, 3,72 s	3', 5'
163,0	C		7,73 s, (4,40 d)	9
172,2	C		4,72 d, 4,40 d, 3,65 s	9'

* Carbon type according to DEPT experiment

Table S11. Correlations and assignments for compound **19** (δ in ppm):

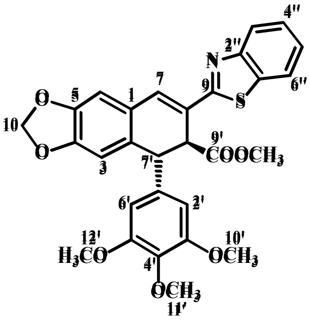

δ ^{13}C	TYPE*	HMQC, δ ^{1}H (*J* in Hz)	HMBC, δ ^{1}H	Assigned
46,5	CH	4,67 d	6,73 s, 6,38 s, 4,61 d	7’
48,3	CH	4,61 d	7,40 s, 4,67 d	8’
52,5	CH_3	3,62 s		9’-OCH_3
56,0	CH_3	3,71 s		3’, 5’-OCH_3
60,7	CH_3	3,75 s		4’-OCH_3
101,4	CH_2	5,99 s, 5,98 s		10
104,6	CH	6,38 s	6,38 s, 4,67 d	2’, 6’
108,2	CH	6,86 s	7,40 s	6
109,8	CH	6,73 s	4,67 d	3
121,3	CH	7,82 m	7,42 m	6’’
123,0	CH	7,92 m	7,34 m	3’’
125,2	CH	7,34 m	7,92 m	5’’
126,0	CH	7,42 m	7,82 m	4’’
126,1	C		6,73 s, 4,67 d	1
127,4	C		4,67 d, 4,61 d	8
131,3	C		7,40 s, 6,86 s, 4,67 d, 4,61 d	2
131,8	CH	7,40 s	6,86 s	7
134,4	C		7,92 m, 7,34 m	2’’
136,8	C		6,38 s, 3,75 s	4’
137,8	C		4,67 d, 4,61 d	1’
147,1	C		6,86 s, 6,73 s, 5,99 s, 5,98 s	5
148,7	C		6,86 s, 6,73 s, 5,99 s, 5,98 s	4
153,0	C		6,38 s, 3,71 s	3’, 5’
153,7	C		7,82 m, 7,42 m	1’’
167,8	C		7,40 s, 4,61 d	9
172,5	C		4,67 d, 4,61 d, 3,62 s	9’

* Carbon type according to DEPT experiment

Table S12. Correlations and assignments for compound **20** (δ in ppm):

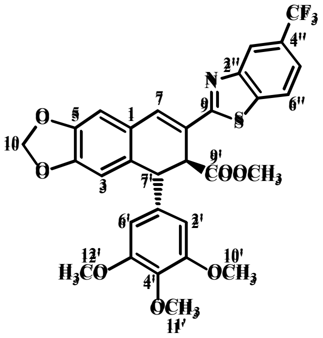

δ ^{13}C	TYPE*	HMQC, δ ^{1}H (*J* in Hz)	HMBC, δ ^{1}H	Assigned
46,5	CH	4,69 d	6,73 s, 6,36 s, 4,58 d	7’
48,4	CH	4,58 d	7,43 s, 4,69 d	8’
52,6	CH_3	3,64 s		9’-OCH_3
56,1	CH_3	3,72 s		3’, 5’-OCH_3
60,7	CH_3	3,75 s		4’-OCH_3
101,5	CH_2	6,01 d, 5,99 d		10
104,6	CH	6,36 s	6,36 s, 4,69 d	2’, 6’
108,3	CH	6,87 s	7,43 s	6
109,9	CH	6,73 s	4,69 d	3
120,1	CH	8,19 s	7,56 dd	3’’
121,5	CH	7,56 dd	8,19 s	5’’
121,9	CH	7,92 d		6’’
125,5	C		8,19 s, 7,56 dd	4’’-CF_3
125,8	C		7,43 s, 6,73 s	1
127,0	C		4,69 d, 4,58 d	8
129,1	C		7,92 d	2’’
131,5	C		7,43 s, 6,87 s, 4,69 d, 4,58 d	2
133,1	CH	7,43 s	6,87 s, 4,58 d	7
137,0	C		6,36 s, 3,75 s	4’
137,7	C		4,69 d, 4,58 d	1’
137,8	C		8,19 s, 7,56 dd	1’’
147,2	C		6,87 s, 6,73 s, 6,01 s, 5,99 s	5
149,0	C		6,87 s, 6,73 s, 6,01 s, 5,99 s	4
153,1	C		6,36 s, 3,72 s	3’, 5’
153,3	C		7,92 d	4’’
169,8	C		7,43 s, 4,58 d	9
172,3	C		4,69 d, 4,58 d, 3,64 s	9’

* Carbon type according to DEPT experiment

Table S13. Correlations and assignments for compound **21** (δ in ppm):

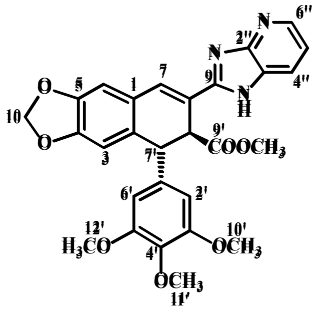

δ ^{13}C	TYPE*	HMQC, δ ^{1}H (*J* in Hz)	HMBC, δ ^{1}H	Assigned
46,2	CH	4,75 d	6,75 s, 6,36 s, 4,61 d	7’
47,5	CH	4,61 d	7,65 s, 4,75 d	8’
52,6	CH_3	3,63 d		9’-OCH_3
55,9	CH_3	3,67 s		3’, 5’-OCH_3
60,6	CH_3	3,72 s		4’-OCH_3
101,5	CH_2	6,02 s, 6,00 s		10
104,6	CH	6,36 s	6,36 s, 4,75 d	2’, 6’
107,9	CH	6,84 s	7,65 s	6
109,9	CH	6,75 s	4,75 d	3
118,2	CH	7,26 m		5’’
122,4	C		4,75 d, 4,61 d	8
126,0	C		6,75 s, 4,75 d	1
130,6	C		7,65 s, 6,84 s, 4,75 d, 4,61 d	2
130,8	CH	7,65 s	6,84 s, 4,61 d	7
136,7	C		6,36 s, 3,72 s	4’
137,7	C		4,75 d, 4,61 d	1’
142,4	CH	8,35 d		4’’
147,2	C		(6,84 s), 6,75 s, 6,02 s, 6,00 s	5
148,7	C		6,84 s, 6,75 s, 6,02 s, 6,00 s	9
153,0	C		6,36 s, 3,67 s	3’, 5’
153,8	C		7,65 s, 4,61 d	9
172,6	C		4,75 d, 4,61 d, 3,63 s	9’

* Carbon type according to DEPT experiment

Table S14. Correlations and assignments for compound **22** (δ in ppm):

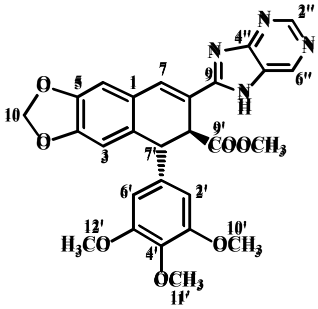

δ ^{13}C	TYPE*	HMQC, δ ^{1}H (*J* in Hz)	HMBC, δ ^{1}H	Assigned
46,1	CH	4,74 d	6,73 s, 6,31 s, 4,55 d	7’
47,6	CH	4,55 d	7,58 s, 4,74 d	8’
52,8	CH_3	3,69 s		9’-OCH_3
56,1	CH_3	3,70 s		3’, 5’-OCH_3
60,7	CH_3	3,73 s		4’-OCH_3
101,6	CH_2	6,01 s, 6,00 s		10
104,8	CH	6,31 s	6,31 s, 4,74 d	2’, 6’
108,2	CH	6,75 s	7,58 s	6
110,0	CH	6,73 s	4,74 d	3
121,4	C		4,74 d, 4,55 d	8
125,4	C		6,73 s, 4,74 d	1
131,0	C		7,58 s, 6,75 s	2
132,3	CH	7,58 s	6,75 s, 4,55 d	7
135,2	C		9,04 s	5’’
137,1	C		6,31 s, 3,73 s	4’
137,5	C		4,74 d, 4,55 d	1’
147,2	CH	9,04 s	8,95 s	6’’
147,3	C		6,73 s, 6,01 s, 6,00 s	5
149,3	C		6,73 s, 6,01 s, 6,00 s	4
151,5	CH	8,95 s	9,04 s	2’’
153,1	C		6,31 s, 3,70 s	9
153,2	C		9,04 s, 8,95 s	4’’
154,4	C		7,58 s, 4,55 d	9
172,5	C		4,74 d, 4,55 d, 3,69 s	9’

* Carbon type according to DEPT experiment

Table S15. Correlations and assignments for compound **28** (δ in ppm):

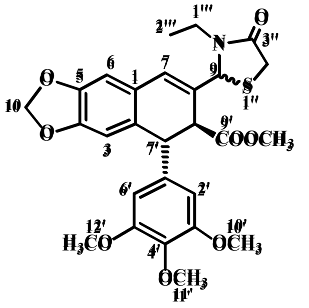

δ ^{13}C	TYPE*	HMQC, δ ^{1}H (*J* in Hz)	HMBC, δ ^{1}H	Assigned
11,8 / 11,8	CH_3	0,95 t / 1,05 t		2'''
32,2 / 32,9	CH_2	3,48 d, 3,45 s / 3,61 d, 3,41 s		2''
36,7 / 37,3	CH_2		0,95 t / 1,05 t	1'''
46,9 / 47,6	CH	4,41 d / 4,32 d	6,18 s / 6,28 s	7'
48,0 / 47,2	CH	3,23 d / 3,63 d	6,47 s/ 6,55 s	8'
52,4 / 52,4	CH_3	3,66 s / 3,61 s		9'-OCH_3
56,1 / 56,1	CH_3	3,73 s / 3,76 s		3', 5'-OCH_3
60,8 / 60,8	CH_3	3,77 s / 3,80 s		4'-OCH_3
63,6 / 64,7	CH	5,27 d / 5,25 d	6,47 s / 6,55 s	9
101,2 / 101,2	CH_2	5,97 d, 5,96 d / 5,94 d, 5,93d		10
105,1 / 105,0	CH	6,18 s / 6,28 s	6,18 s, 4,41 d / 6,28 s, 4,32 d	2', 6'
107,4 / 107,6	CH	6,73 s / 6,71 s	6,47 s / 6,55 s	6
109,7 / 109,4	CH	6,60 s / 6,56 s	4,41 d / (4,32 d)	3
126,4 / 125,6	C		6,60 s, 4,41 d / 6,56 s, (4,32 d)	1
127,5 / 129,5	CH	6,47 s / 6,55 s	6,73 s, 5,27 d / 6,71 s, (5,25 d)	7
128,6 / 129,2	C		6,73 s, 6,47 s / 6,71 s, 6,55 s	2
129,2 / 129,5	C		4,41 d, 3,23 d / 4,32 d, 3,63 d	8
137,2 / 137,2	C		6,18 s, 3,77 s / 6,28 s	4'
137,8 / 138,0	C		4,41 d, 3,23 d / 4,32 d	1'
147,0 / 146,9	C		6,60 s, 5,97 d, 5,96 d / 6,56 s	5
148,0 / 148,0	C		6,73 s / 6,71 s	4
153,1 / 153,1	C		6,18 s, 3,73 s / 6,28 s, 3,76 s	3', 5'
170,9 / 170,4	C		3,48 d, 3,45 s / 3,61 d, 3,41 s	3''
172,3 / 172,0	C		3,66 s, 3,23 d / 3,61 s, 3,63 d	9'

* Carbon type according to DEPT experiment

Table S16. Correlations and assignments for compound **32** (δ in ppm):

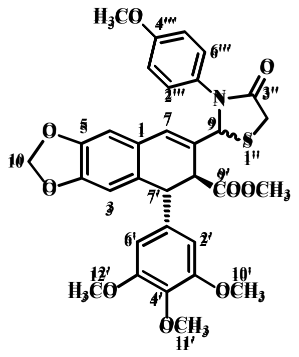

δ ^{13}C	TYPE*	HMQC, δ ^{1}H (*J* in Hz)	HMBC, δ ^{1}H	Assigned
32,7 / 33,1	CH_2	3,60 s / 3,48 s		2’’
46,8 / 47,7	CH	4,42 d / 4,31 d	6,55 s, 6,07 s, 3,43 d / 6,18 s	7’
48,6 / 49,2	CH	3,43 d / 3,52 sa	6,40 s, 5,65 s, 4,42 d / 6,52 s	8’
52,4 / 52,4	CH_3	3,65 s / 3,53 s		9’-OCH_3
55,3 / 55,4	CH_3	3,79 s / 3,80 s		4’’’-OCH_3
55,9 / 56,0	CH_3	3,66 s / 3,71 s		3’, 5’-OCH_3
60,8 / 60,8	CH_3	3,80 s / 3,81 s		4’-OCH_3
66,7 / 66,7	CH	5,65 s / 5, 53 s	6,40 s / 6,52 s	9
101,2 / 101,2	CH_2	5,93 d, 5,92 d / 5,93 d, 5,92 d		10
104,9 / 104,9	CH	6,07 s / 6,18 s	6,07 s, 4,42 d / 6,18 s, (4,31 d)	2’, 6’
107,5 / 107,5	CH	6,64 s / 6,66s	6,40 s / (6,52 s)	6
109,6 / 109,6	CH	6,55 s / 6,51 s	4,42 d	3
114,1 / 114,1	CH	6,75 d / 6,86 d	6,75 d / 6,86 d	3’’’, 5’’’
125,1 / 125,1	CH	6,99 d / 7,12 d	6,99 d / 7,12 d	2’’’, 6’’’
126,0 / 126,0	C		6,55 s, 4,42 d	1
126,9 / 128,7	CH	6,40 s / 6,52 s	6,64 s, 5,65 s, 3,43 d / 6,66 s	7
128,7 / 128,7	C		6,40 s, 3,43 d / 6,52 s	2
129,2 / 129,2	C		4,42 d, 3,43 d	8
130,1 / 130,9	C		6,75 d / 6,86 d	1’’’
136,9 / 136,9	C		6,07 s, 3,80 s / 6,18 s, (3,81 s)	4’
138,1 / 138,1	C		4,42 d, 3,43 d / 4,31 d	1’
146,9 / 146,9	C		6,55 s / 6,51s	5
148,0 / 148,0	C		6,64 s, 5,93 d, 5,92 d	4
153,0 / 153,0	C		6,07 s, 3,66 s / 6,18 s, 3,71 s	3’, 5’
157,7 / 157,7	C		6,99 d, 6,75 d, 3,79 s / 7,12 d, 6,86 d, 3,80 s	4’’’
170,9 / 170,9	C		5,65 s	3’’
172,6 / 171,9	C		4,42 d, 3,65 s, 3,43 d / 3,53 s, 3,52 sa	9’

* Carbon type according to DEPT experiment

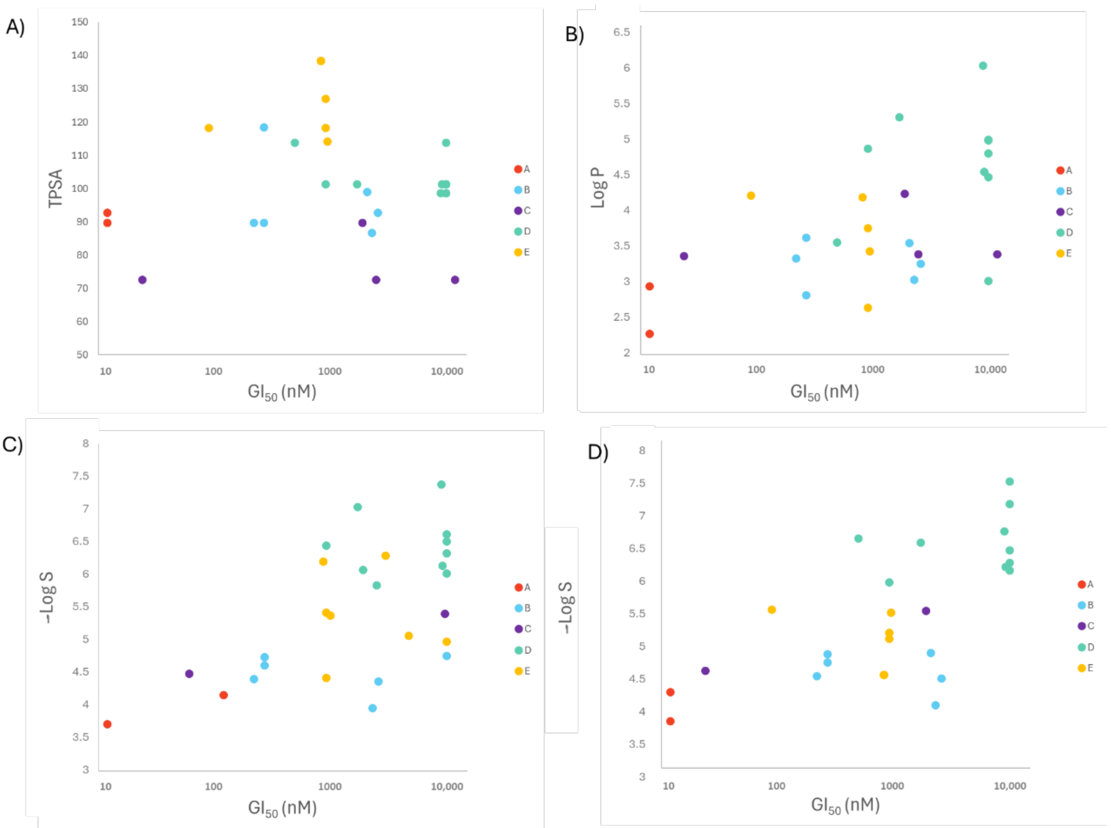

Figure S1: Graphical representation of the Log P, TPSA and Log S values of the synthesized compounds, calculated by Swissadme.

A) and B) TPSA and Log P values respectively, plotted with the cytotoxicity values of the compounds in A549 (expressed in nanomolar). C) and D) Log S plotted with the cytotoxicity values of the compounds in A549 and HT-29 respectively (expressed in nanomolar). Color code for the different subfamilies of compounds synthesized in this work as it is in the manuscript.

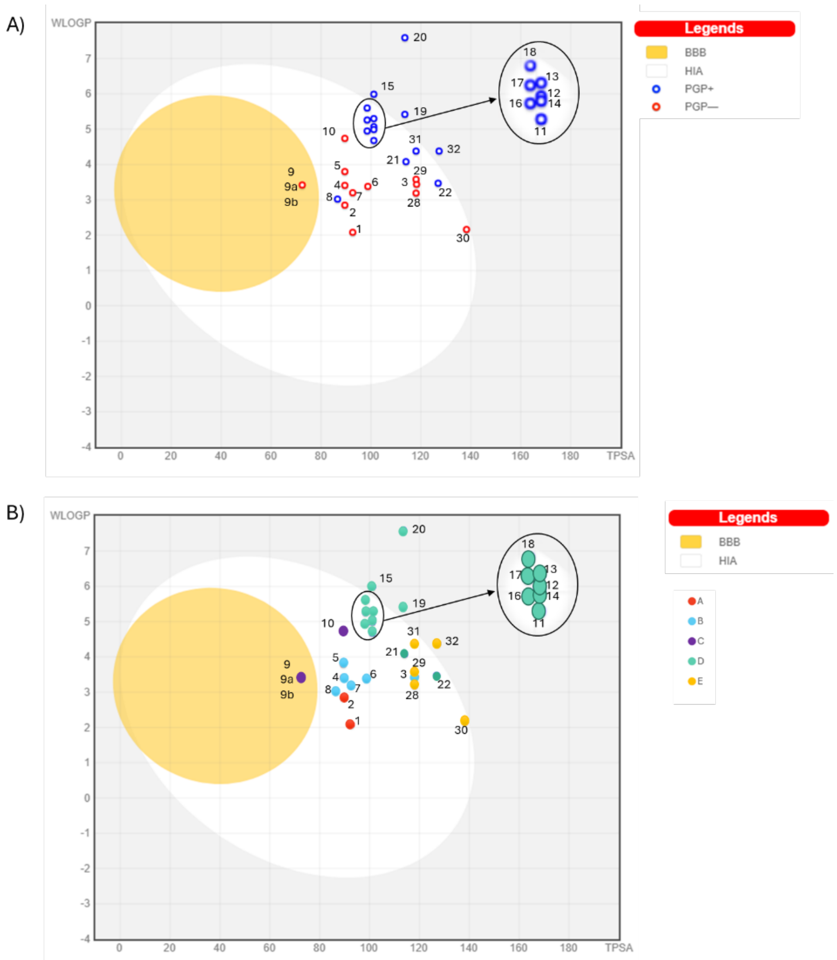

Figure S2: BOILED-Egg model obtained in Swissadme.

Yellow region indicates the compounds that are predicted to passively permeate through the blood-brain barrier (BBB). White region indicates a high intestinal absorption (HIA). In panel A, red points refer to compounds that are not predicted to be substrate of P-glycoprotein and blue points compound predicted to be substrate of P-glycoprotein. In panel B, color code for the different subfamilies of compounds synthesized in this work as it is in the manuscript.

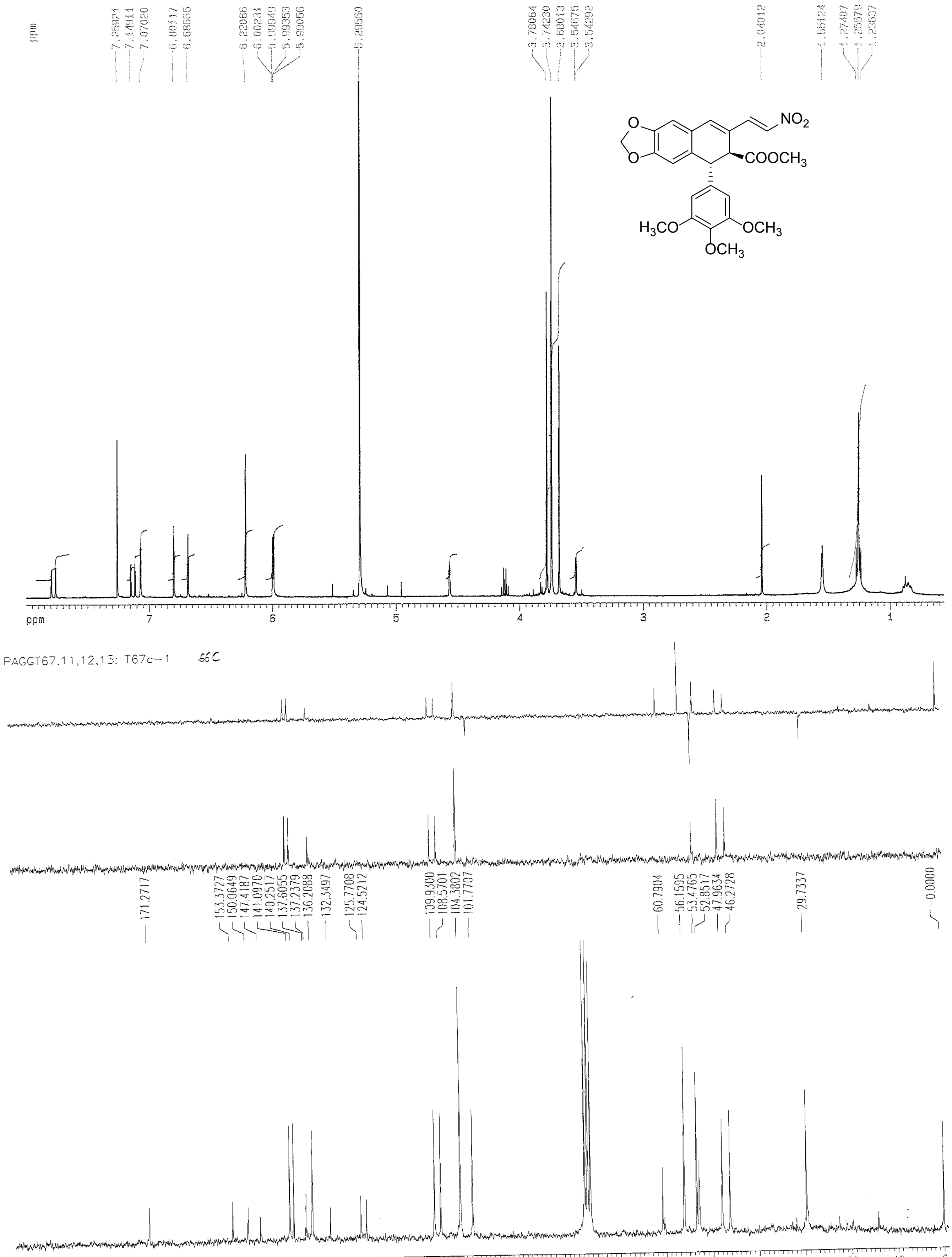

Figure S3. ^{1}H and ^{13}C NMR spectra for compound **3**.

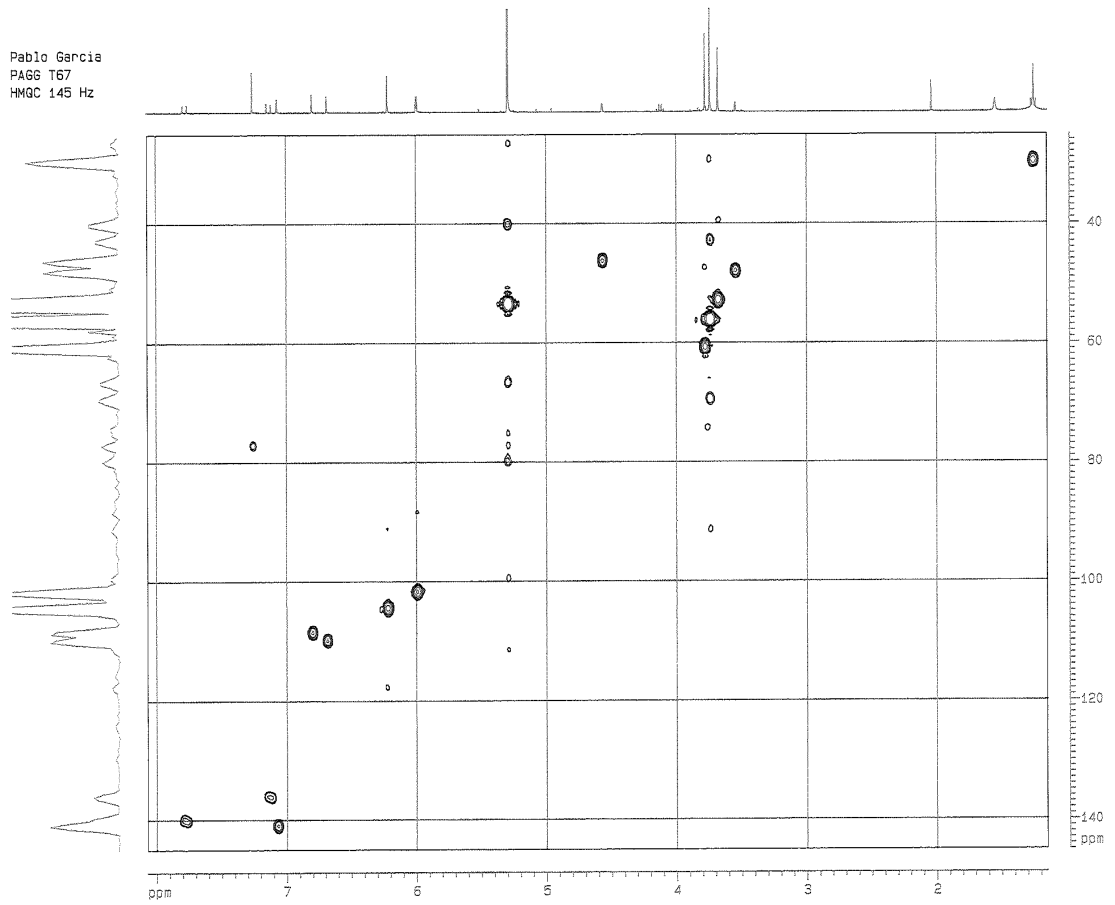

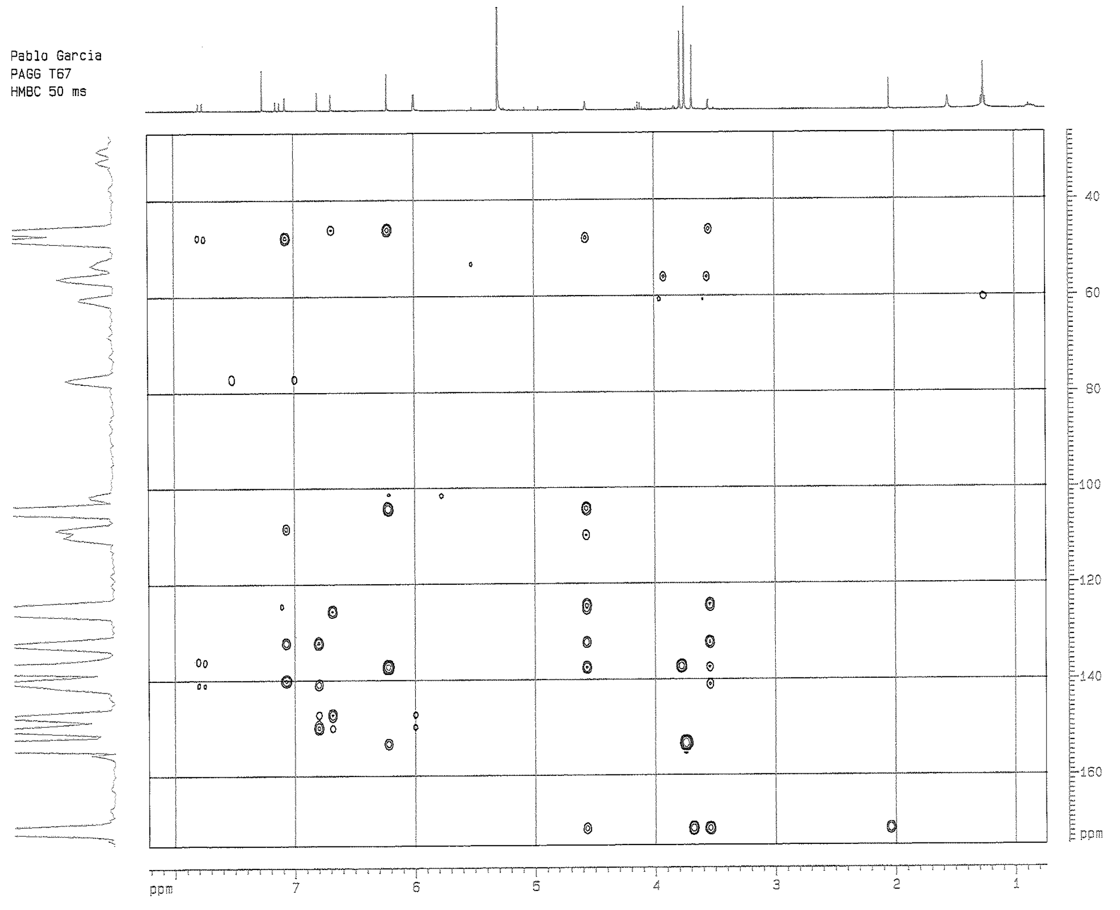

Figure S4. HMQC and HMBC for compound **3**.

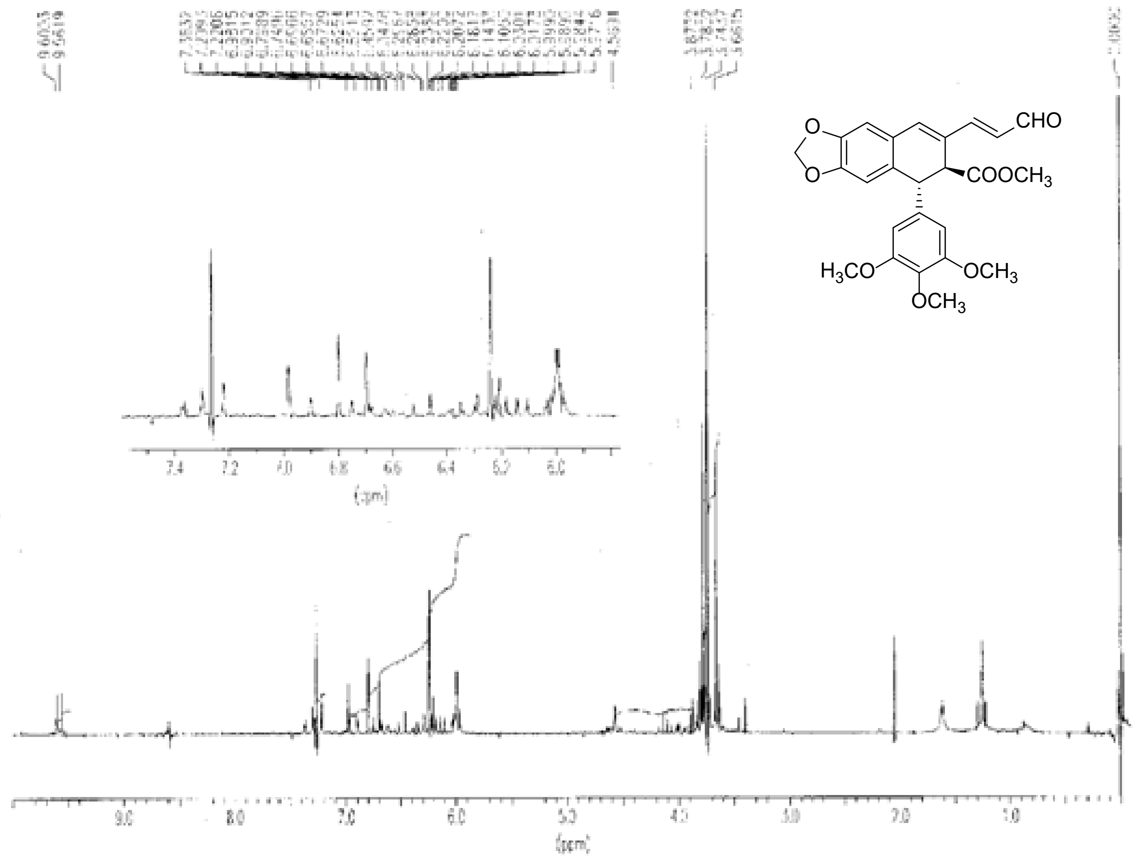

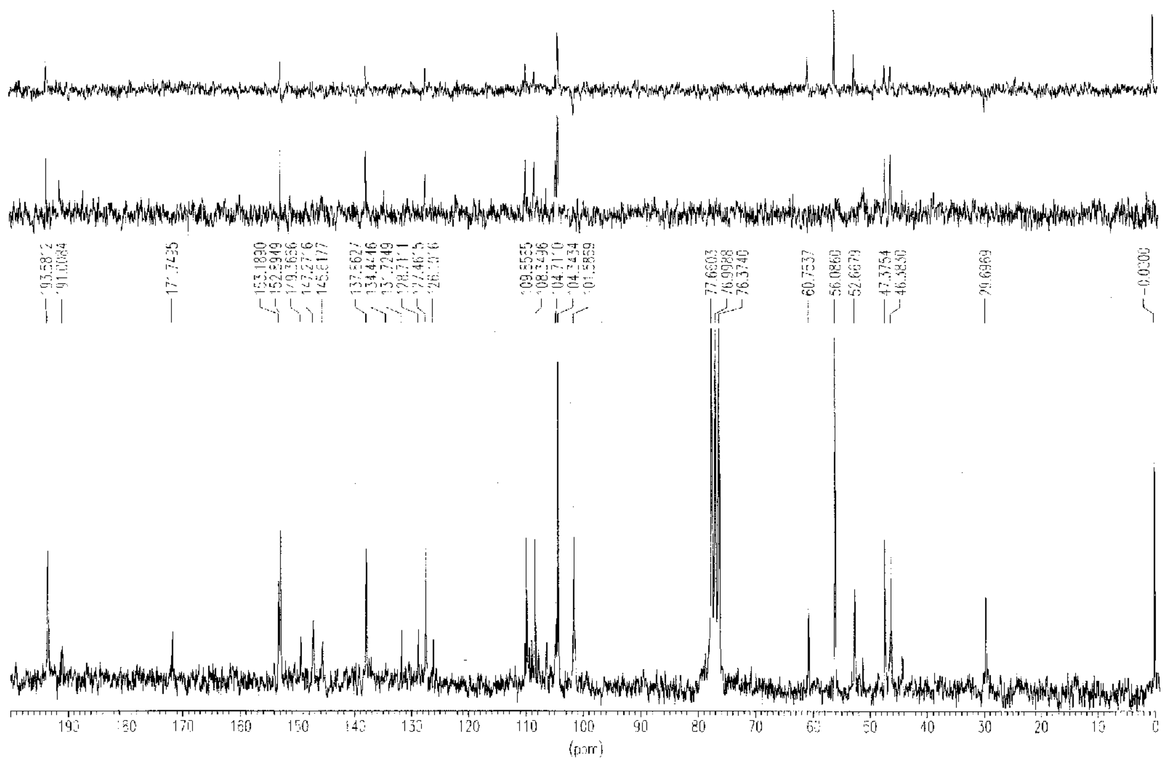

Figure S5. ^{1}H and ^{13}C for compound **4**.

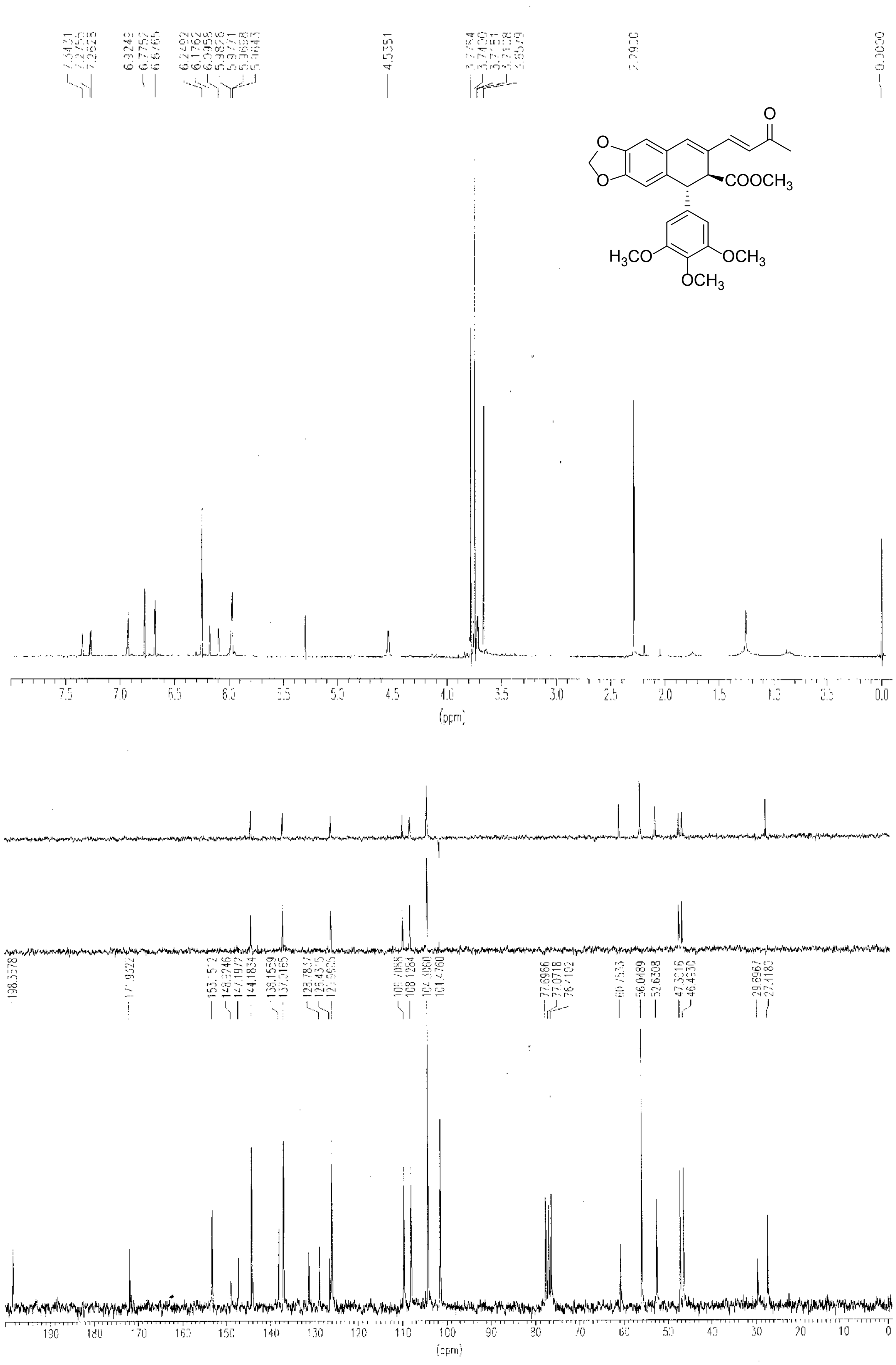

Figure S6. ^{1}H and ^{13}C for compound **5**.

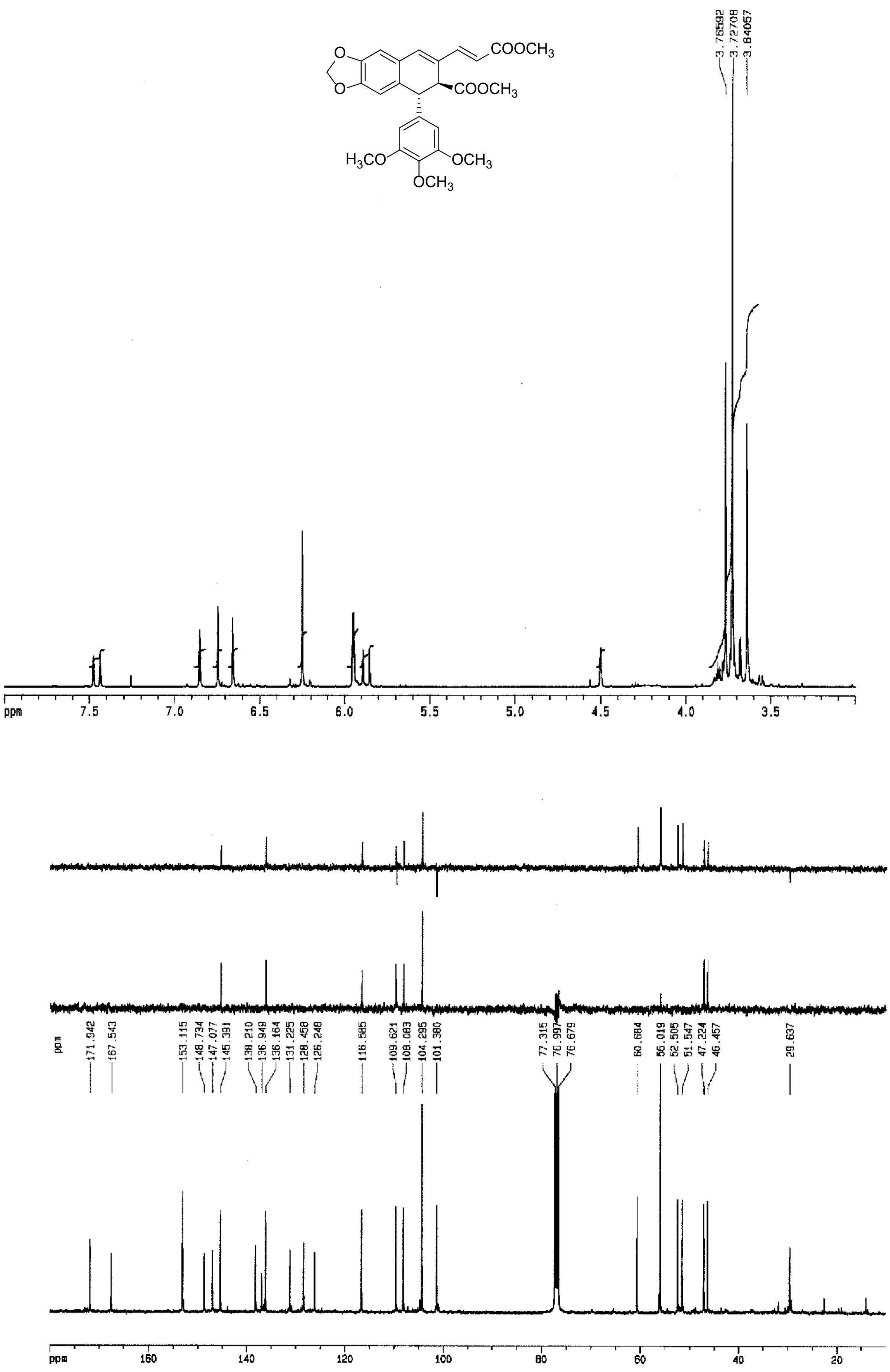

Figure S7. ^{1}H and ^{13}C for compound **6**.

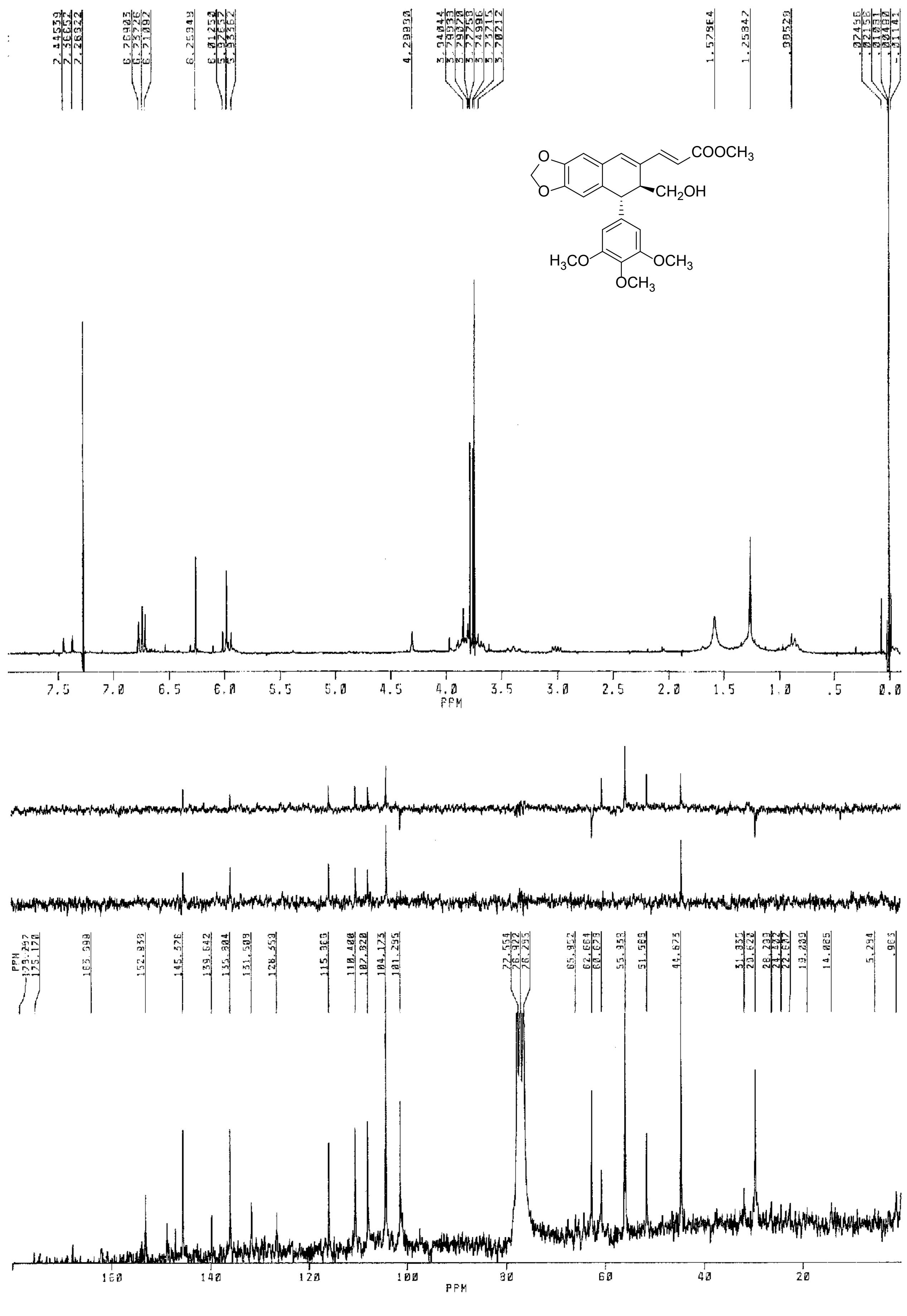

Figure S8. ^{1}H and ^{13}C for compound **7**.

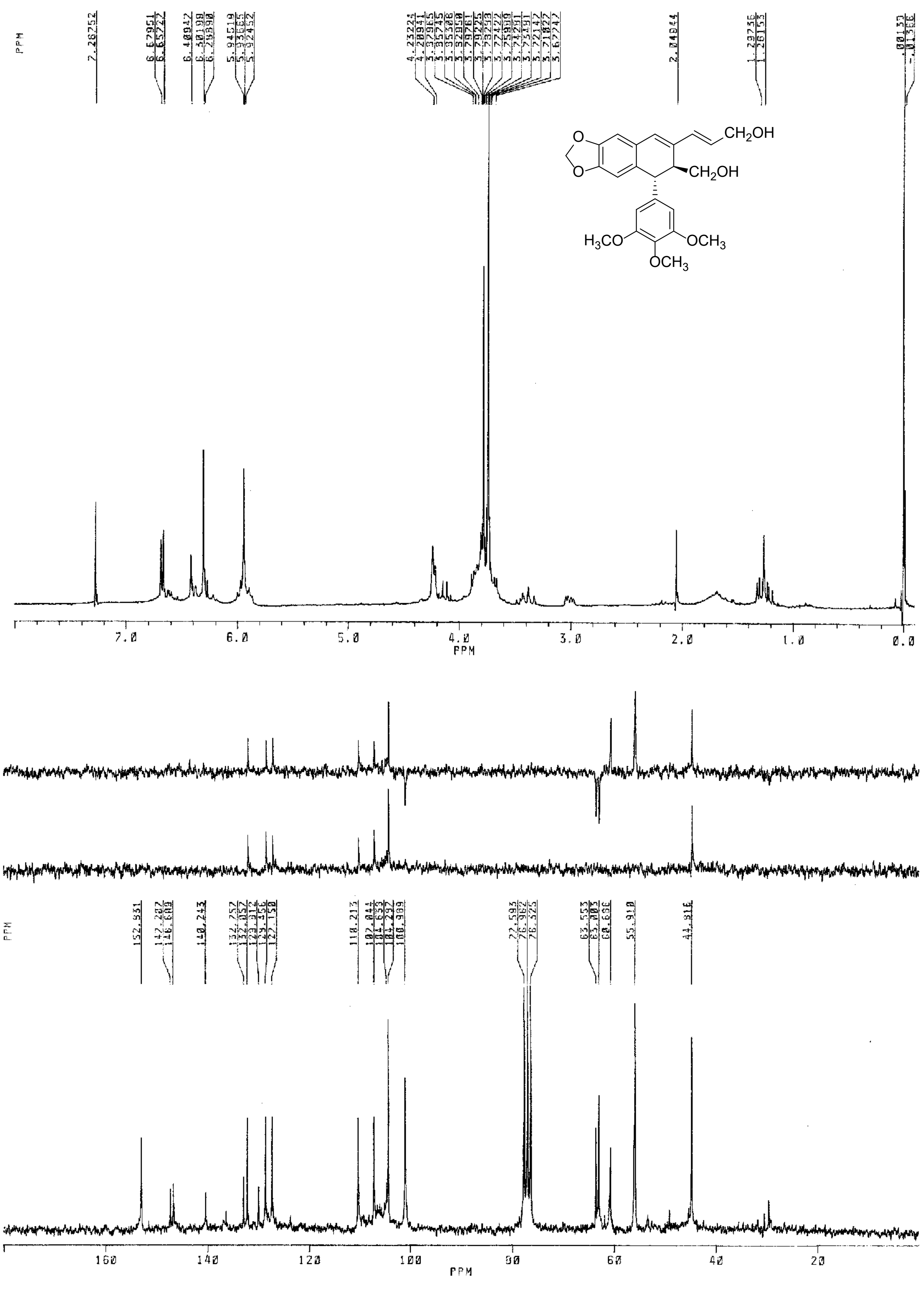

Figure S9. [1]H and ^{13}C for compound **8**.

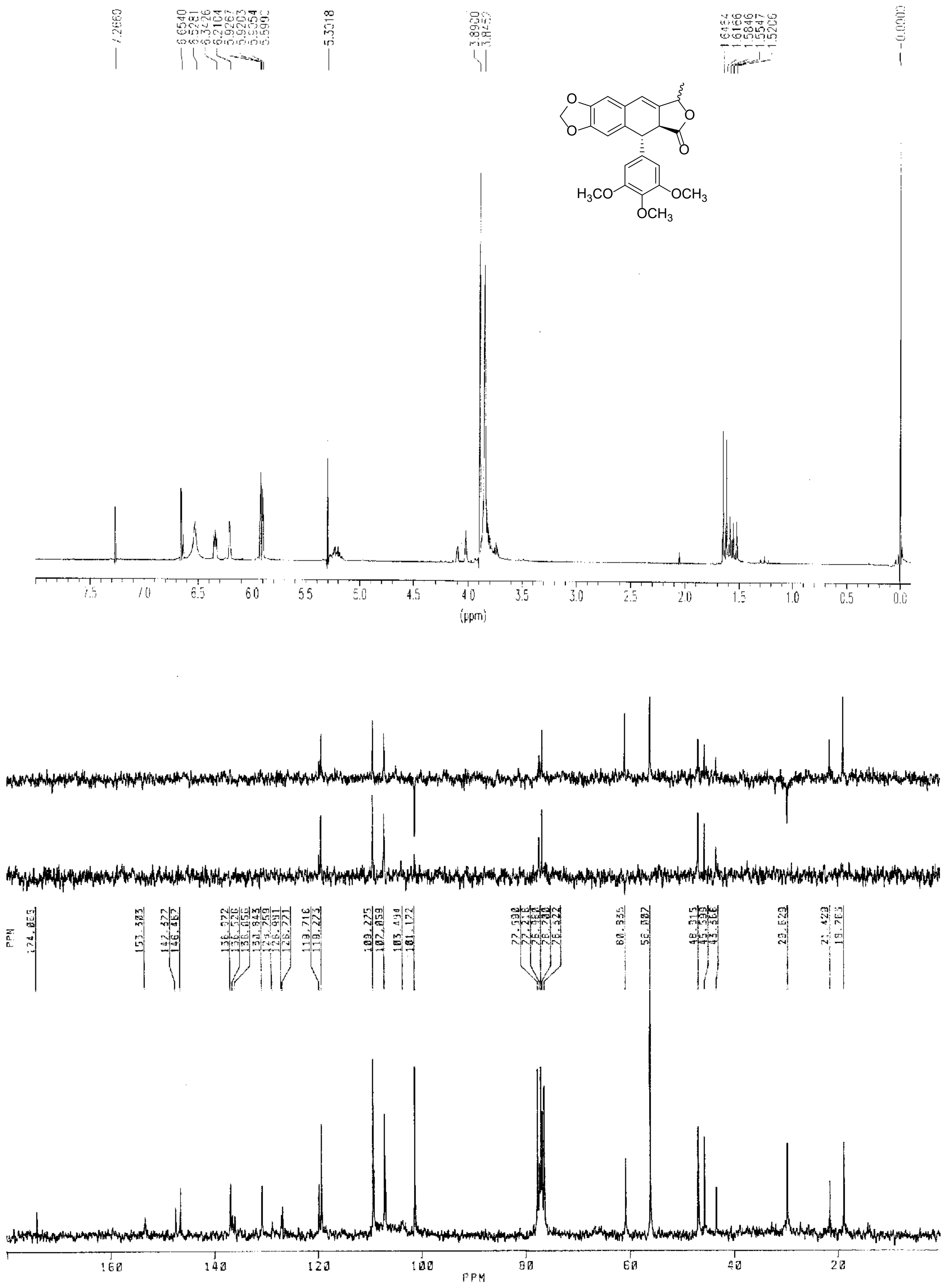

Figure S10. ^{1}H and ^{13}C for compound **9**

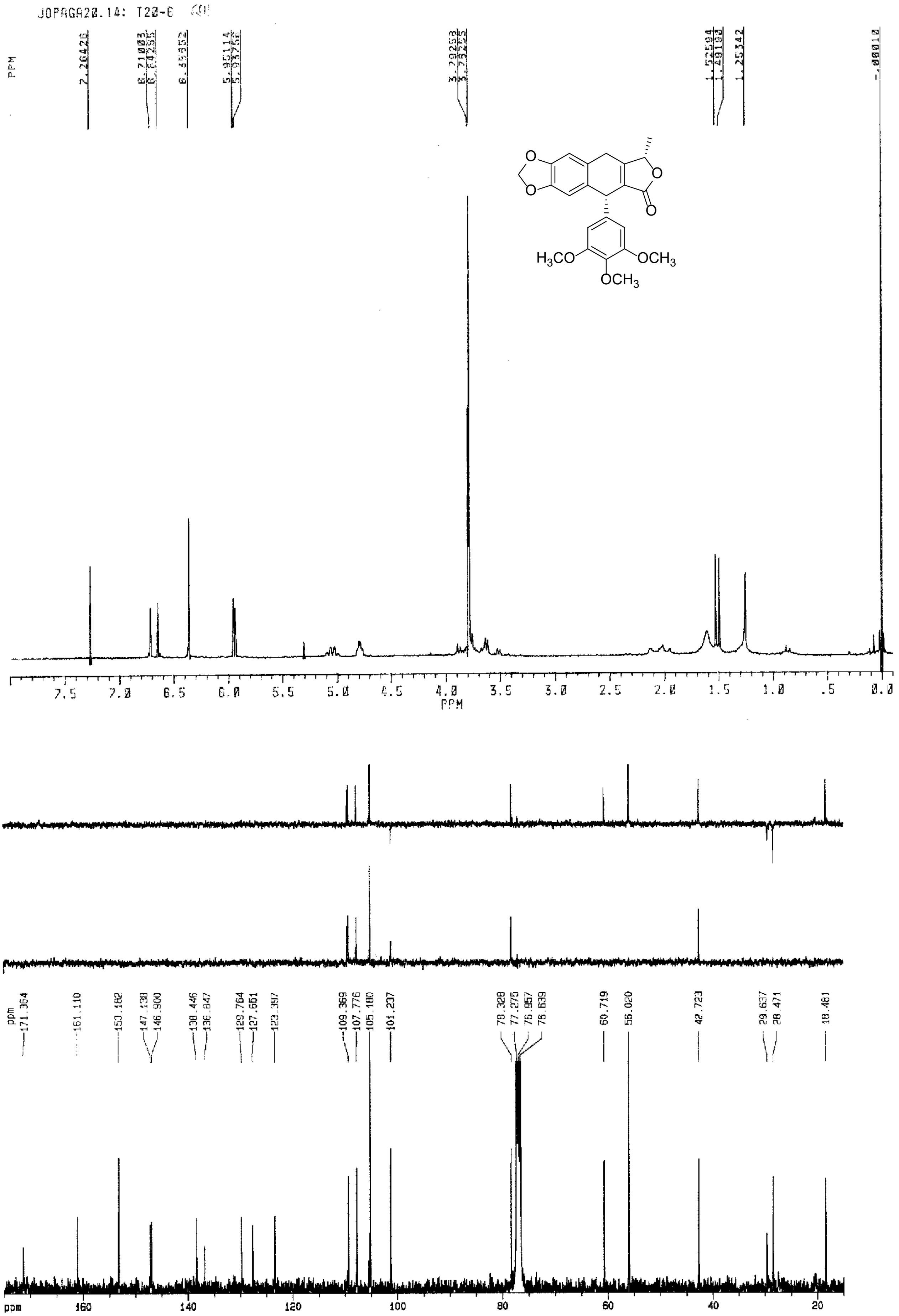

Figure S11. ^{1}H and ^{13}C for compound **9a**

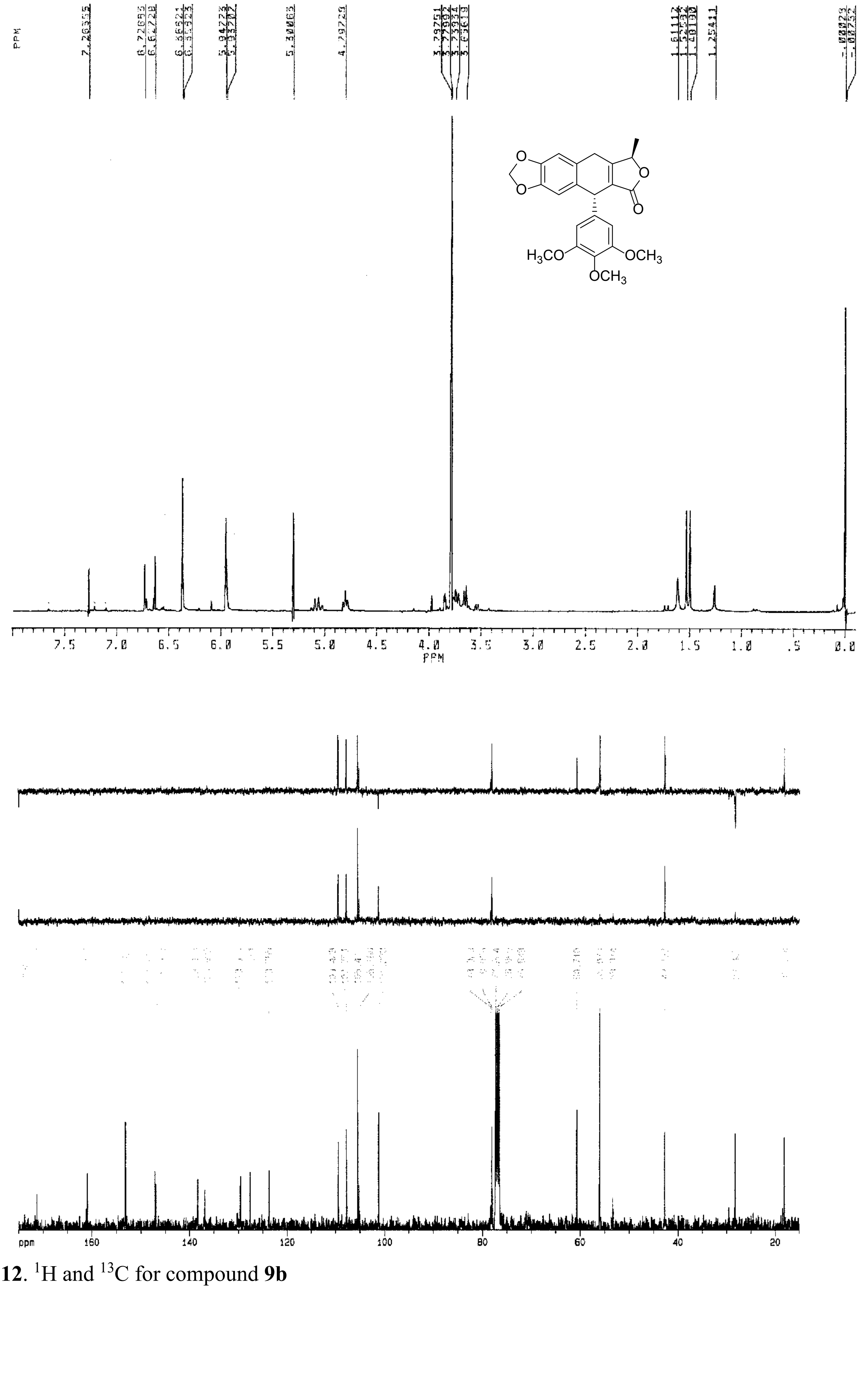

Figure S12. ^{1}H and ^{13}C for compound **9b**

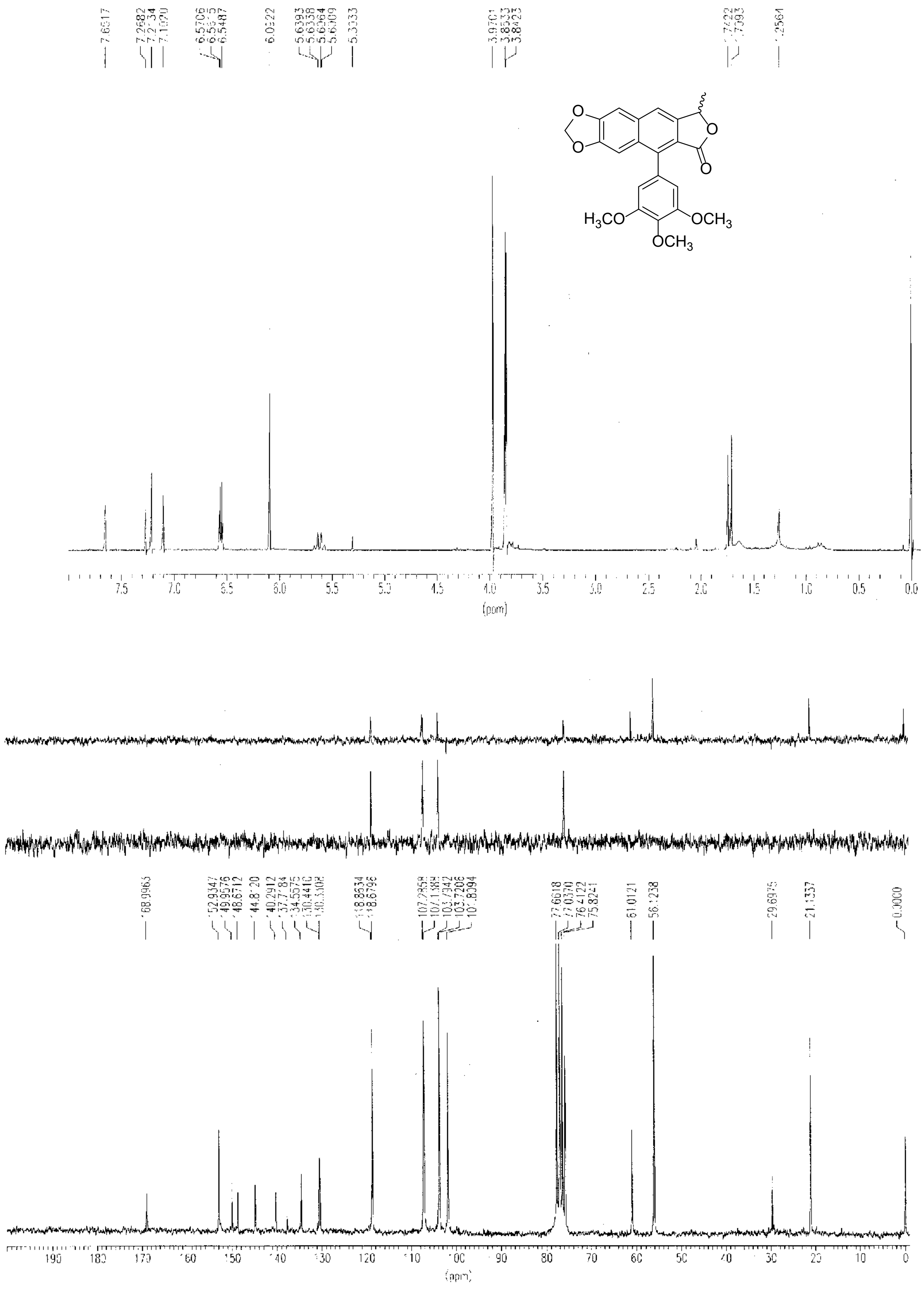

Figure S13. ^{1}H and ^{13}C for compound **9c**

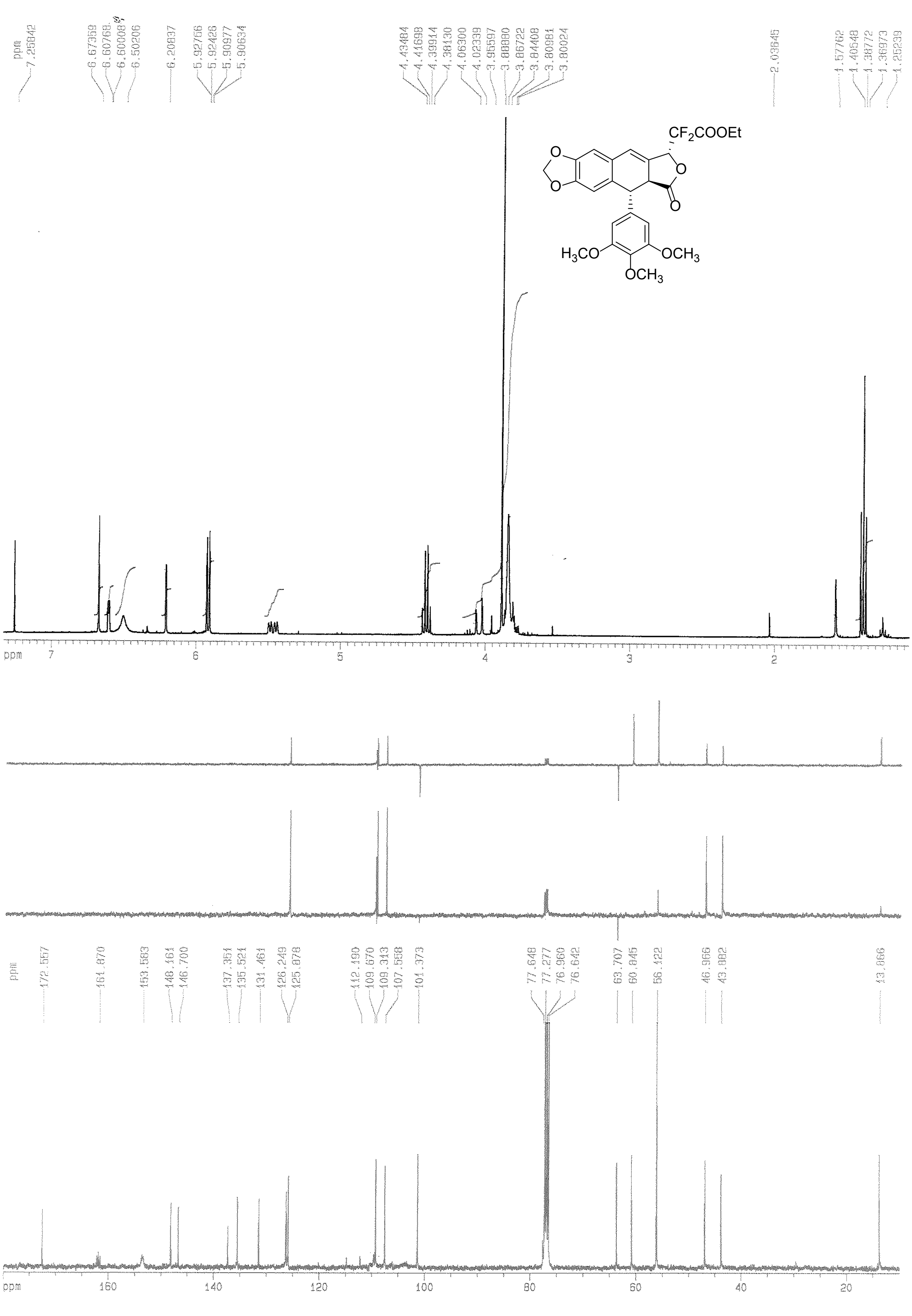

Figure S14. ^{1}H and ^{13}C for compound **10**

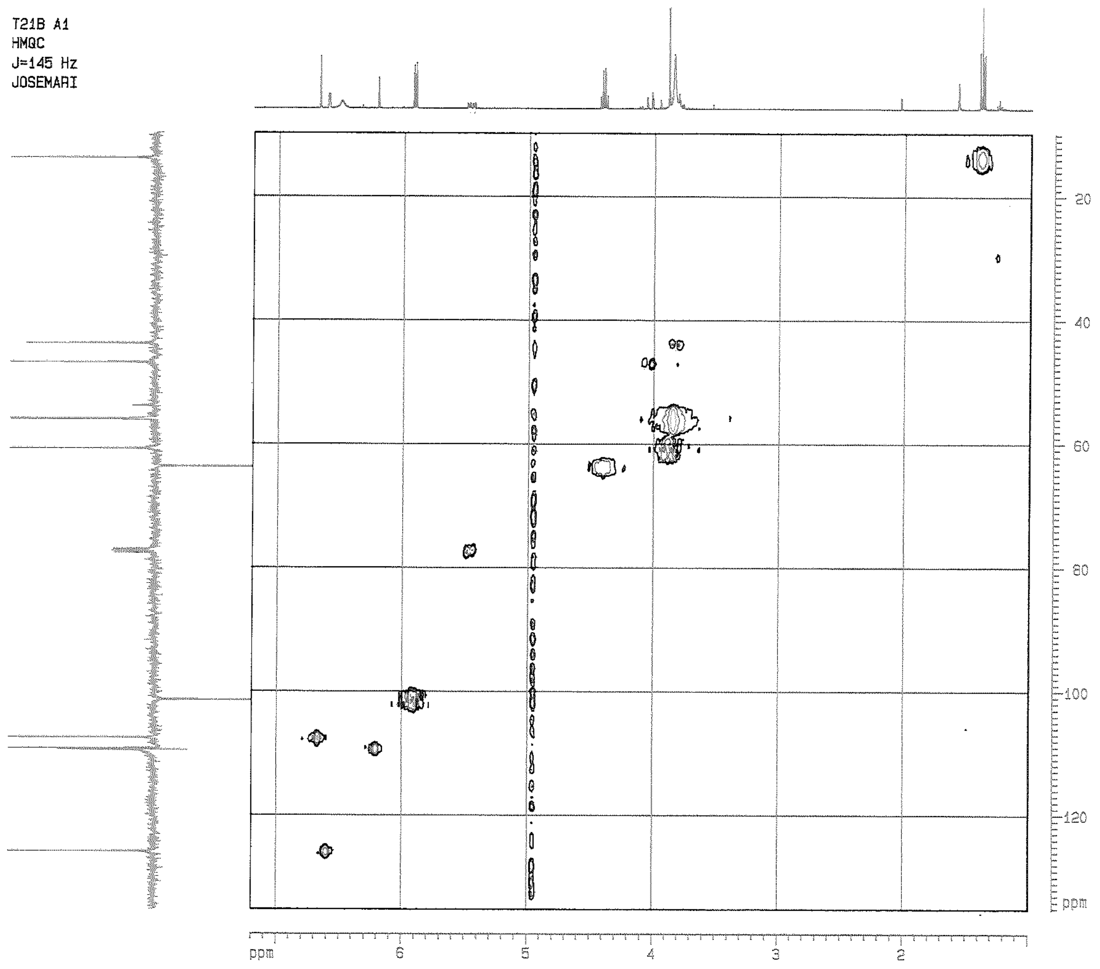

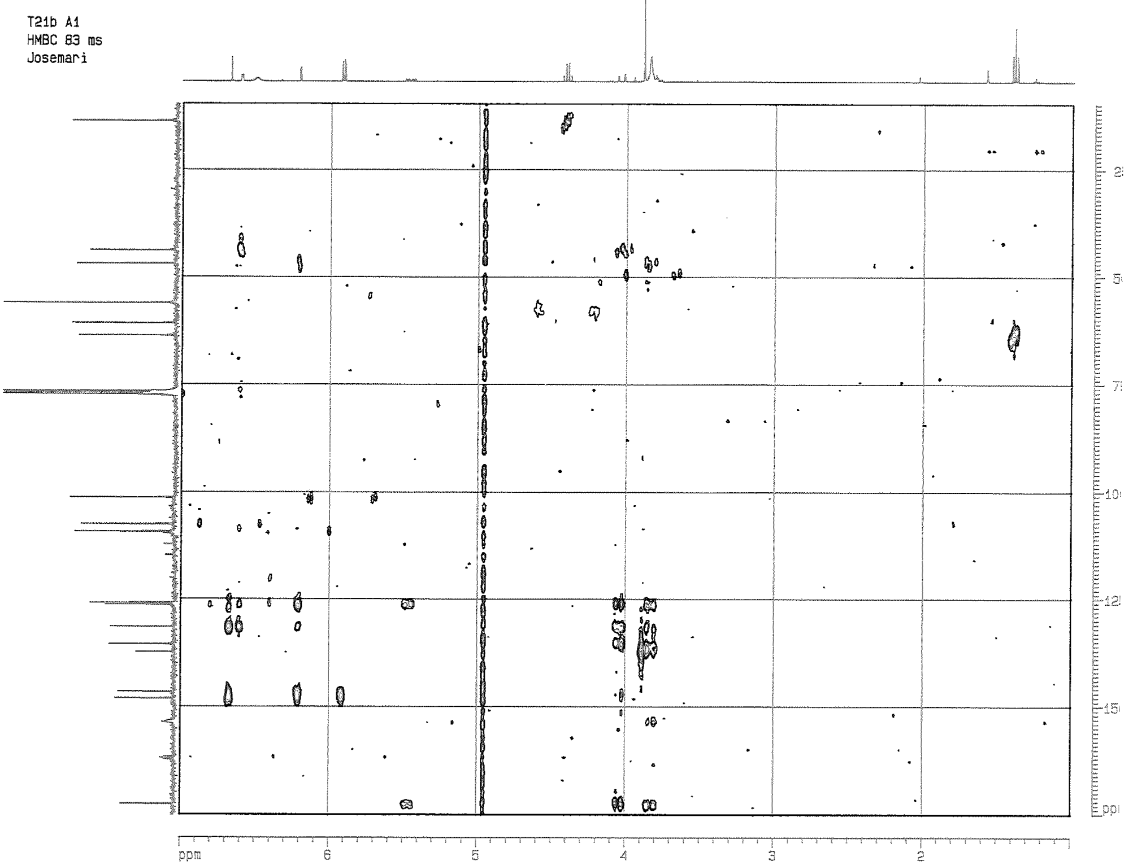

Figure S15. HMQC and HMBC for compound **10**

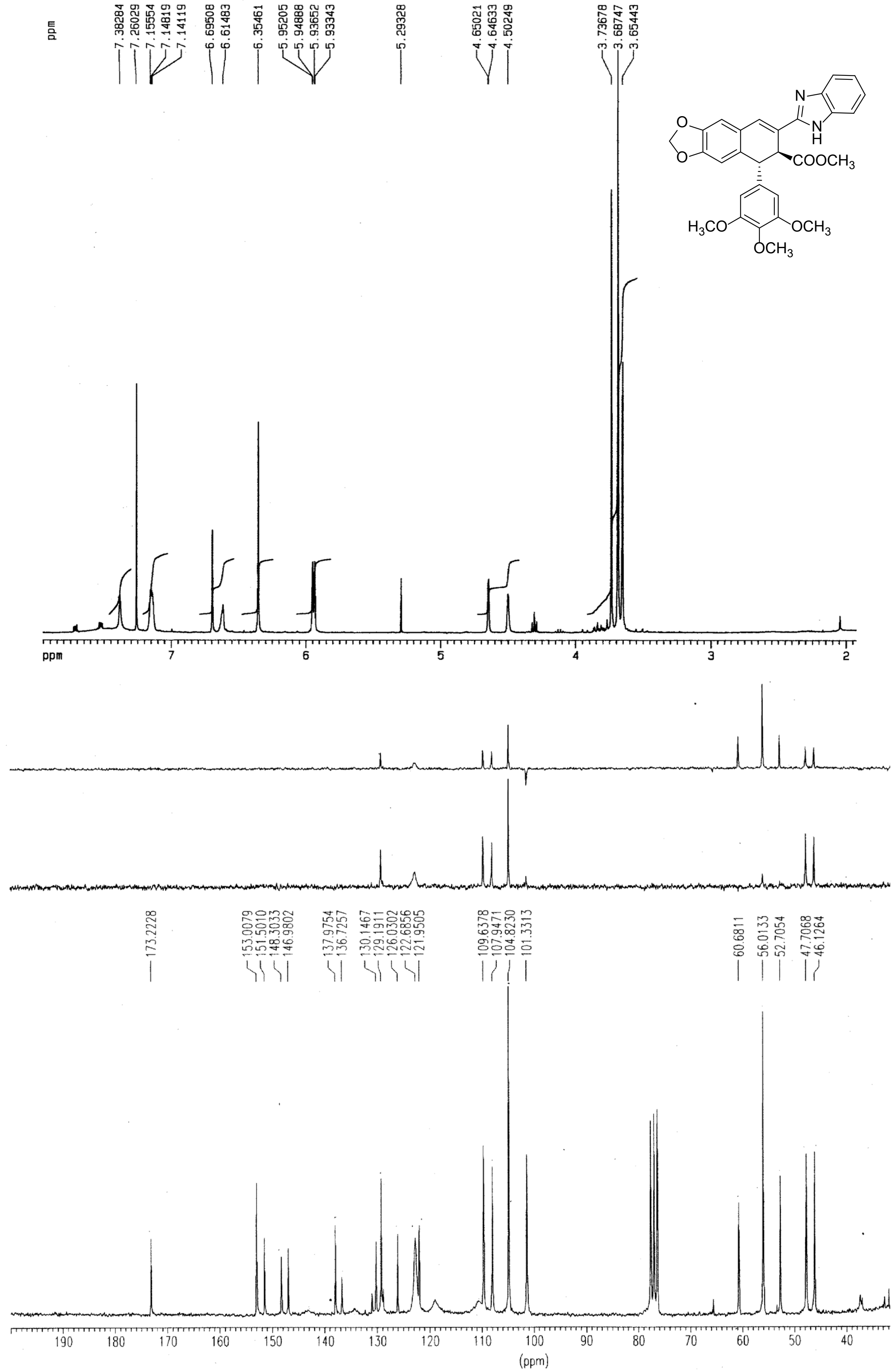

Figure S16. ^{1}H and ^{13}C for compound **11**

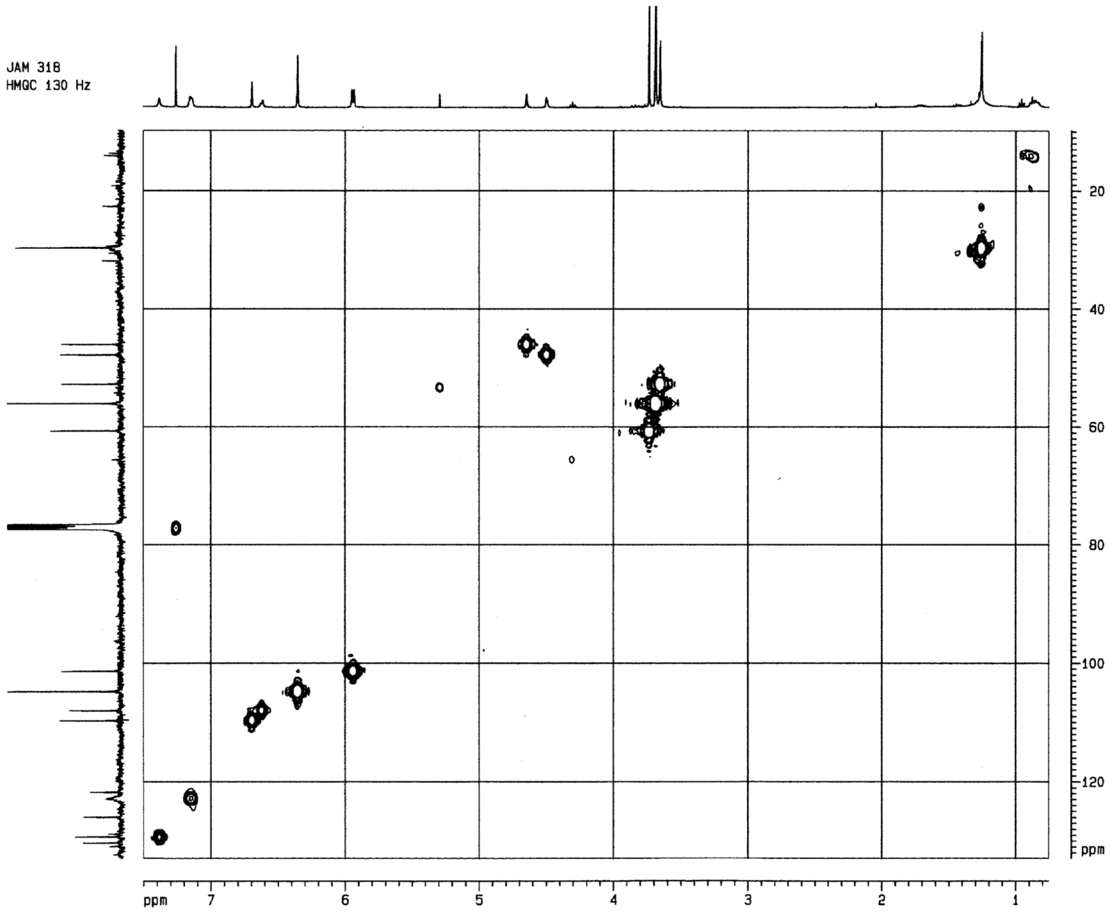

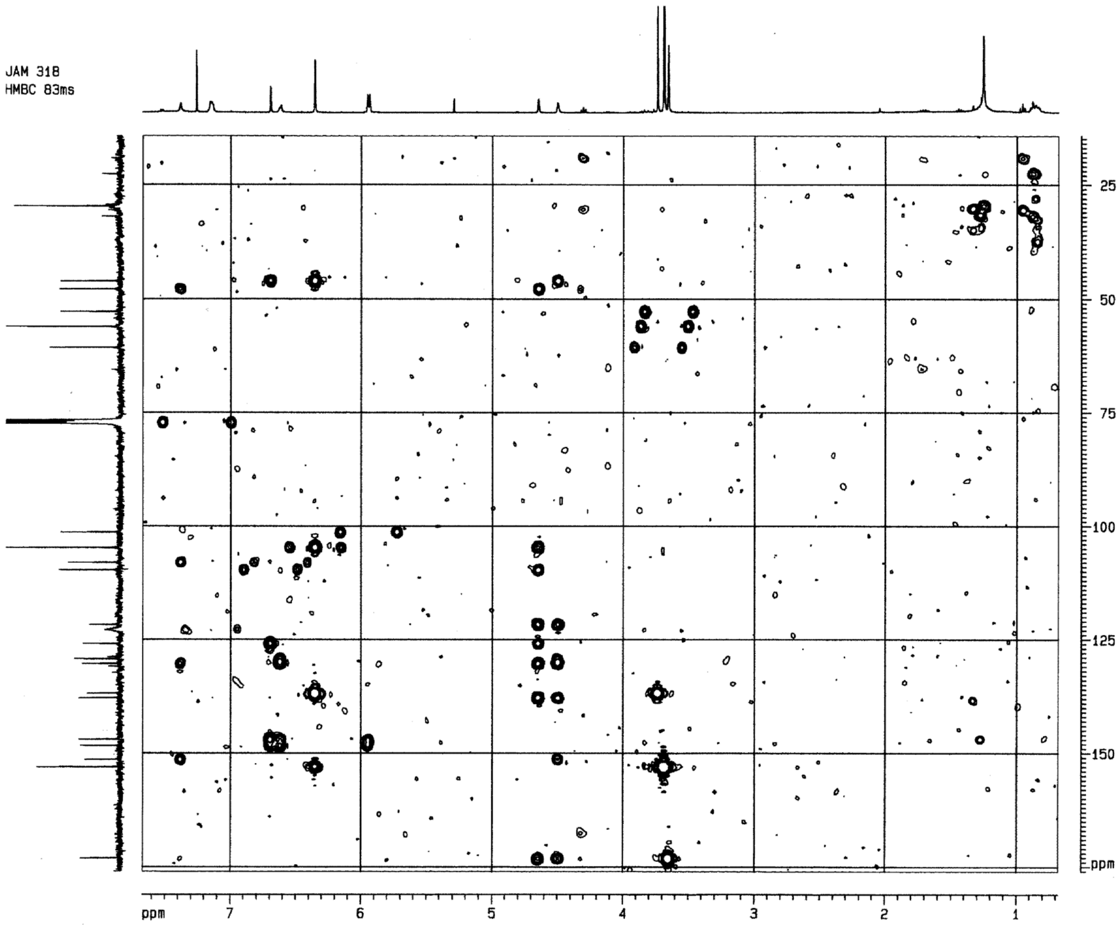

Figure S17. HMQC and HMBC for compound **11**

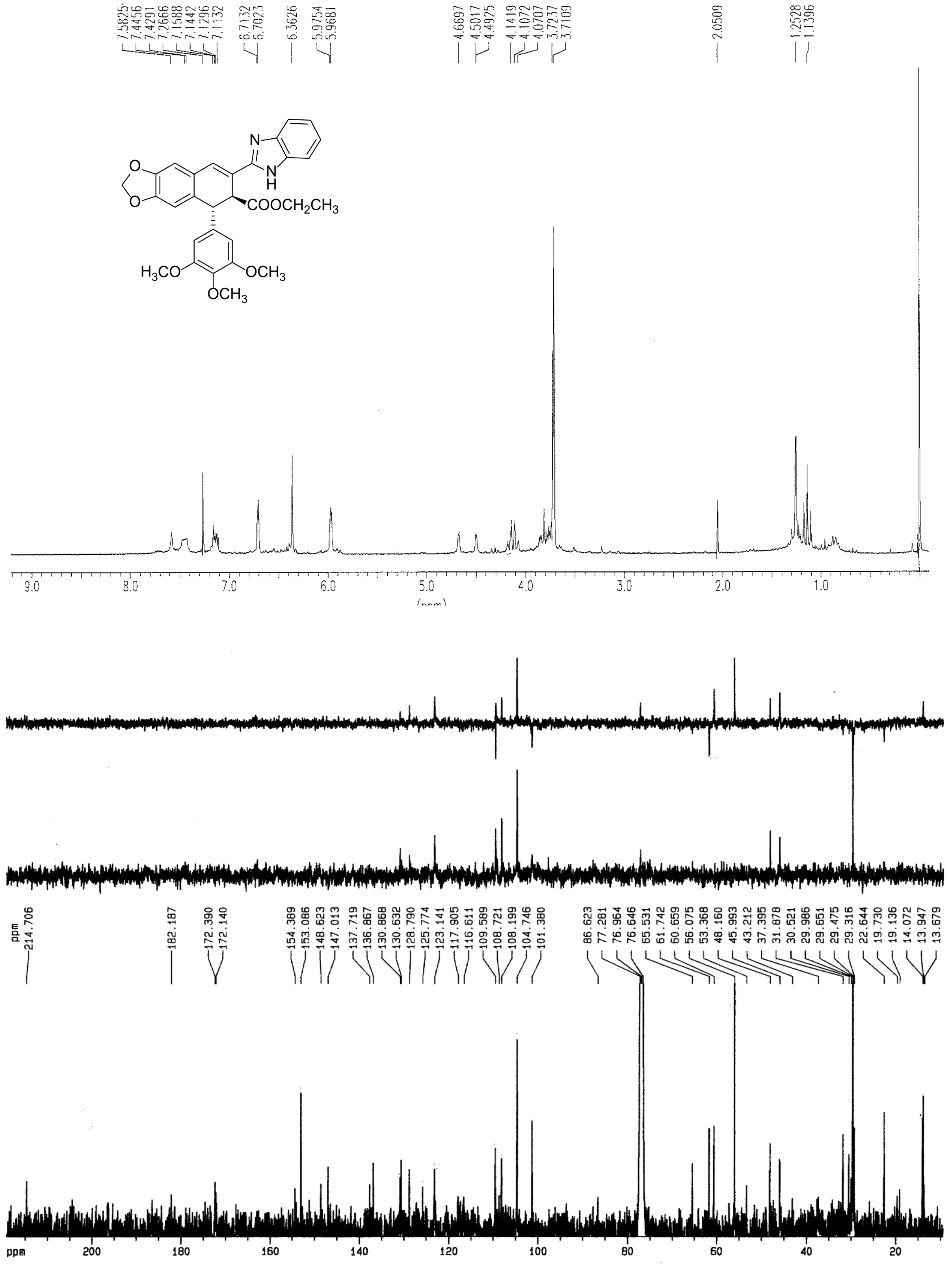

Figure S18. ^{1}H and ^{13}C for compound **12**

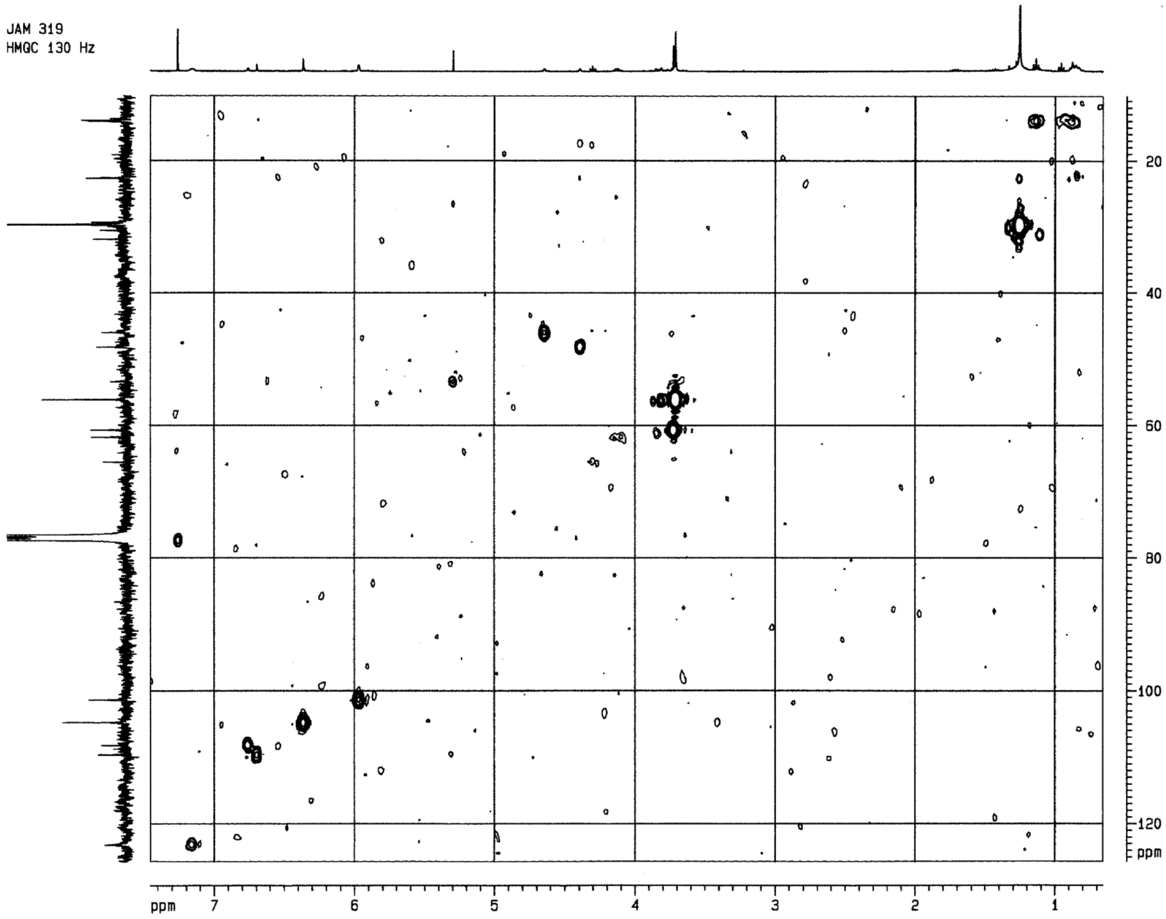

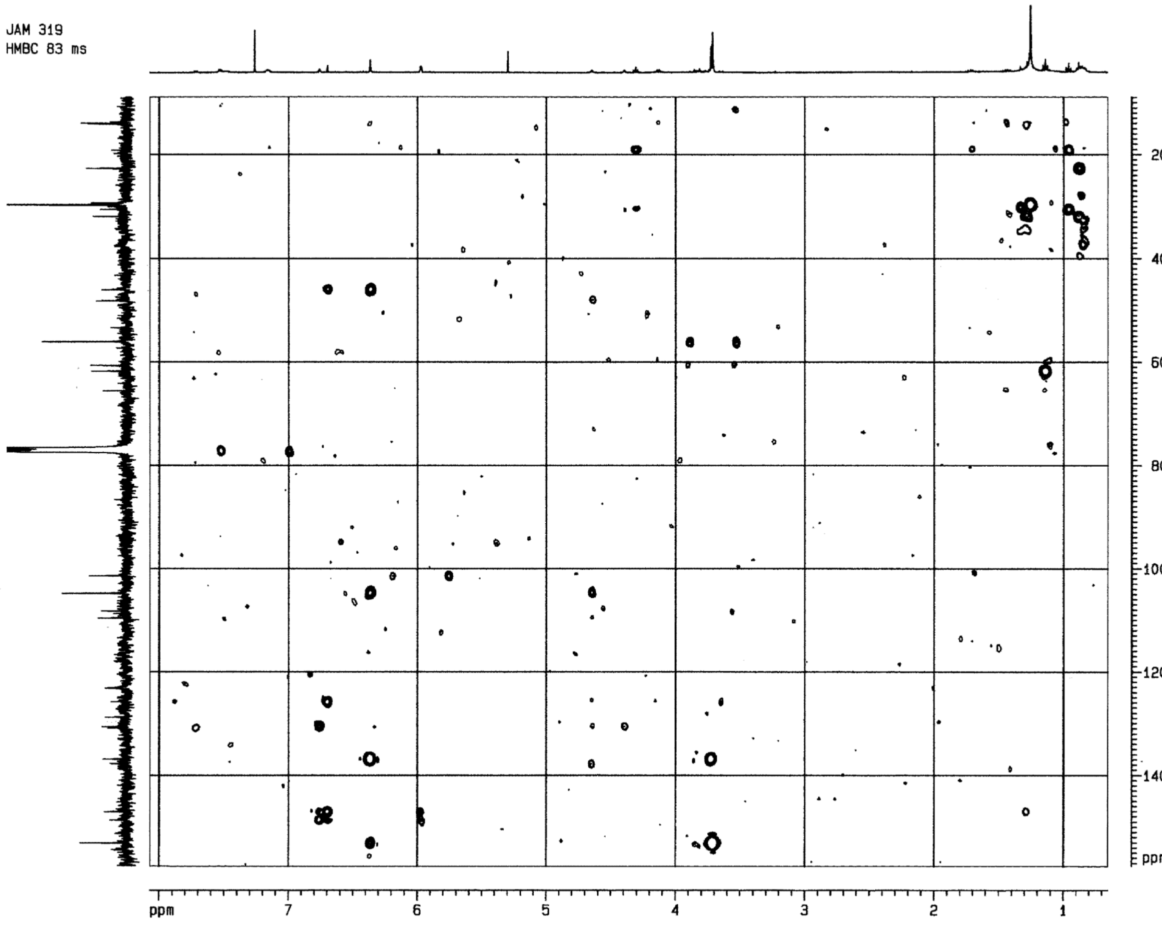

Figure S19. HMQC and HMBC for compound **12**

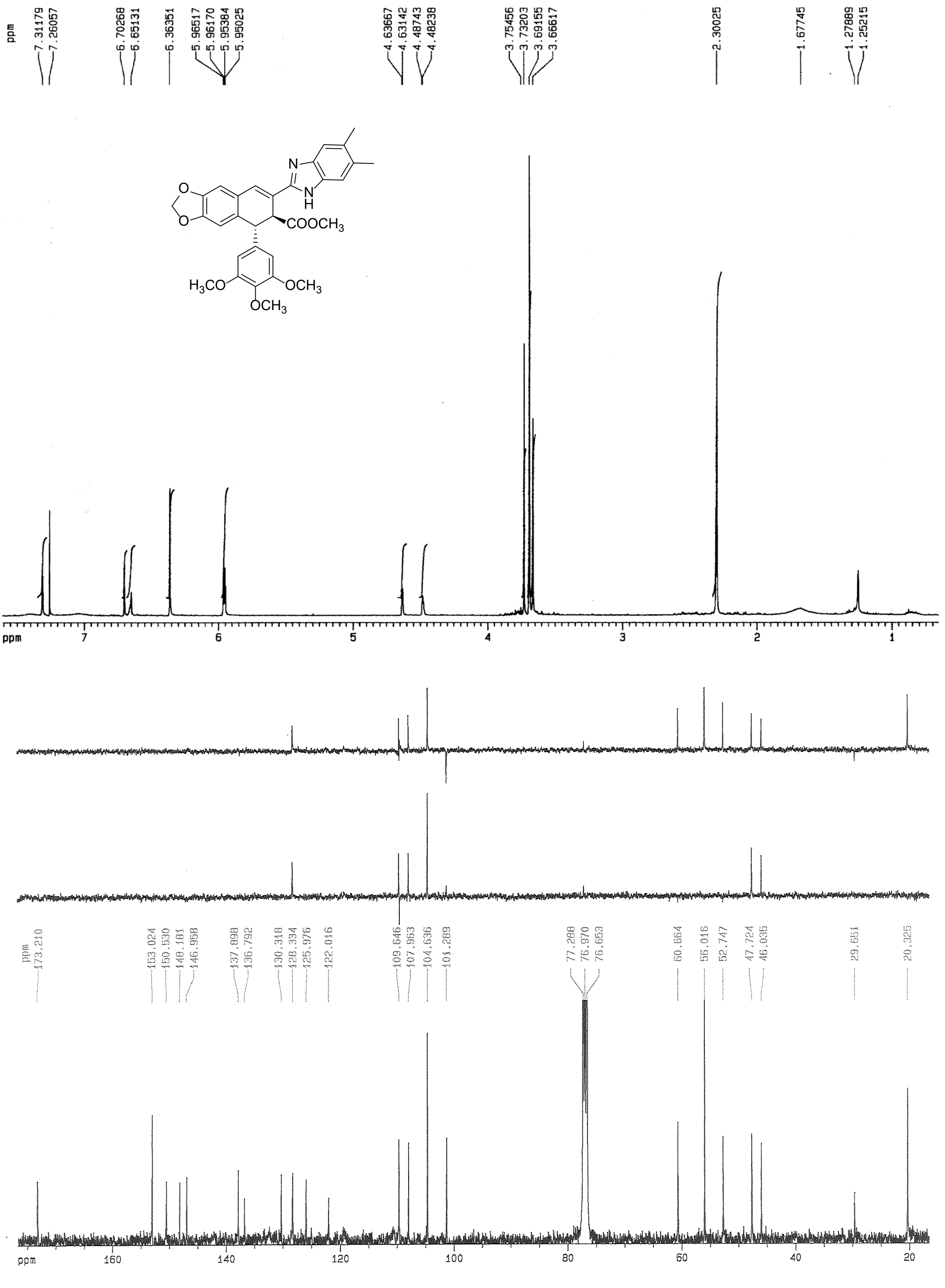

Figure S20. ^1H and ^{13}C for compound **13**

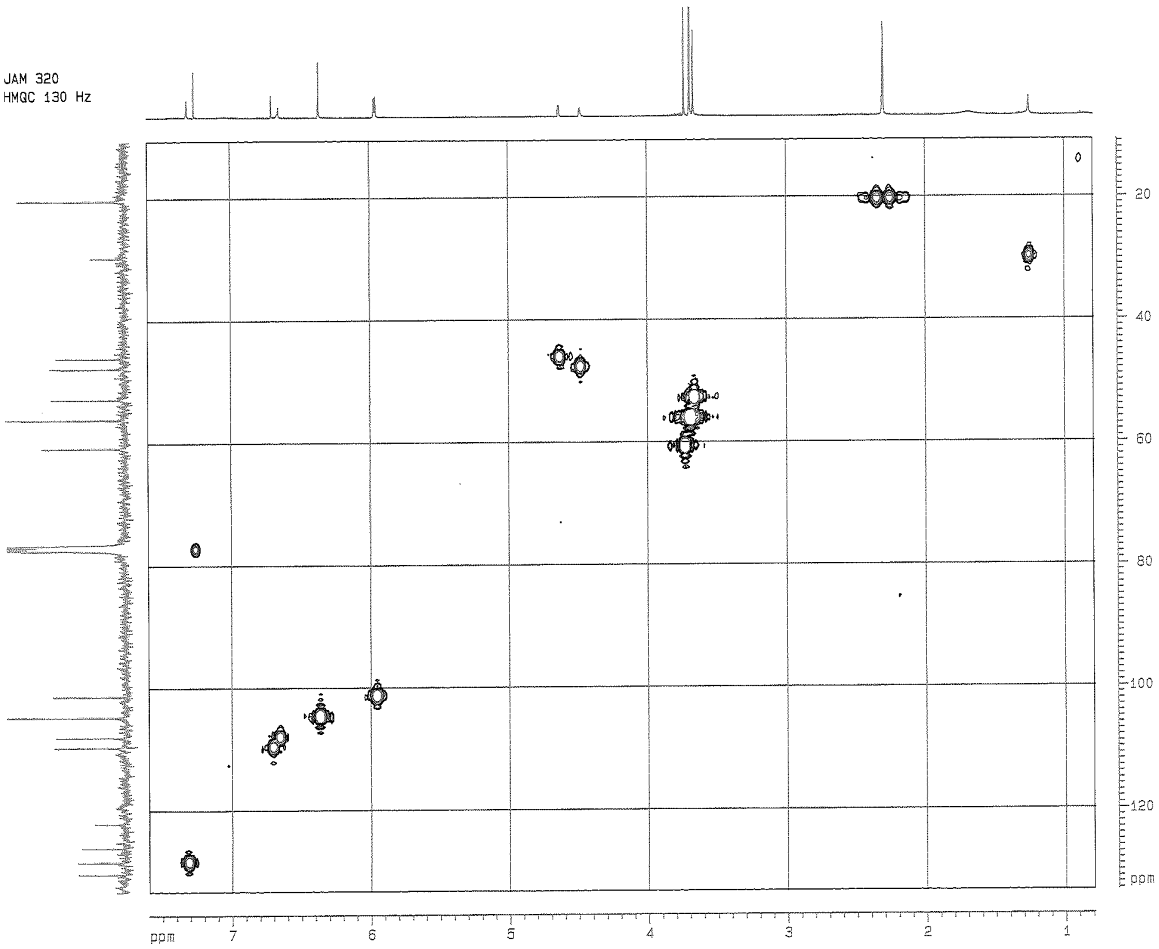

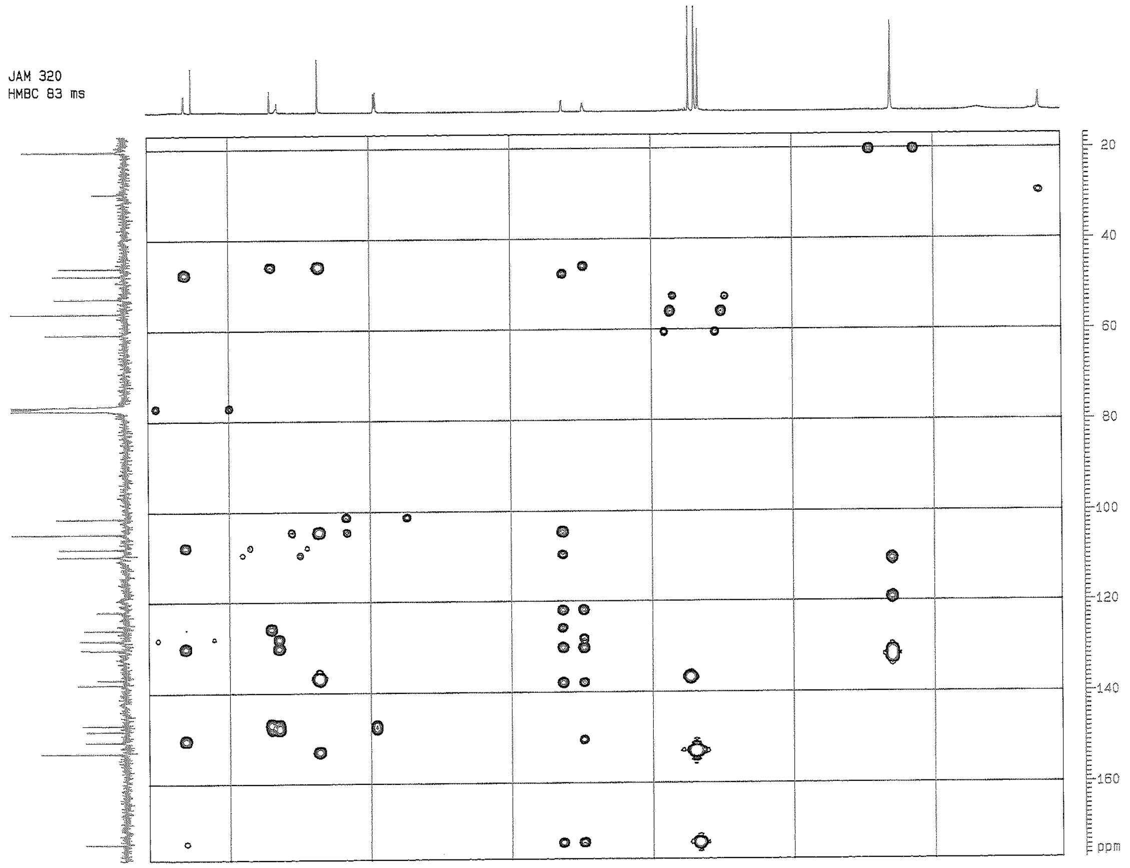

Figure S21. HMQC and HMBC for compound **13**

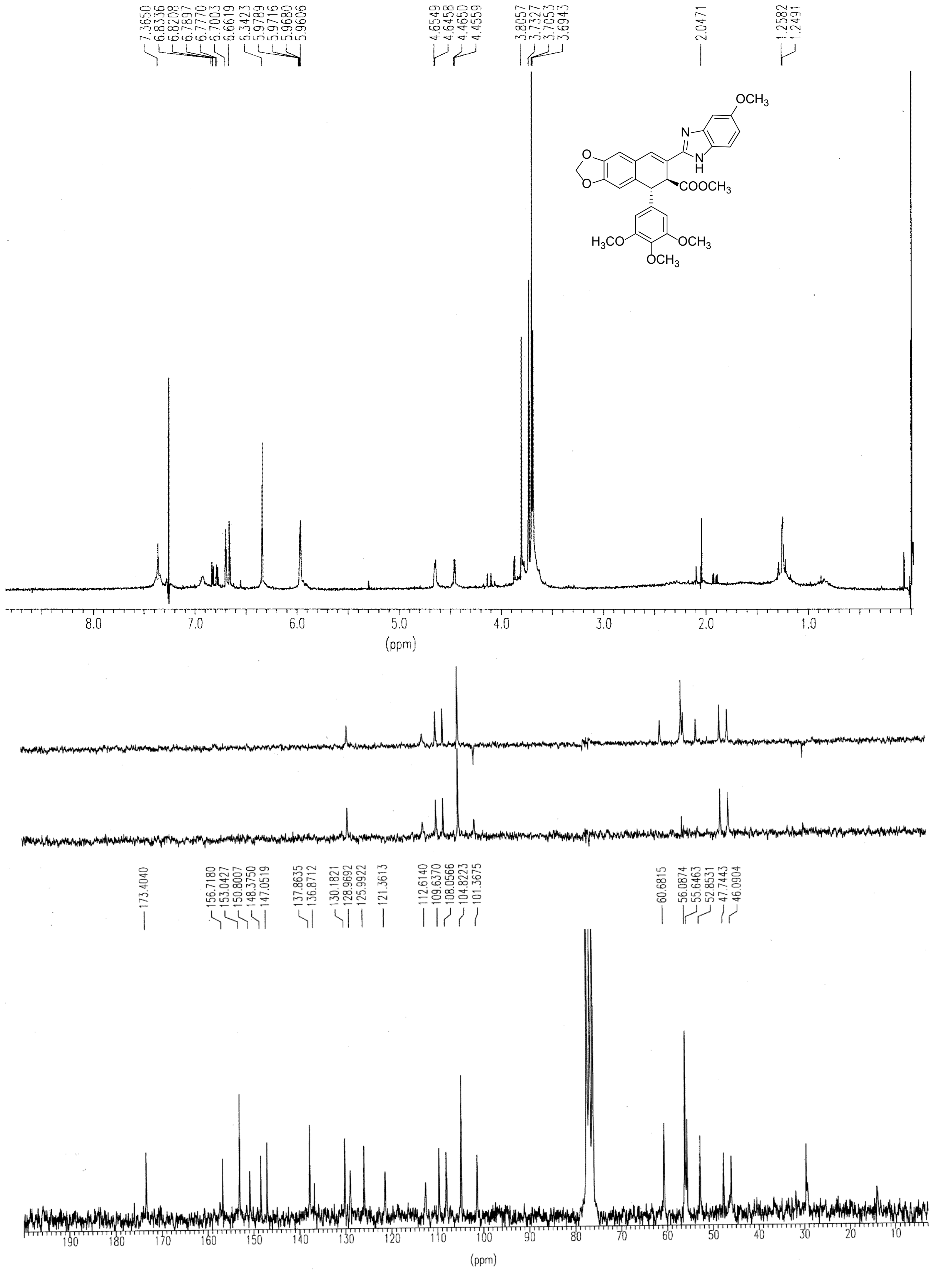

Figure S22. ^{1}H and ^{13}C for compound **14**

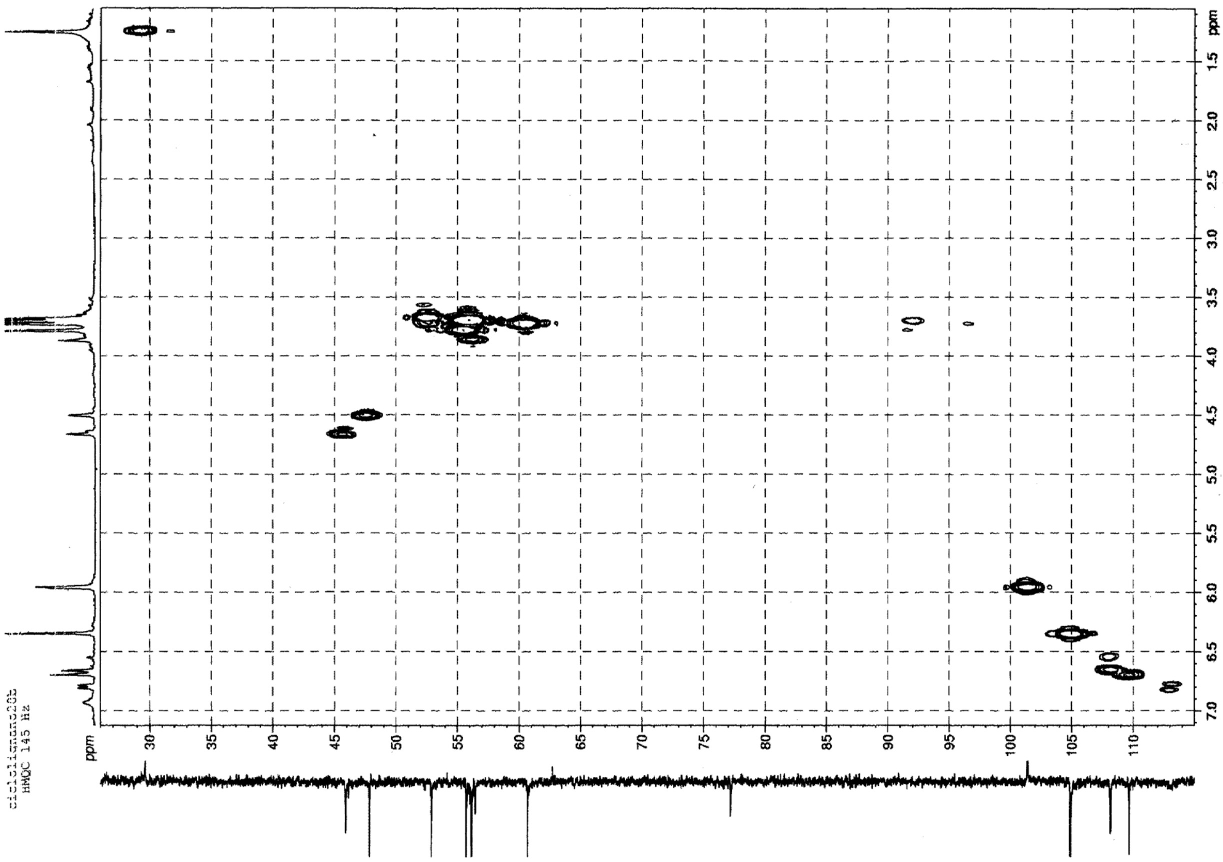

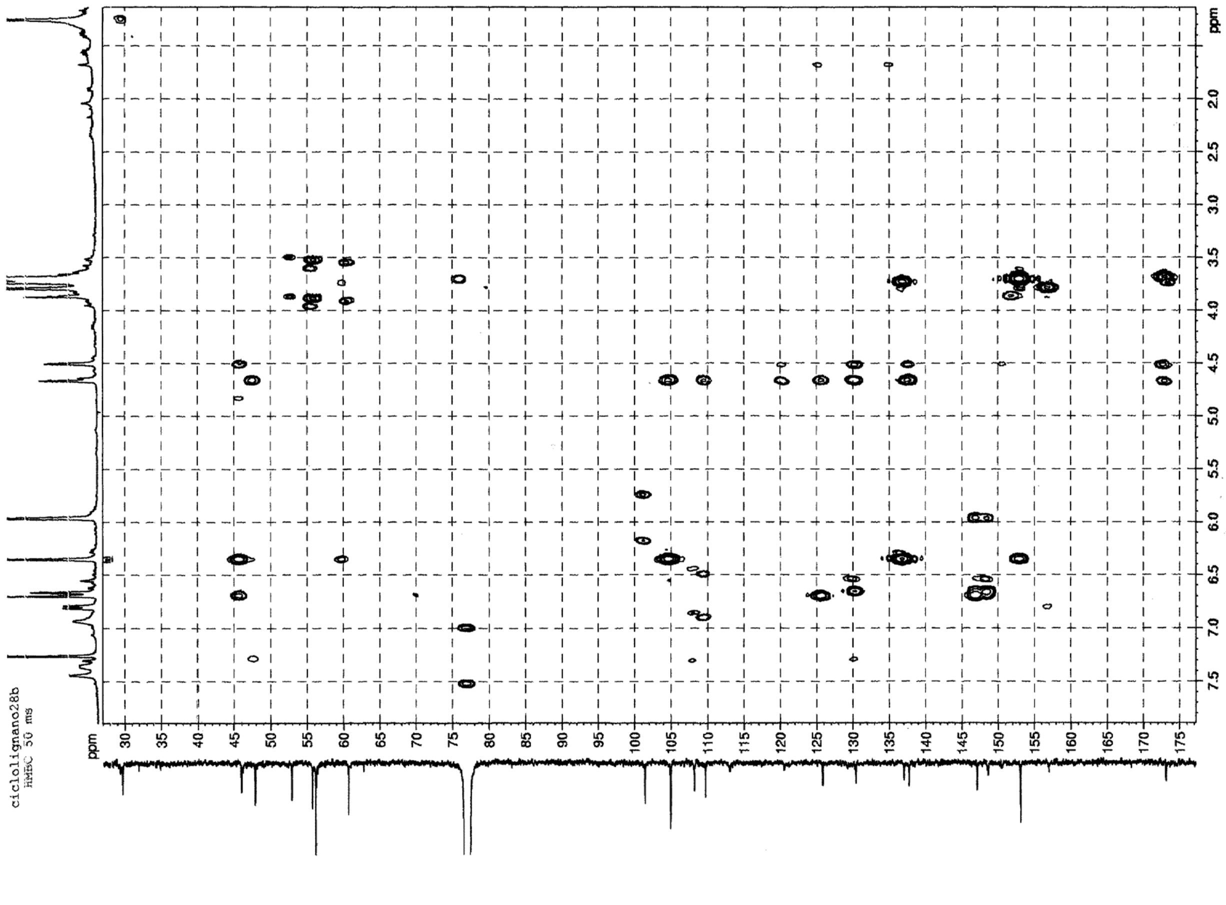

Figure S23. HMQC and HMBC for compound **14**

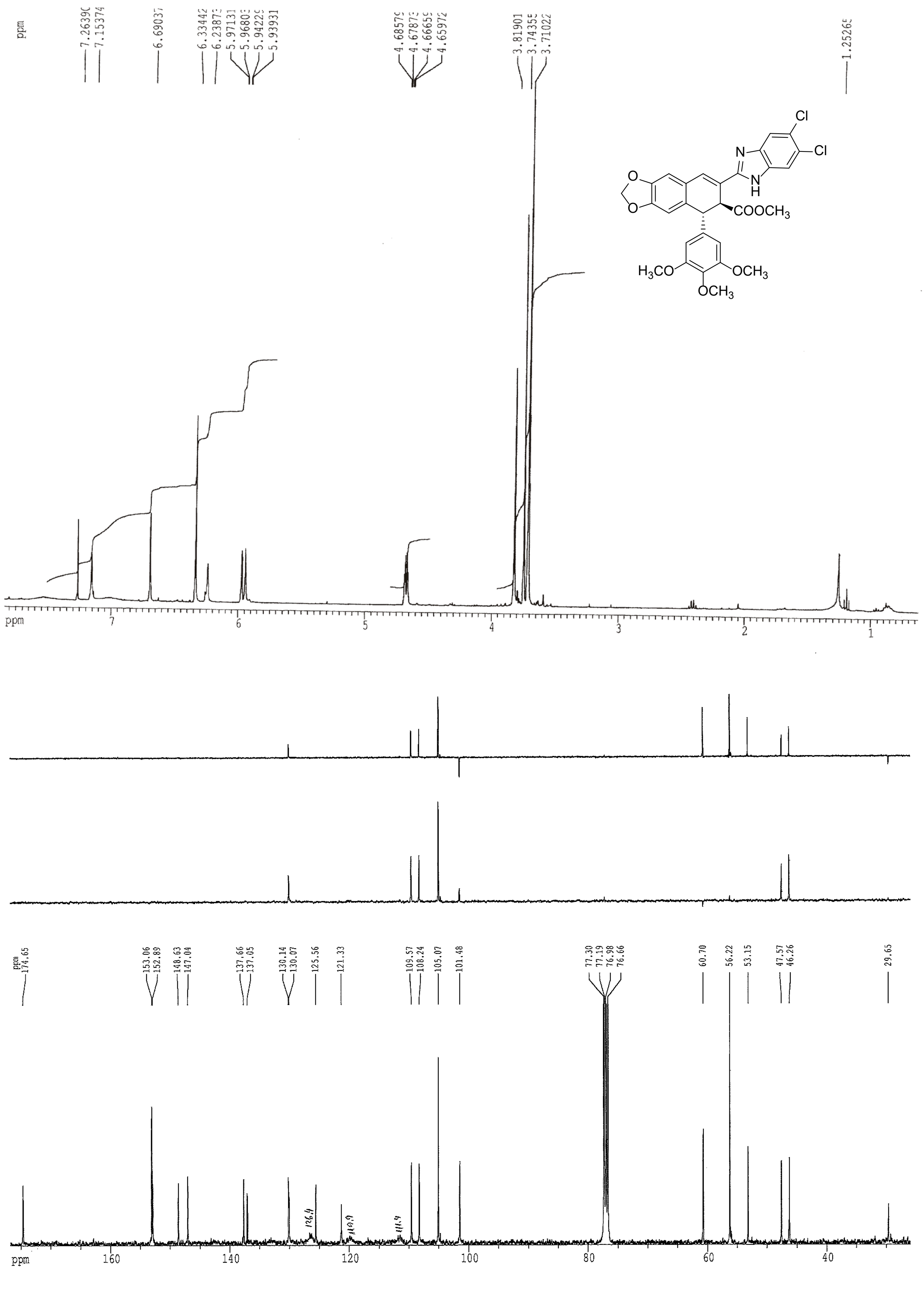

Figure S24. ^{1}H and ^{13}C for compound **15**

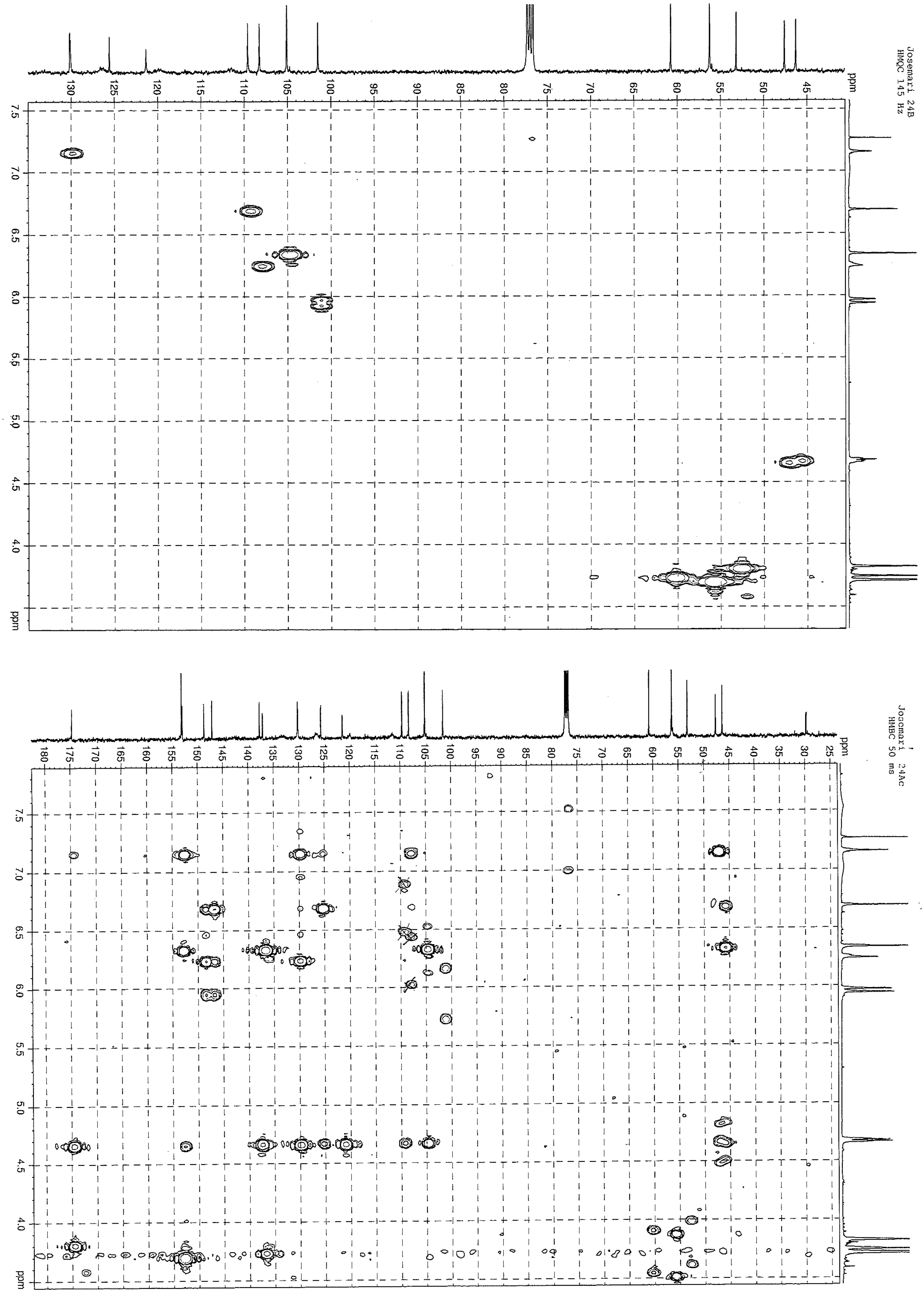

Figure S25. HMQC and HMBC for compound **15**

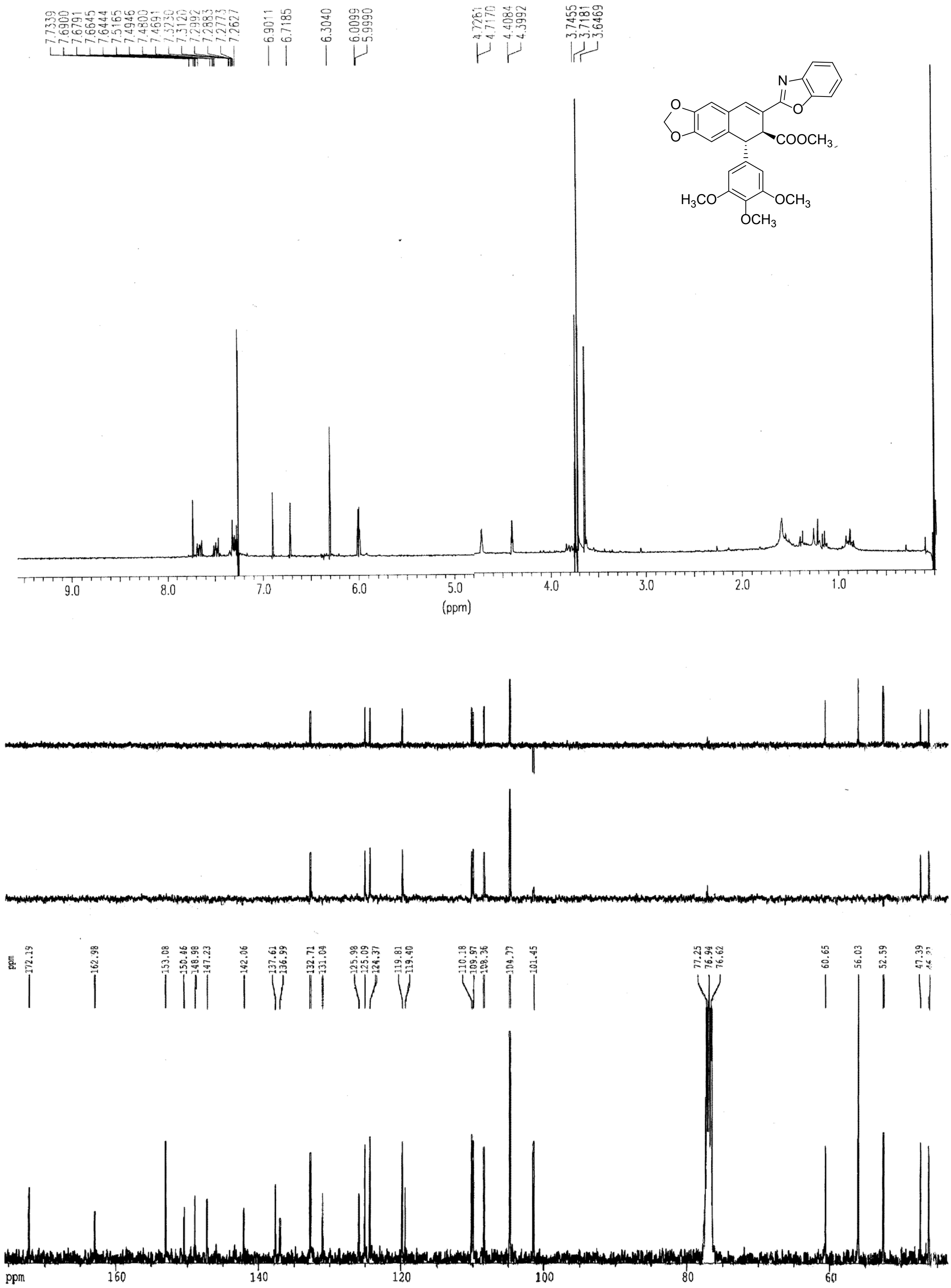

Figure S26. ^{1}H and ^{13}C for compound **16**

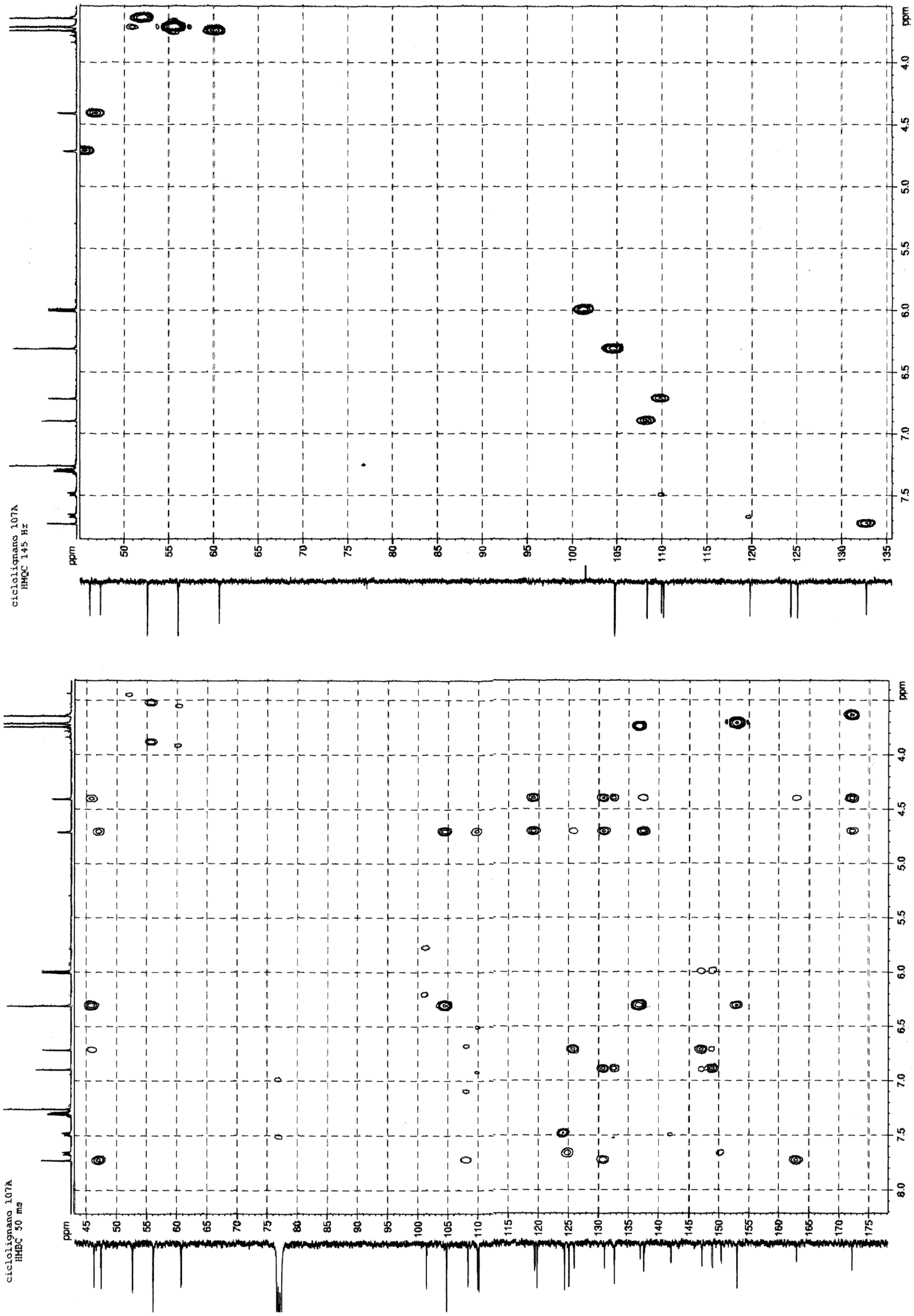

Figure S27. HMQC and HMBC for compound **16**

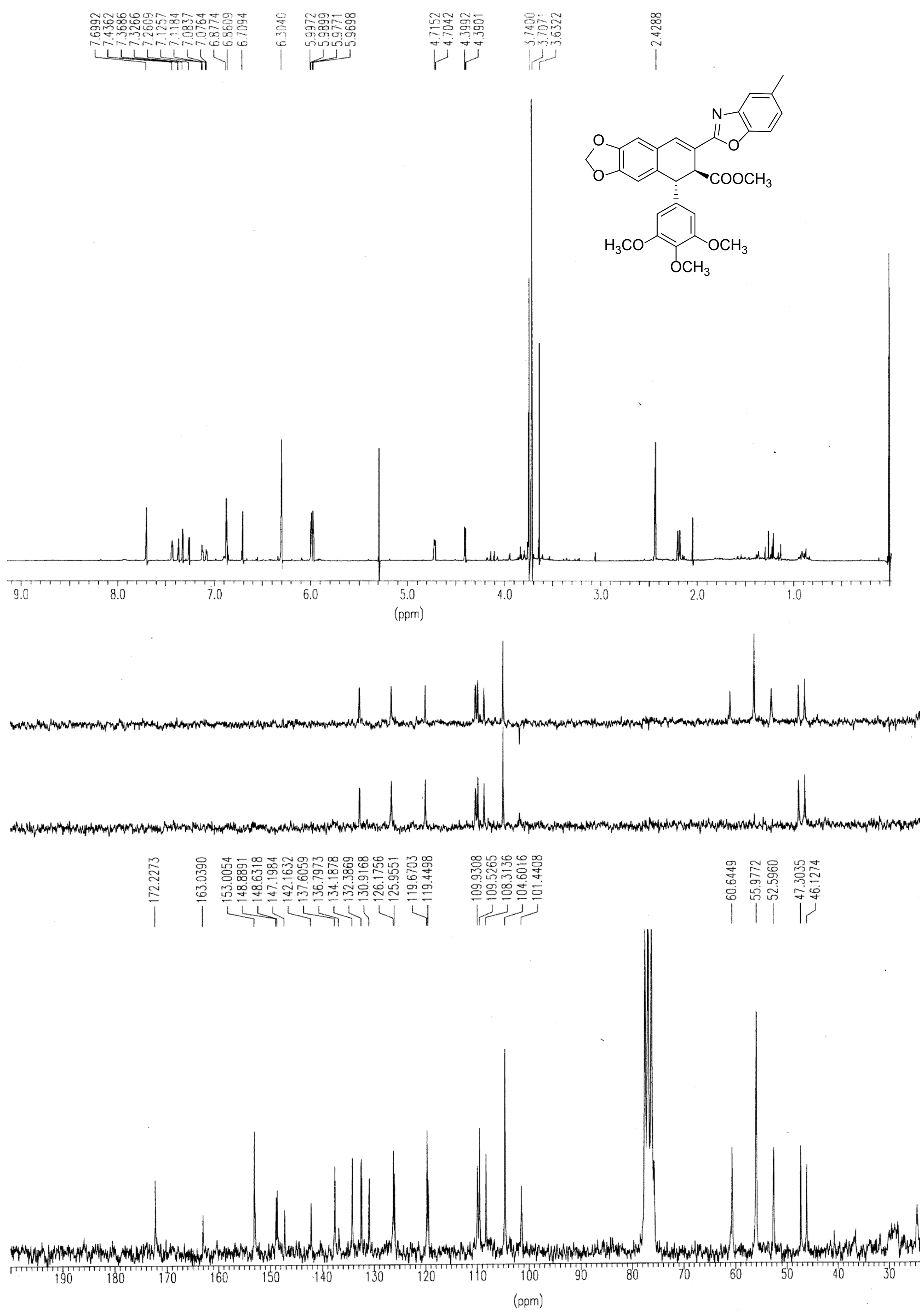

Figure S28. ^{1}H and ^{13}C for compound **17**

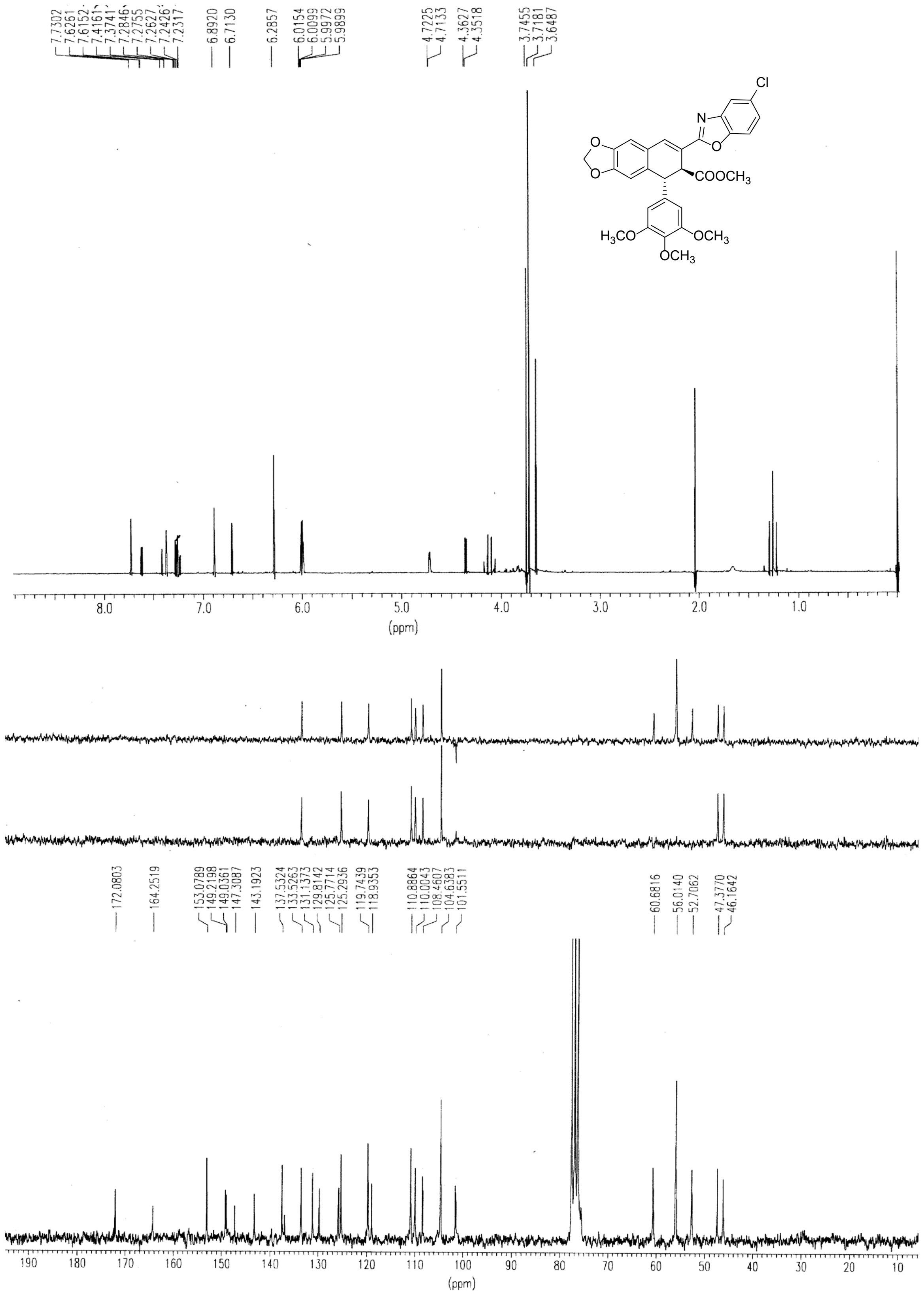

Figure S29. ^{1}H and ^{13}C for compound **18**

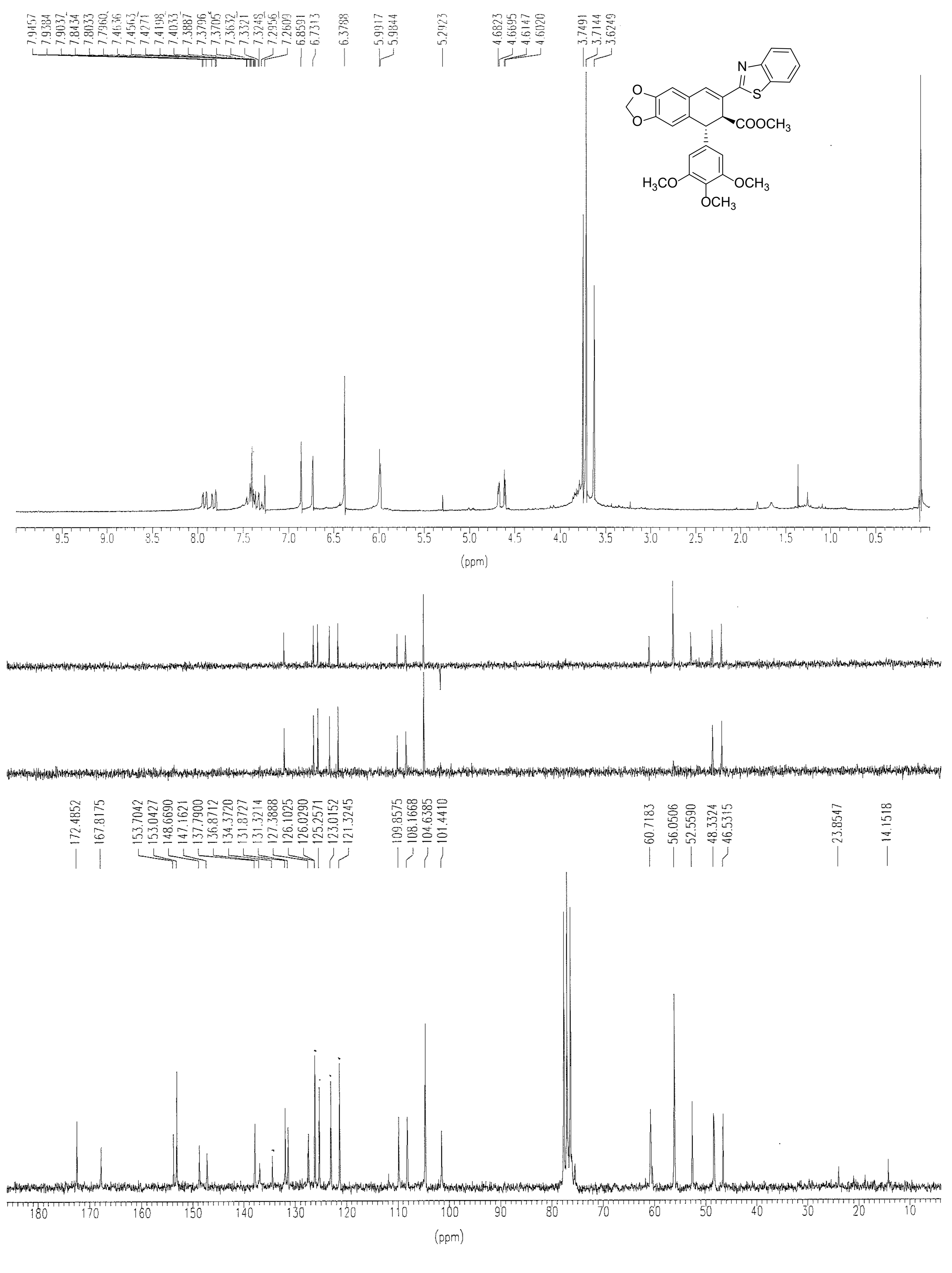

Figure S30. ^{1}H and ^{13}C for compound **19**

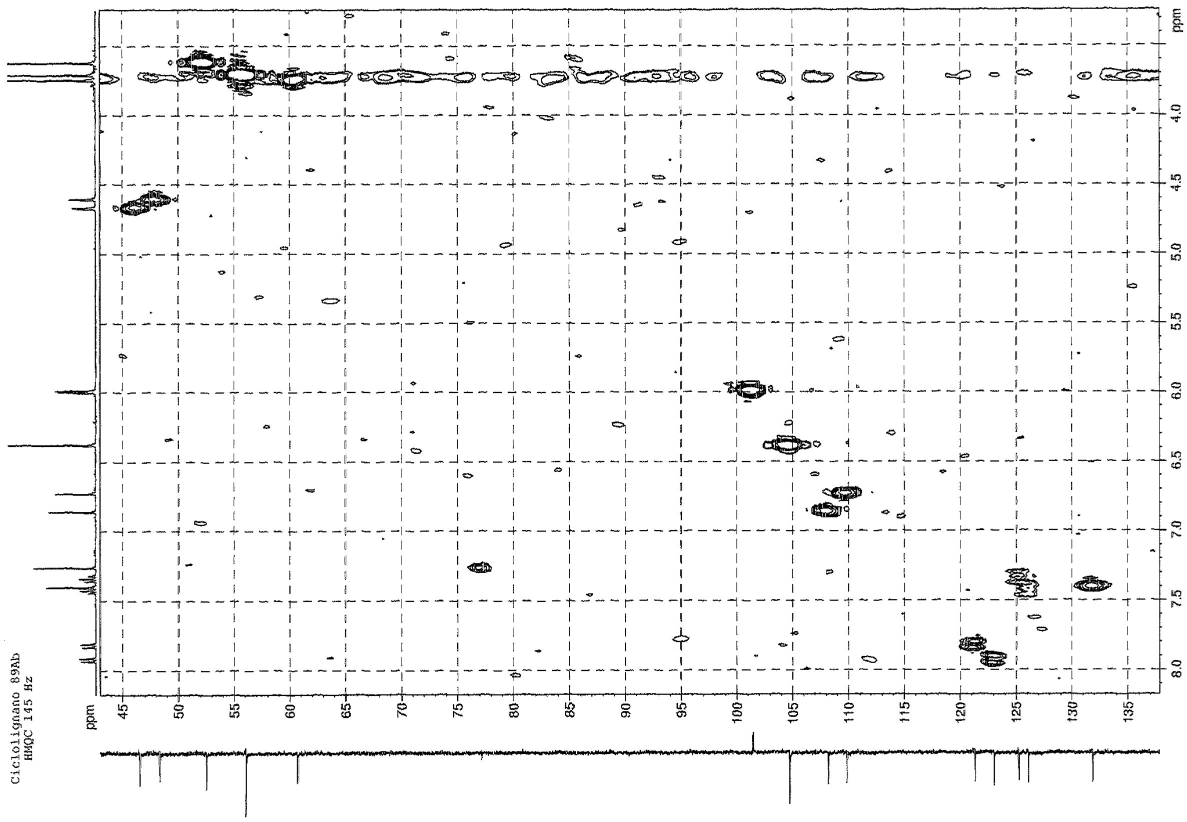

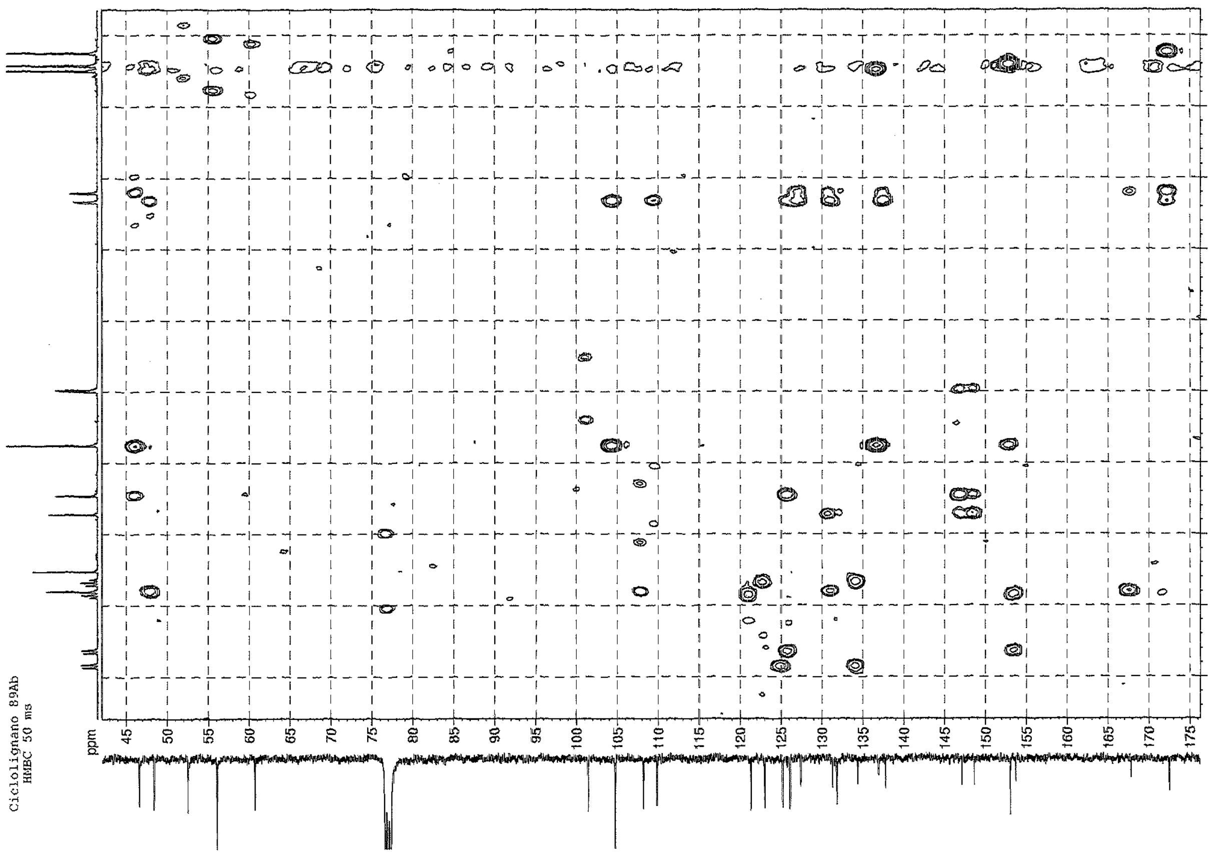

Figure S31. HMQC and HMBC for compound **19**

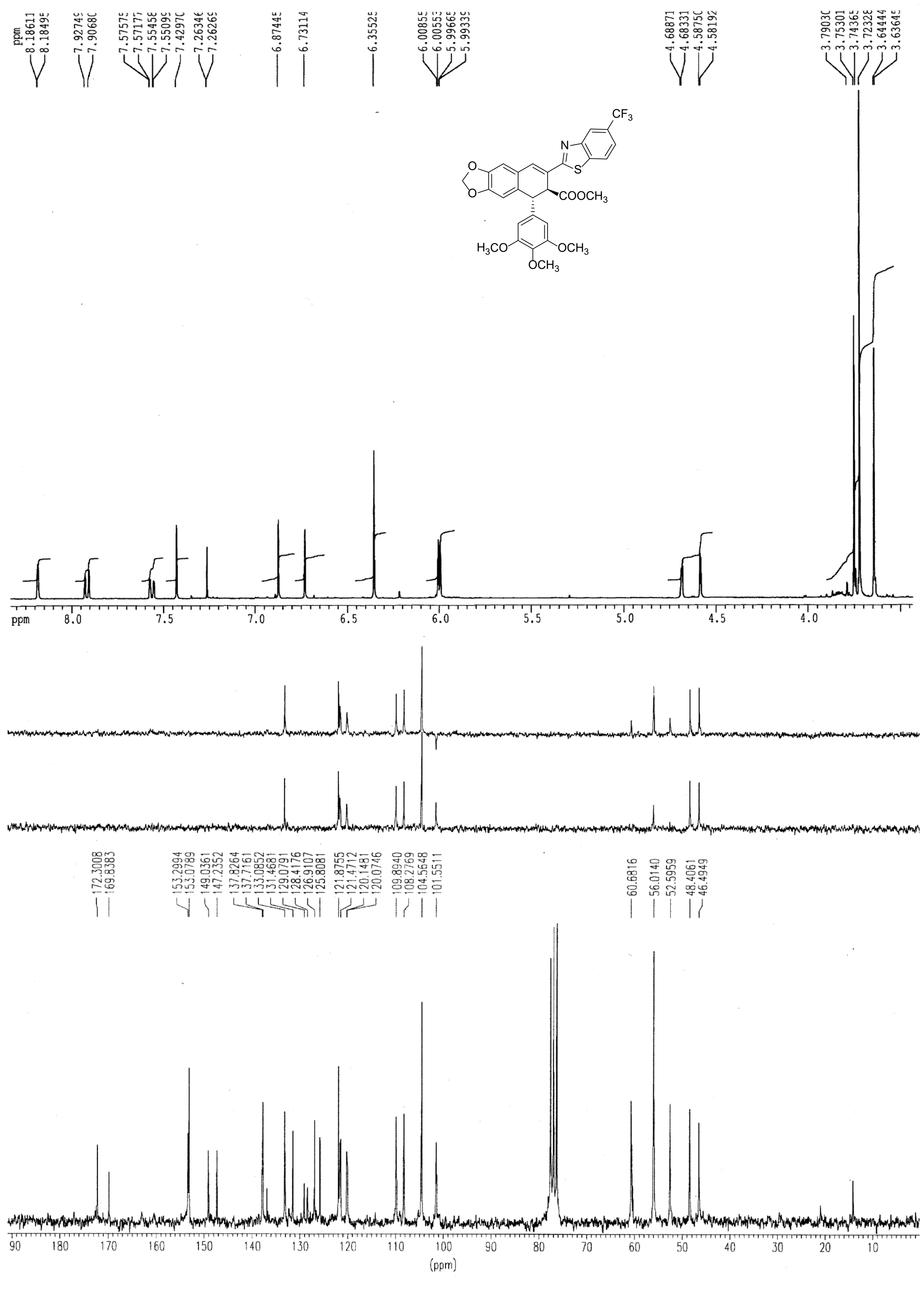

Figure S32. ^{1}H and ^{13}C for compound **20**

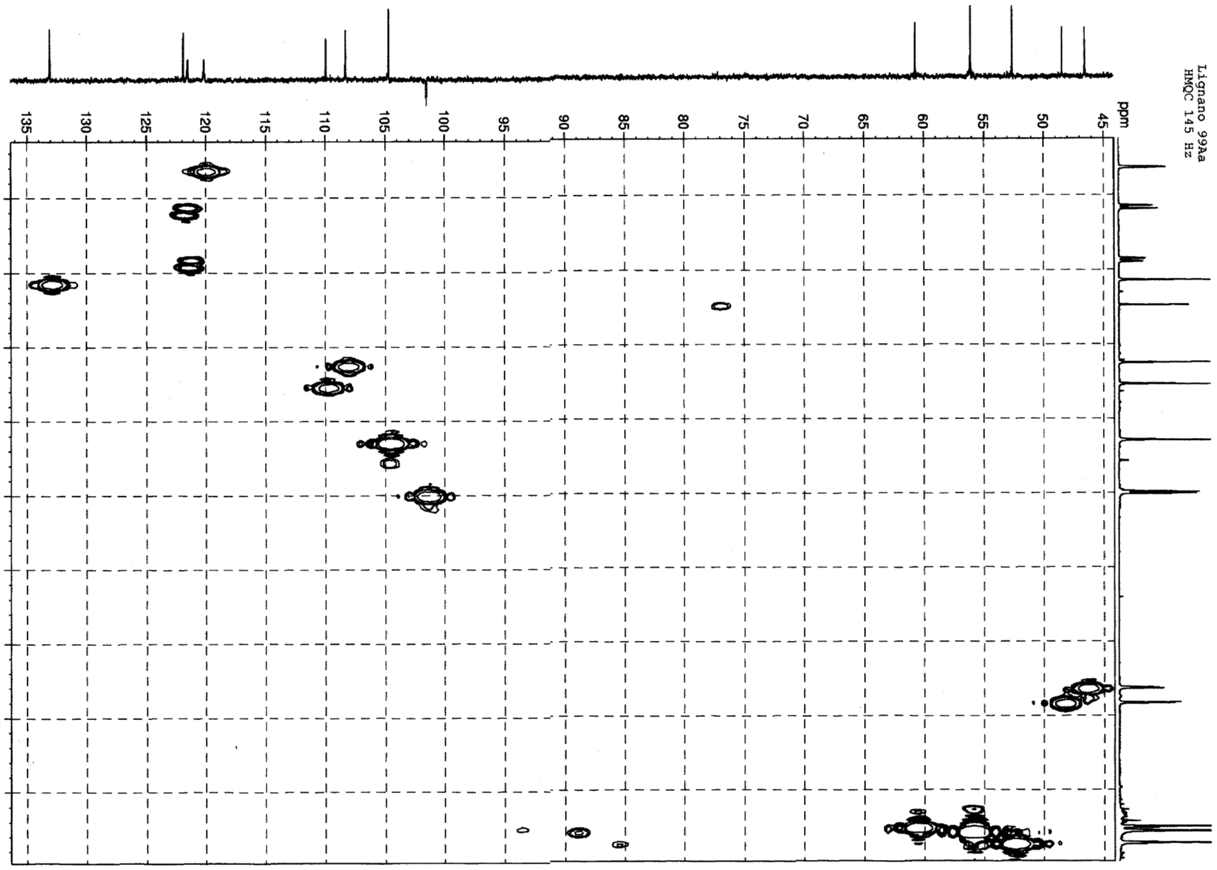

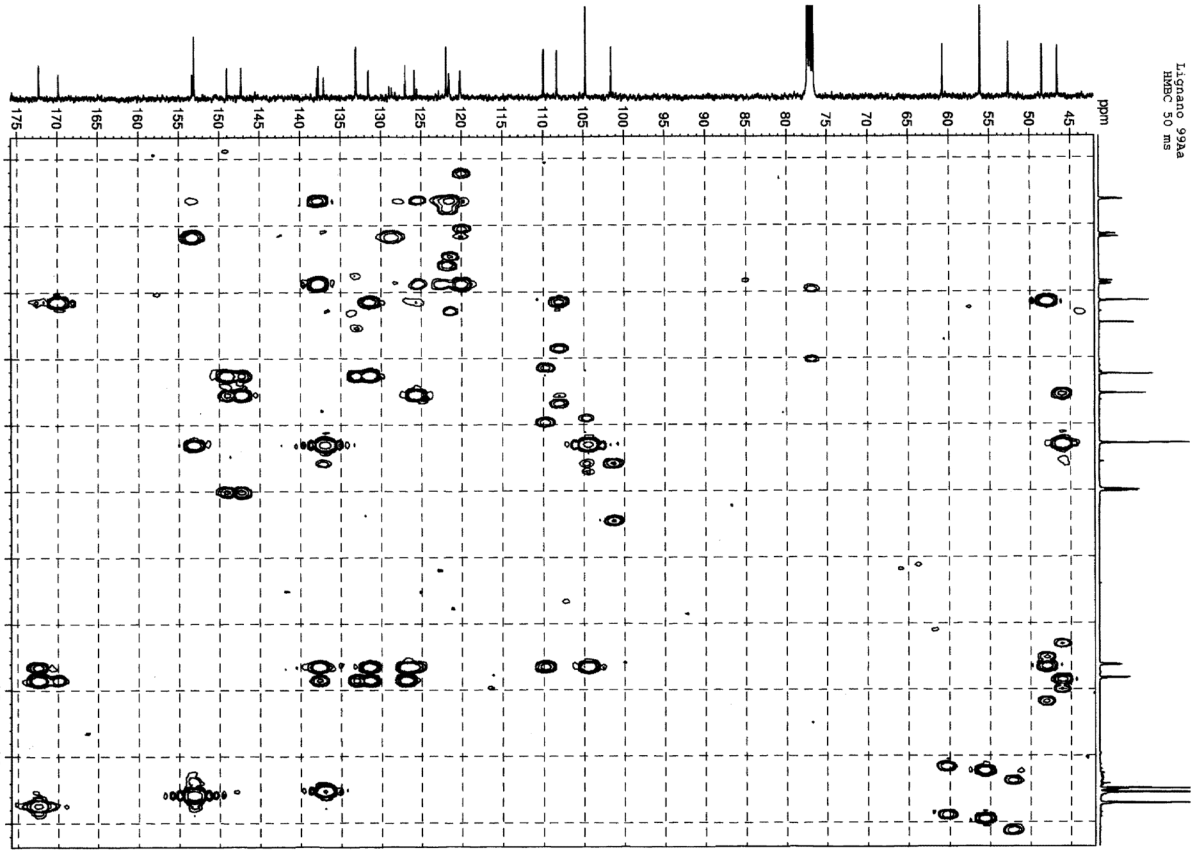

Figure S33. HMQC and HMBC for compound **20**

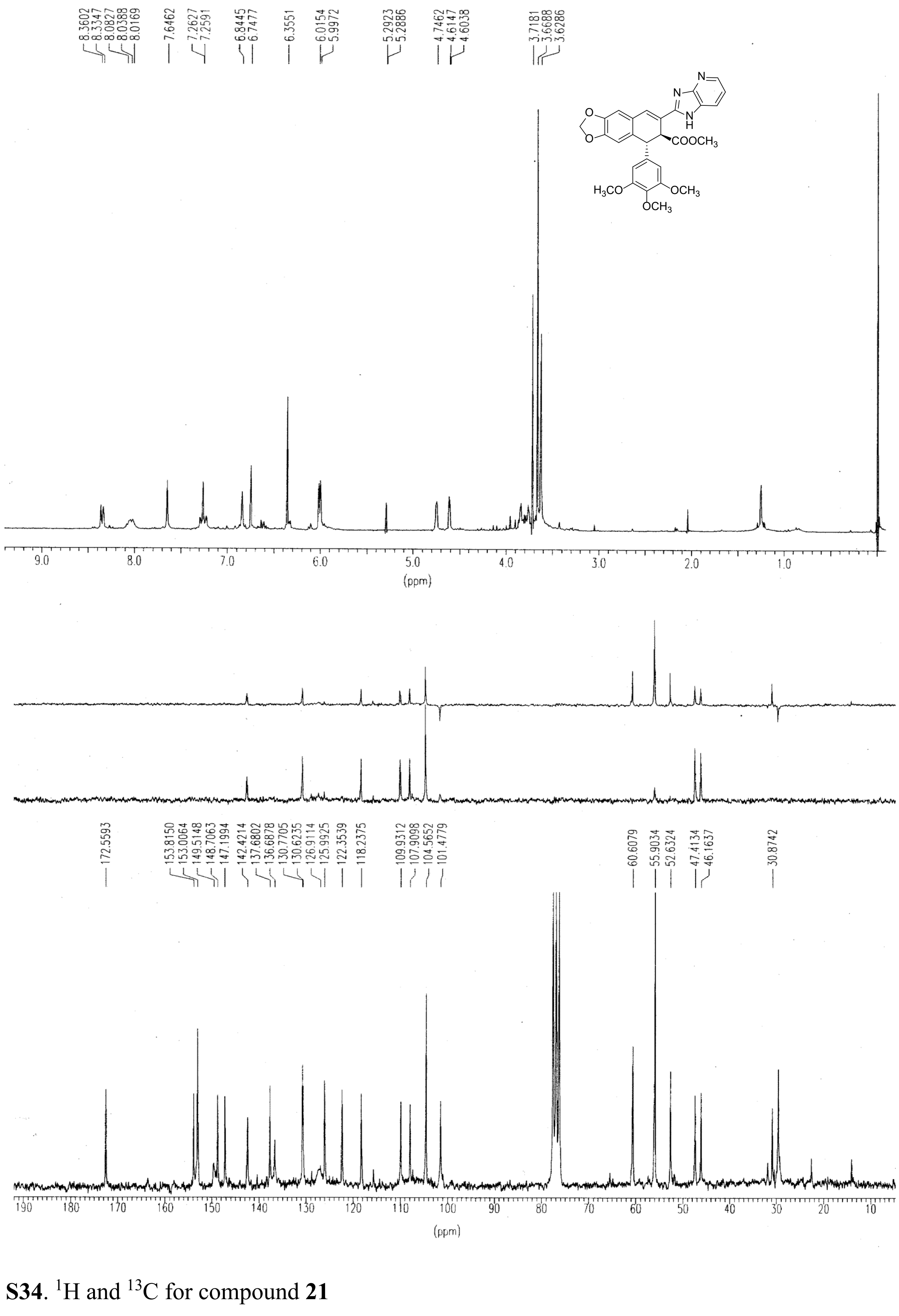

Figure S34. ^{1}H and ^{13}C for compound **21**

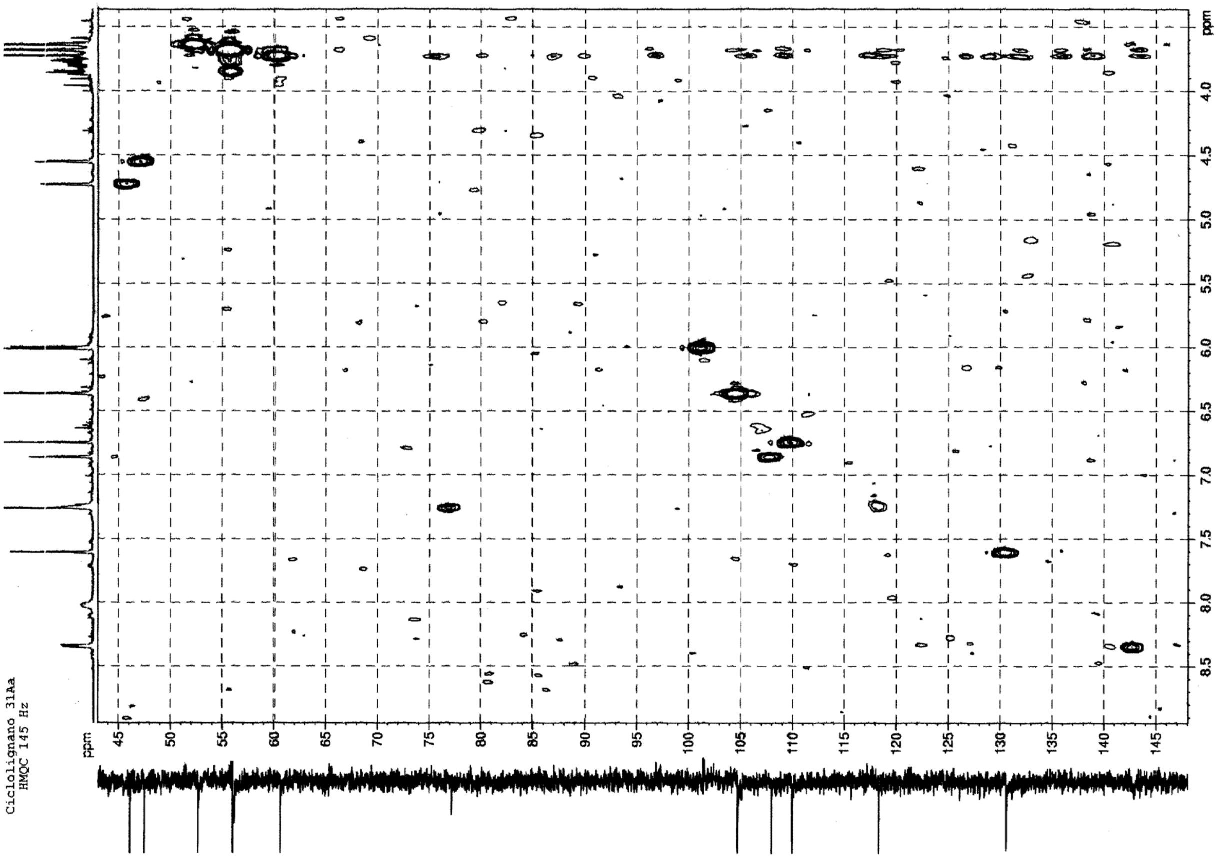

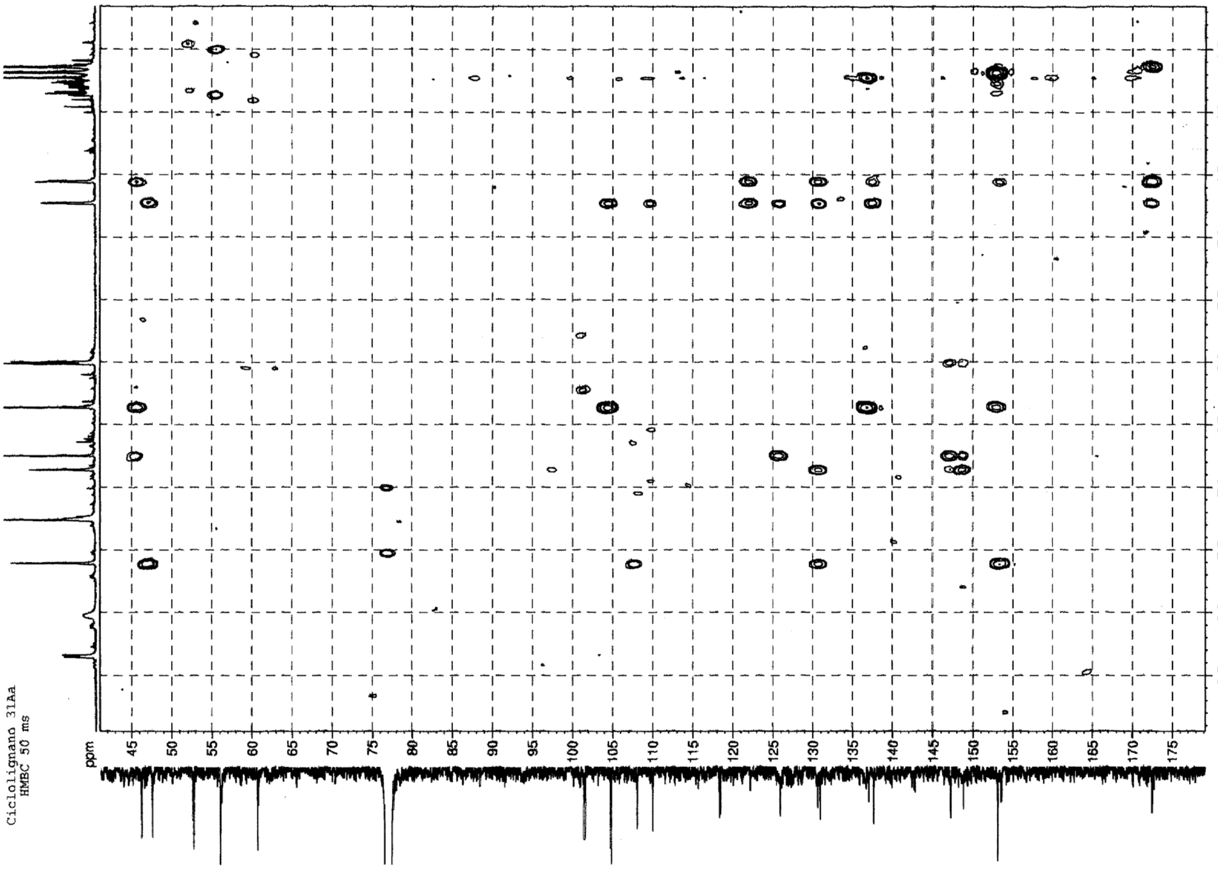

Figure S35. HMQC and HMBC for compound **21**

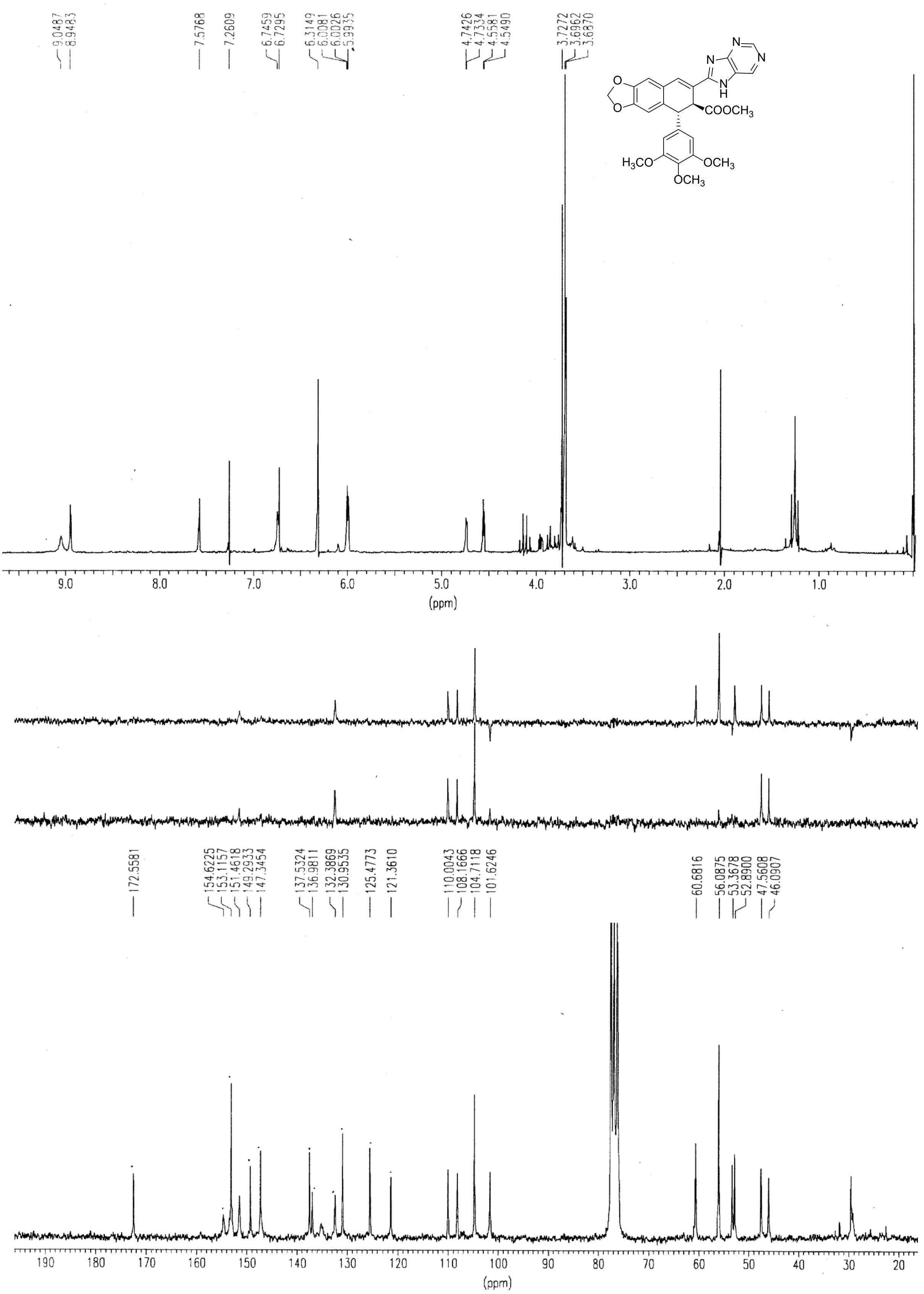

Figure S36. ^{1}H and ^{13}C for compound **22**

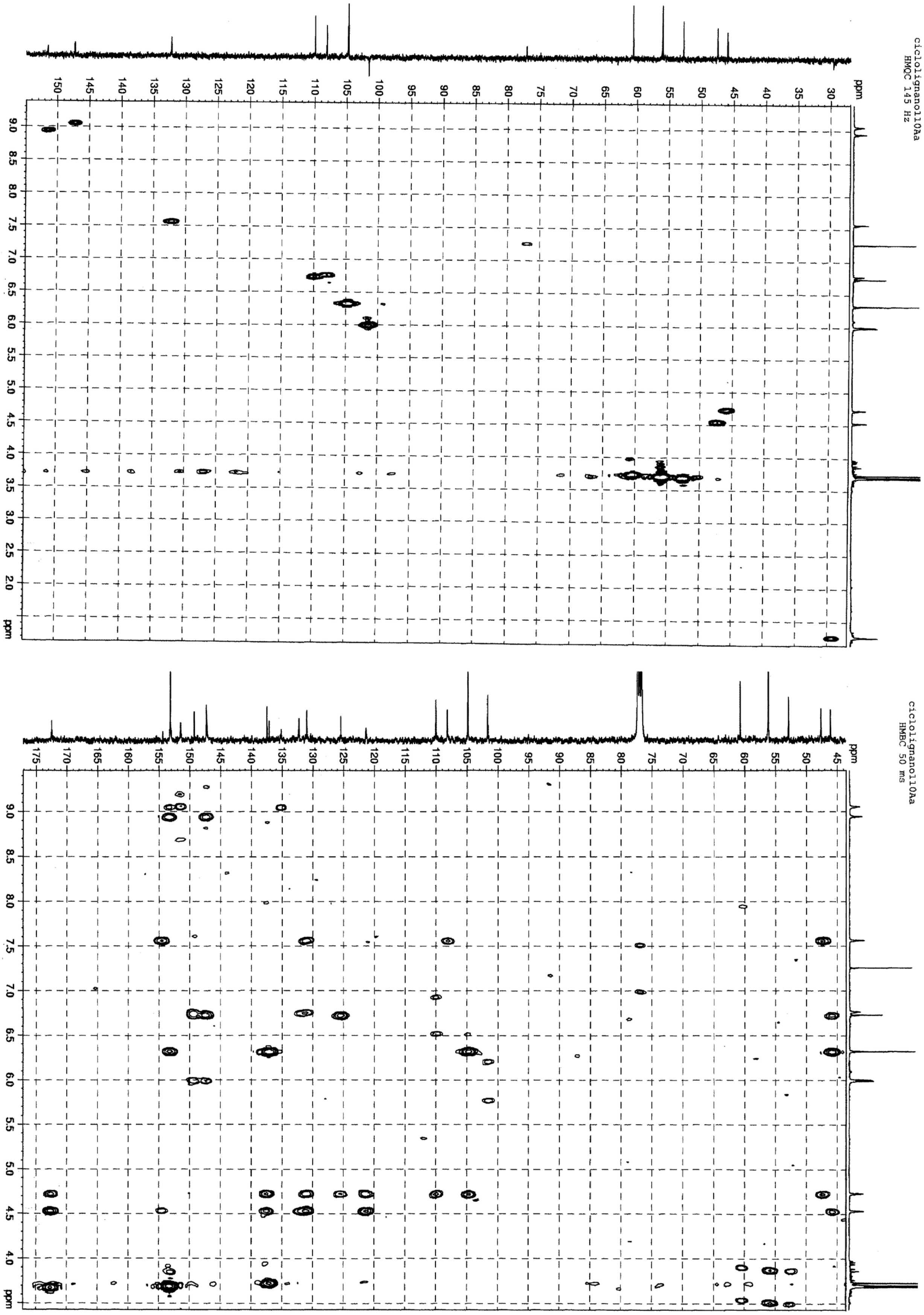

Figure S37. HMQC and HMBC for compound **22**

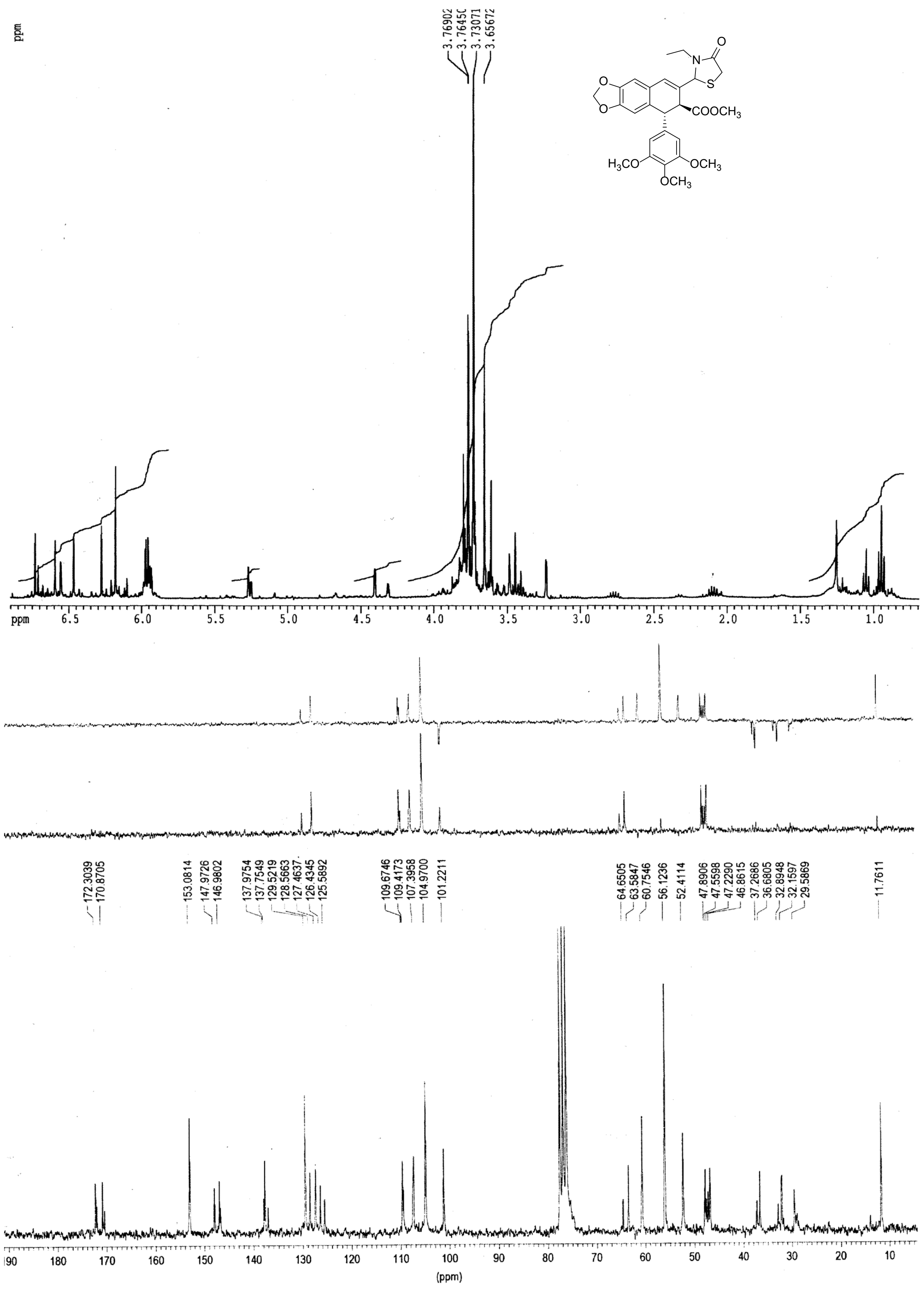

Figure S38. ^{1}H and ^{13}C for compound **28**

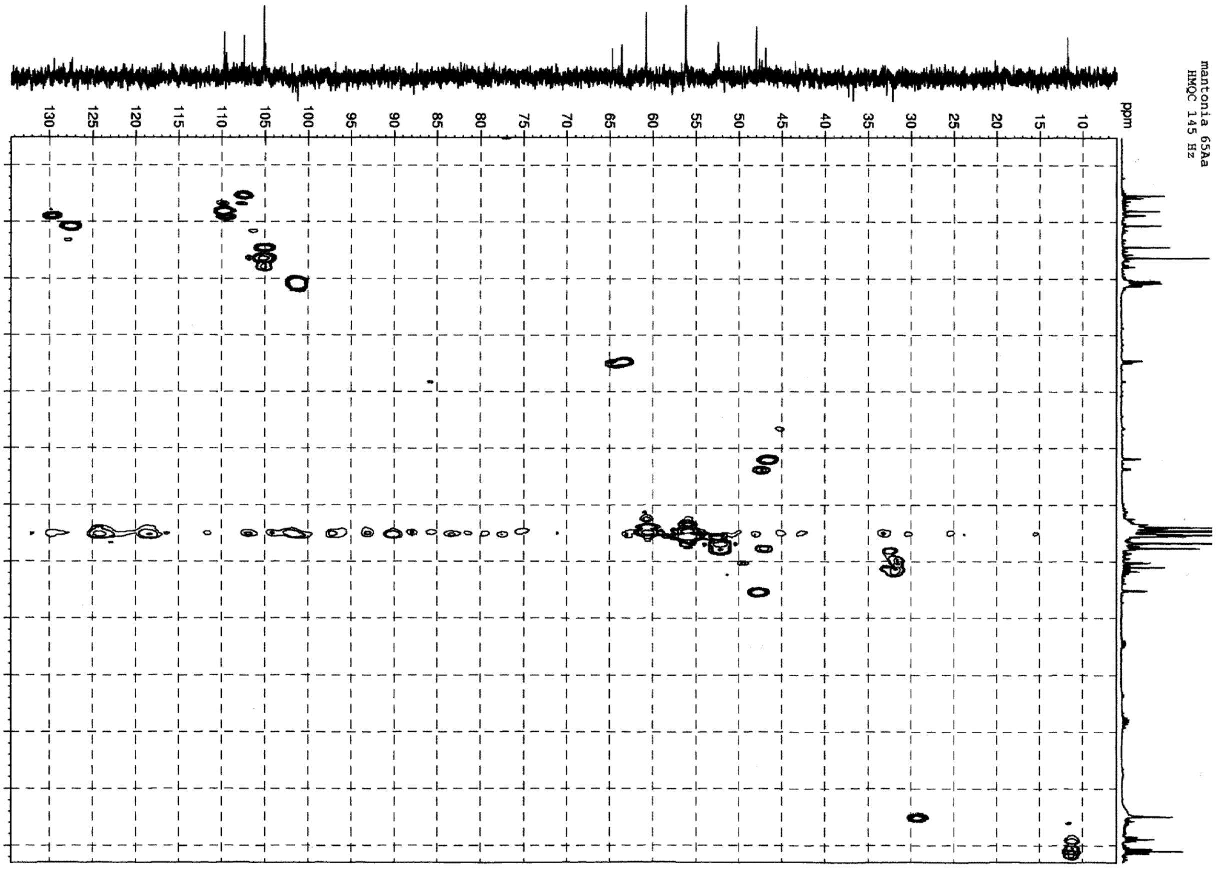

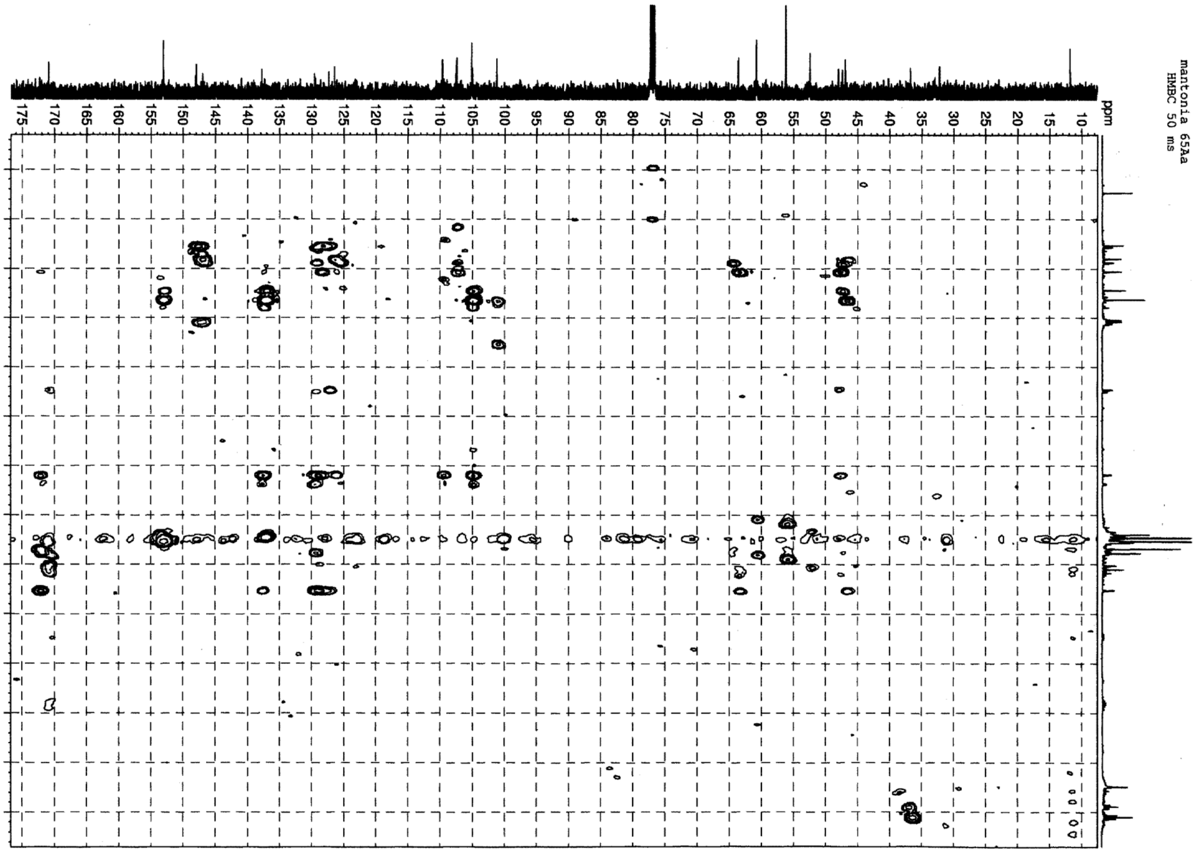

Figure S39. HMQC and HMBC for compound **28**

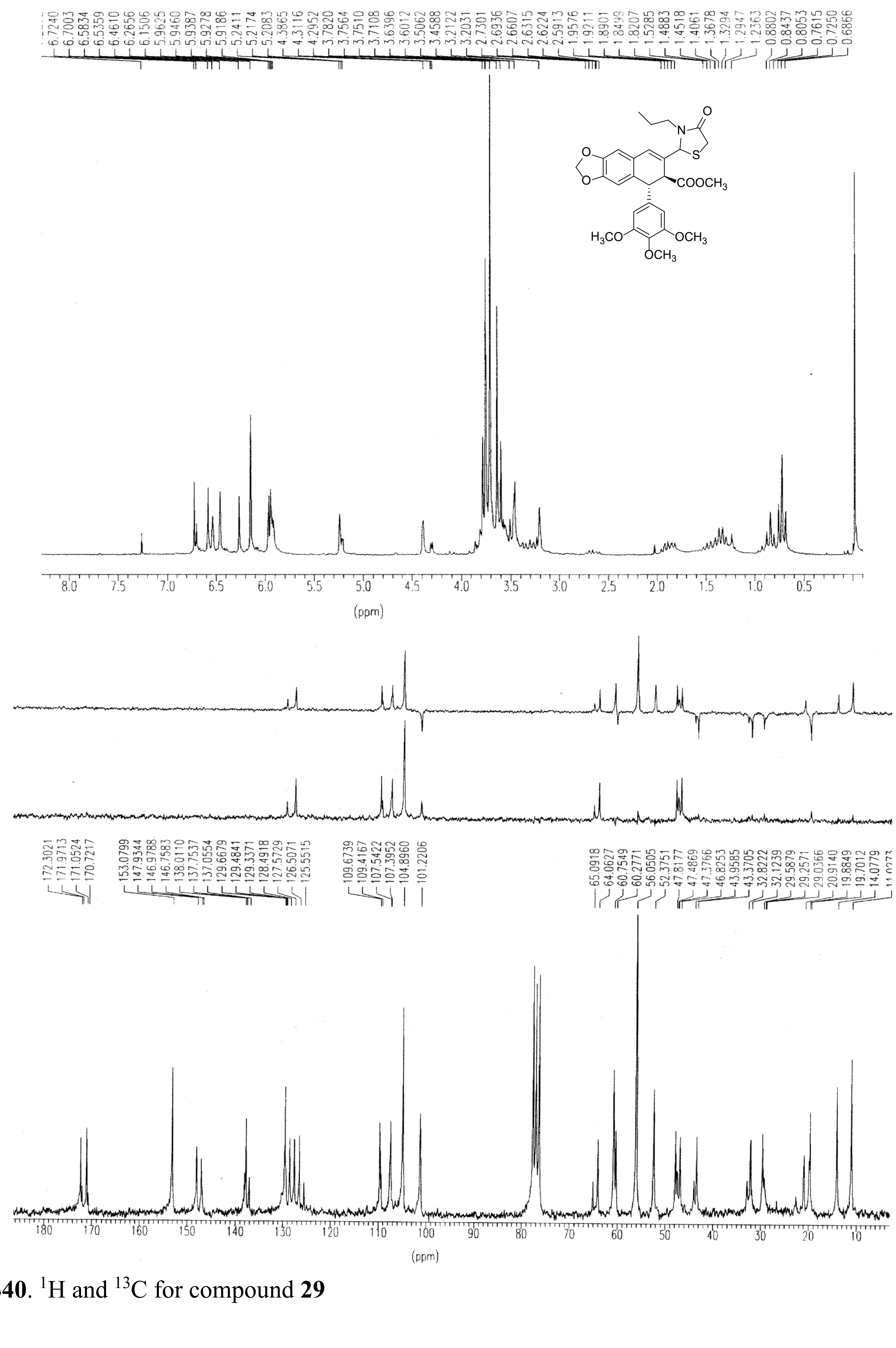

Figure S40. ^{1}H and ^{13}C for compound **29**

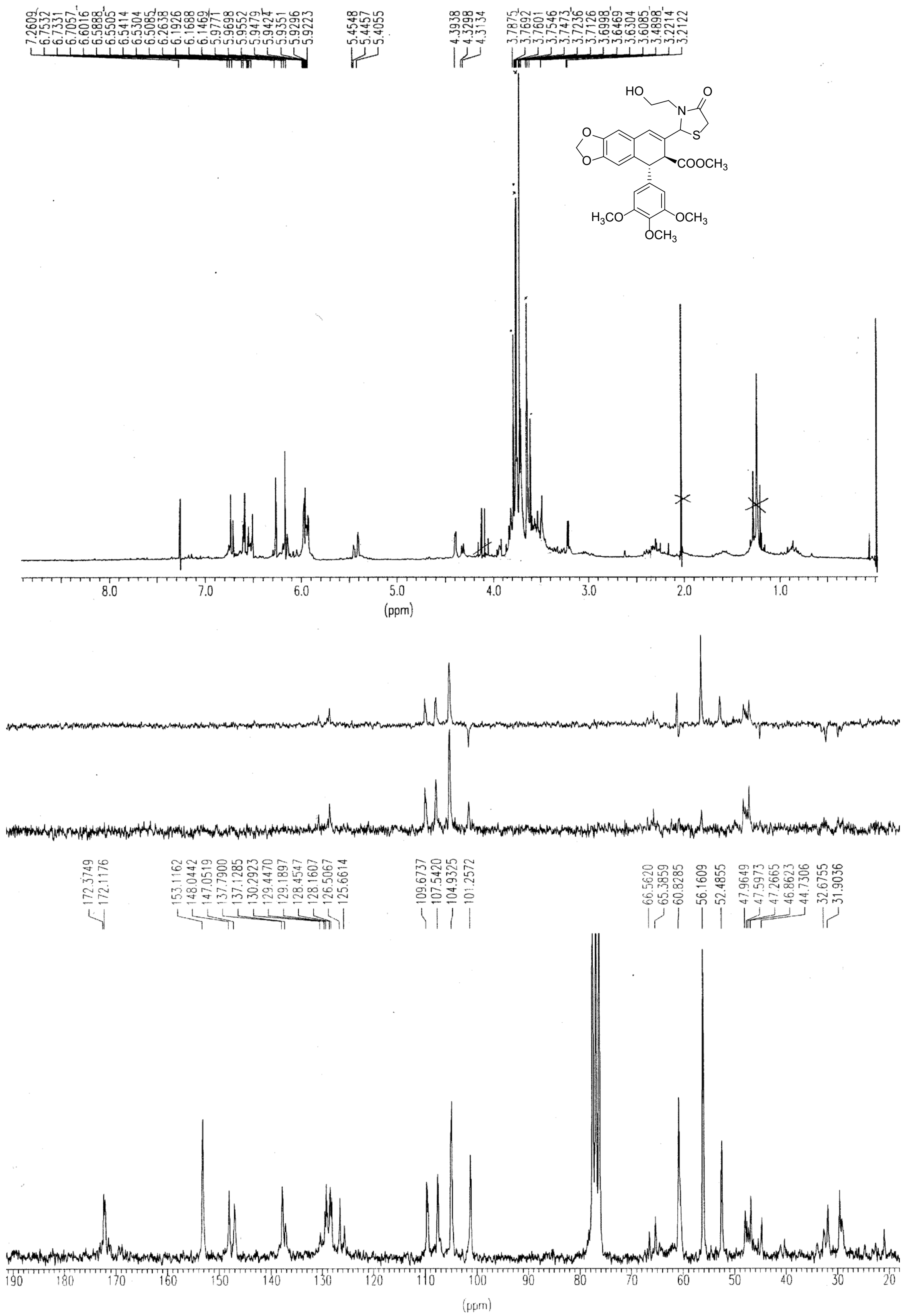

Figure S41. ^{1}H and ^{13}C for compound **30**

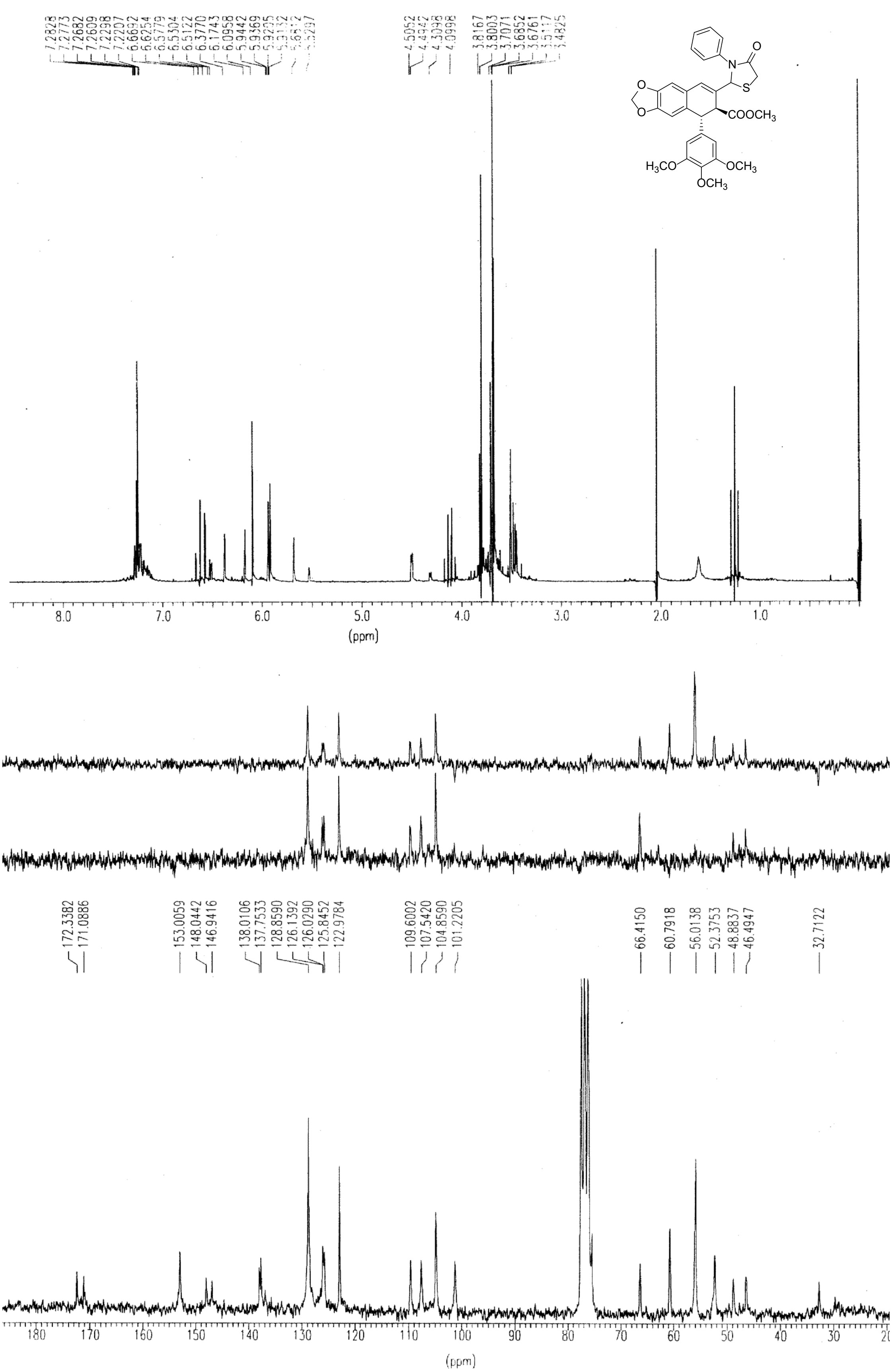

Figure S42. ^{1}H and ^{13}C for compound **31**

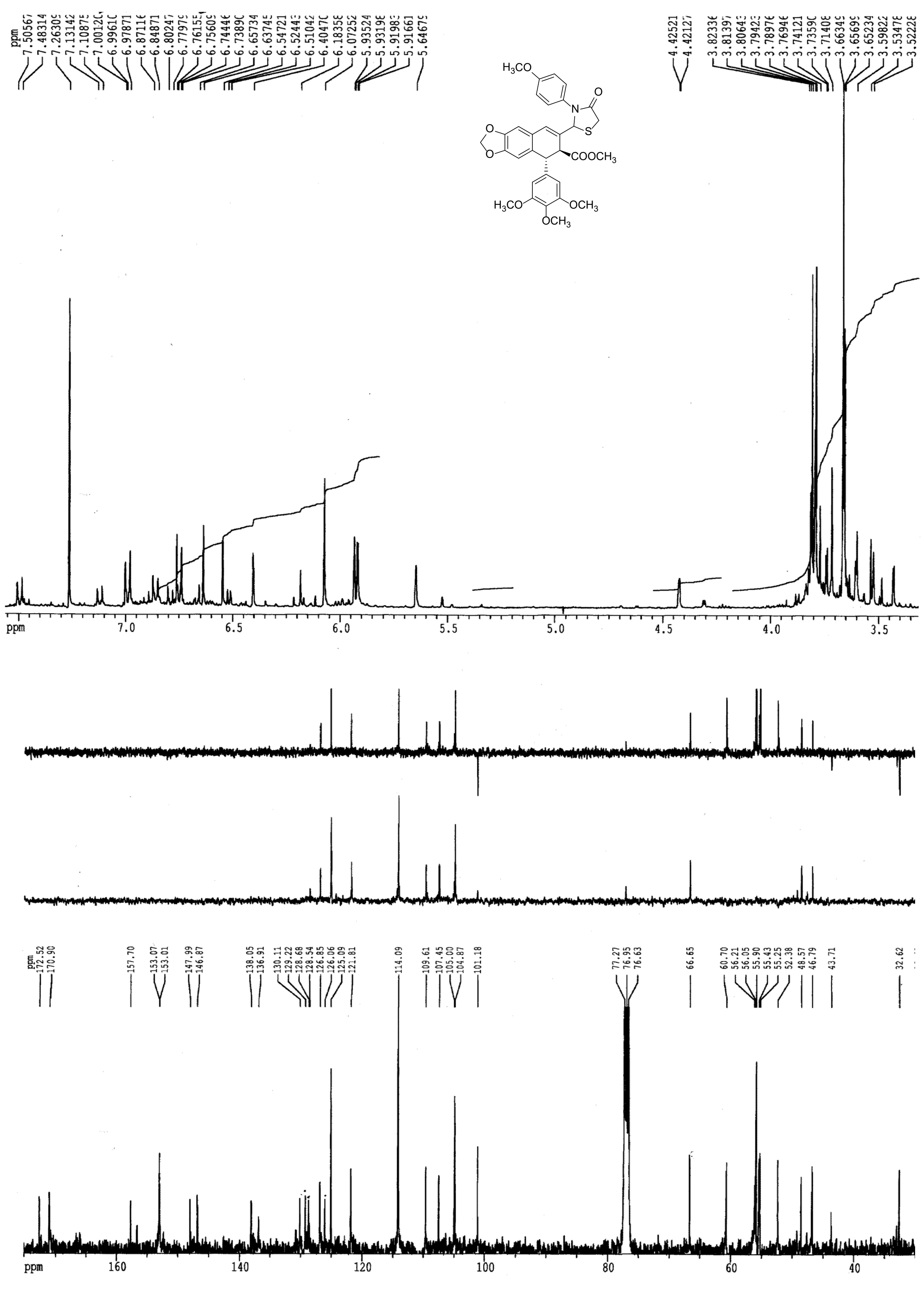

Figure S43. ^{1}H and ^{13}C for compound **32**

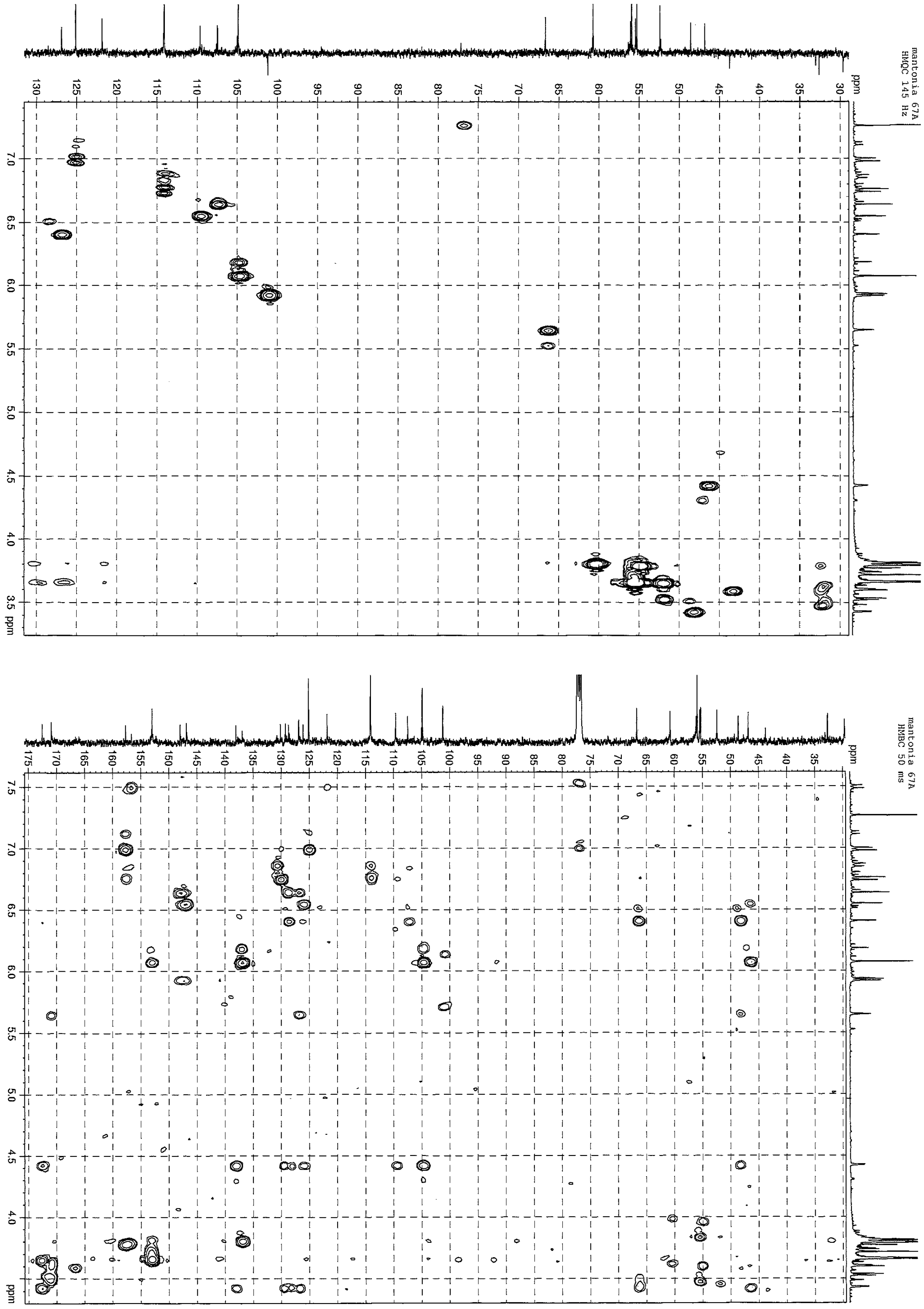

Figure S44. HMQC and HMBC for compound **32**
